# The ESA Parastronaut Feasibility Project: Investigating the Need and Contents of Physical Performance Tests for an Inclusive European Astronaut Corps

**DOI:** 10.1007/s40279-023-01891-4

**Published:** 2023-09-25

**Authors:** Isabella Wiedmann, Guillaume Weerts, Klara Brixius, Anna Seemüller, Justin Mittelstädt, Nolan Herssens, Tobias Weber

**Affiliations:** 1 Institute for Cardiovascular Research and Sports Medicine, German Sports University Cologne, Cologne, Germany; 2 KBR GmbH, Cologne, Germany; 3 Institute of Aerospace Medicine, Aviation and Space Psychology, German Aerospace Centre, Hamburg, Germany; 4 Space Medicine Team (HRE-OM), ISS Operations and Astronauts Group, European Astronaut Centre, Directorate of Human Spaceflight and Robotic Exploration, European Space Agency, Linder Höhe, 51147 Cologne, Germany

## Abstract

**Introduction:**

In 2022, the European Space Agency (ESA) held the first astronaut selection since the beginning of space flight that allowed physically impaired astronaut candidates to be selected in an inclusive European astronaut corp. The main objective of the ‘parastronaut feasibility project’ is to investigate if physical performance tests (PPTs) should be part of future astronaut recruitments for an inclusive ESA astronaut corps to test their flight readiness. The objectives of this study are (1) to assess if future (para-)astronaut recruitment campaigns should include PPTs to ensure flight readiness, safety, and mission success; (2) if so, which areas of physical performance should be tested to mimic nominal and off-nominal crew activities during all phases of a space mission; and (3) to assess whether PPTs are compatible with the ethical principles of equal opportunity for an inclusive pool of astronaut candidates.

**Methods:**

58 subject matter experts with specialisations in space physiology, operational human space flight, space medicine, medical ethics or parasports were interviewed in two rounds using the Delphi method. Both qualitative and quantitative data were obtained, analysed, categorised, and visualised using the qualitative research tool NVivo and Excel.

**Results:**

Two thirds of the experts were in favour of adding PPTs to future astronaut selections and recommended to implement them for both physically unimpaired and physically impaired astronaut candidates. The main physical skills that should be examined are space-related, mission-specific coordination skills of the upper extremities, followed by endurance performance and stamina, dexterity of the upper extremities, motor learning ability and mobility.

**Conclusion:**

Based on this study, it is clear that PPTs should be part of future astronaut selection campaigns. However, the content of these PPTs must be carefully evaluated and validated using existing data on crew activities before, during, and after space flight, while considering equal opportunities in the context of human space flight. Historical considerations have influenced current astronaut requirements, but this study's findings indicate a need to reassess these requirements for future inclusive selection campaigns, as their validity and necessity remain uncertain.

**Supplementary Information:**

The online version contains supplementary material available at 10.1007/s40279-023-01891-4.

## Key Points

**Table:** 

Physical performance tests were deemed to be a useful tool to assure mission readiness during future astronaut selections for both physically impaired and physically unimpaired astronaut candidates
The pool of physically impaired subject matter experts and experts working in the field of parasports are strong advocates for physical performance tests giving physically impaired astronaut candidates an opportunity to demonstrate that they are fully capable crew members
Physical performance tests for astronaut candidates should mainly be used to assess physical performance outcomes of the upper extremities

## Introduction

In the spirit of diversity and inclusion [1], David Parker, Director of Human and Robotic Exploration of the European Space Agency (ESA), announced in March 2021 that the 2021 ESA astronaut selection would be the first astronaut selection in the history of human space flight to include astronaut candidates with physical impairments [2]. To this date, human space flight has exclusively been available to physically and psychologically unimpaired individuals [2, 3]. This implies that thus far ableist logics have indeed been applied through all previous astronaut selection campaigns. ESA’s Parastronaut Feasibility Project should therefore be regarded as the first attempt to break with this logic.

As the environment found in space is characterised by a high vacuum (1.33 Pa), microgravity (< 1 µg), extreme temperatures (+ 100 °C to − 100 °C), meteoroids, space debris (> 12,000 catalogued debris objects larger than 10 cm with orbital speed of 8–70 km/s), ionospheric plasma (spacecrafts can develop an induced charge up to − 140 V), ultraviolet, especially shortest wavelength, ionising (425 µSv/d) and non-ionising radiation [4], circadian desynchrony (light–dark transition every 45 min on low Earth orbit), and increased acoustic noise in the spacecraft (ambient noise at International Space Station [ISS]: 60 dBA) [5], it is considered to be extremely hostile and poses great challenges to its explorers. Thus, over the years, test scenarios have been developed and adapted to ensure excellent medical and mental capabilities of the astronauts [6–8], aimed at safeguarding all crew members during all activities inside and outside of the space vehicle to eventually ensure mission success.

With the start of the ISS programme, medical standards were developed by the ISS Multilateral Medical Operations Panel with the aim of ensuring that all crew members involved in an ISS mission meet these standards to assure their safety and mission success [9]. During an astronaut's professional life, the requirements change periodically, depending on whether an astronaut is in mission preparation, in the launch or landing phase for a space mission, in a long-term or short-term mission to the ISS, or returning to other duties and tasks within the agency between missions or after the final mission. Due to the trend towards long-duration missions, these medical requirement standards have been adapted over time and are now individually tailored to the profile (e.g., pilot, mission specialist) of the crew member [9, 10]. During previous ESA astronaut selections, physical performance capabilities (i.e., individual readiness to perform nominal and off-nominal mission operations) evaluated through physical performance tests (PPTs) have not been part of the selection criteria.

In the past, astronaut candidates with any form of physical impairment have been excluded from the selection process due to general safety reasons [2, 7, 11]. However, ESA’s Parastronaut Feasibility Project [2] seeks to explore if individuals with physical impairments are also capable of fulfilling the role of an astronaut similar to any other crew member without physical impairments.

The following study uses the terms ‘para’ and ‘physical impairments’. The authors would like to highlight that these terms do not cover the broad spectrum of individual conditions and their implications for the individual lives of affected humans. This implies that these terms cannot be used in a one-fits-all way as every physical impairment and its consequences are unique and affect each individual’s life differently. Every time the terms ‘para’ and/or ‘physical impairment(s)’ is mentioned, the authors are referring to the International Classification of Functioning, Disability and Health (ICF) of the World Health Organisation (WHO) [12, 13].

Considering the above, the main aims of the present study are to explore.

1. if PPTs should be added to future inclusive astronaut selection campaigns to assure flight readiness of astronaut candidates with physical impairments;
2. if implementing additional PPTs for physically impaired astronaut candidates is in line with the ethical principle of equal opportunities, or if all members of an astronaut corps should undergo these additional tests;
3. which physical performance outcomes should be included in a PPT, should a PPT be deemed necessary for future inclusive astronaut selection campaigns.

## Methods

A classic two-stage Delphi survey was conducted and implemented as an online version (see Supplement 1, 2 and 4 in the electronic supplementary material [ESM]), following an exploratory approach in terms of questionnaire construction and result analysis [14], to capture the knowledge and opinions of a panel of experts familiar with the challenges of living and working in microgravity. The first round of the Delphi Survey was conducted during 18–26 January 2022 and the second was conducted during 2–9 March 2022. This project, with the reference number 156/2021, was approved by the ethics committee of the German Sports University, Cologne.

### Expert Selection

**Fig. 1:**
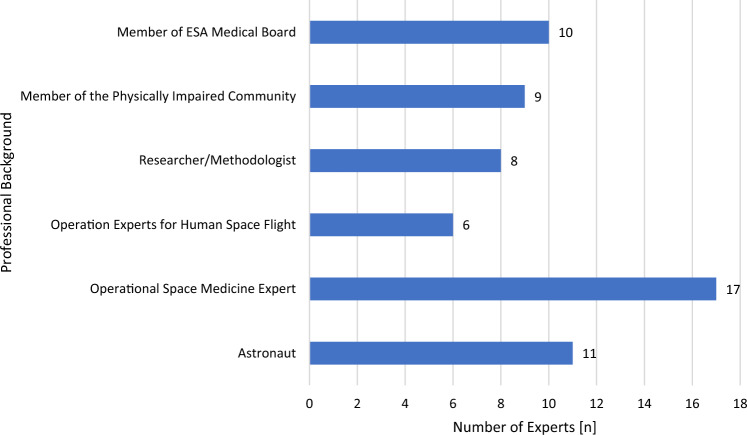
Expert panel composition

The inclusion criteria to determine the eligibility of experts for this two-stage Delphi survey [14] required senior professional expertise (working as a professional in a field for at least 5 years) in at least one of the following fields: space physiology, aviation and space medicine, space-related science or space-related exercise science, human space flight operations, parasports or living as a person with a physical impairment working in a high-performance sector, medical ethics, being an astronaut with at least one mission and a total work experience of at least 5 years. As the recommendations for an appropriate panel size has a range of 8–500 [15], we identified 61 participants eligible for the present expert panel. Out of these experts, six subgroups (based on their individual expertise and background) were formed, and each subgroup had to consist of/include a minimum of 15% of experts with a physical impairment. The expert panel consisted of 55 experts from ESA member states, whilst six experts were from USA, Canada and Australia. A detailed breakdown of the expert panel composition and their professional backgrounds is presented in Fig. 1. Based on the work of Gordon [16], a dropout rate of 35–70% was expected. All members of the expert panel were contacted via email that contained an explanation of the study and a link to their individual survey (see Supplement 1 in the ESM) and were given 7 days to answer in each round. Of note, the expert panel composition is only valid for the first Delphi round since the survey for Delphi round 1 included a specific question for the expert panel to confirm that the professional background assigned to them was correct. In the following Delphi round this question was not asked, thus the composition of the expert panel might vary from the initial expert panel composition as depicted in Fig. 1.

### Design of the Delphi Survey

One of the strengths of the Delphi survey is that, through the initial use of qualitative techniques, large amounts of data can be collected according to a systematic, rule-guided procedure and analysed in a highly specific way, geared to a concrete research object. By prioritising the appropriateness of the subject matter and not applying the systematics, coding rules and semantic principles in a rigid and inflexible form, a multitude of opinions can be depicted [17–20].

The Delphi survey was conducted in two rounds. The questionnaire used for the first round of this Delphi Survey focused on the benefits as well as advantages and disadvantages of a PPT during astronaut selection campaigns for physically unimpaired and physically impaired astronaut candidates, while for the second round the importance of different motor skills in different career or training stages of an astronaut were evaluated. In order to improve the quality indicators of the questionnaires, safety guidelines for operation of the ISS (i.e., “ISS Crew Transportation and Services Requirements” document [21] and “International Space Station Flight Crew Integration Standard” [22]) were examined in preliminary preparation work for their practical significance. The questionnaires were constructed based on the current safety standards and the requirements of ESA’s Space Medicine Office [2]. Based on the recommendations by Keeney et al. (2006) [18], it was decided for the current survey that two rounds of expert interviews and the associated pertinent recirculation of the survey would be sufficient to address the main objectives of the present study.

The questionnaire used for the first round (see Supplement 2 in the ESM) consisted of single-choice questions. Participants were encouraged to give an additional free-text answer explaining their response [23–25]. In total, the first Delphi round consisted of nine questions divided into four categories. The first category examined whether the experts generally expected benefits of PPTs during future inclusive astronaut selection campaigns to ensure the operational readiness of astronaut candidates. In the second category, advantages and disadvantages of PPTs for the group of physically impaired and physically unimpaired astronaut candidates were addressed. The third category evaluated the extent to which there were concerns about the use of PPTs for future astronaut selections. The last category addressed the specific physical performance outcomes that a PPT should test considering the physical demands of astronauts during all stages of their careers and missions. All answers were then analysed according to the criteria of qualitative content analysis.

According to the recommendations of Keeney et al. and Niederberger and Köberich [18, 26], results were sent to the experts for a second evaluation if a 67% consensus could not be achieved in round one in terms on the question of whether PPTs can ensure the readiness of impaired and unimpaired astronaut candidates [18, 26]. Furthermore, the experts (*n* = 58) were provided with a summary of the results of the first round at the end of the first round. This was important for further processing the questionnaire in the second round.

The questionnaire used in the second round predominantly contained closed questions (see Supplement 4 in the ESM). We used a rating scale with values ranging between 0% (i.e., not important at all) to 100%, (i.e., extremely important) in a range of 5% to assess the importance of certain physical performance skills. All surveys were conducted in written form using LimeSurvey [27]. For qualitative analysis, NVivo (released March 2020) [28] was used, and LimeSurvey and Excel were used for frequency analysis of the answers and graphical representation. Subsequently, data were paraphrased by one author and cross-checked by a second author. In cases of disagreement, the passages in question were discussed until a consensus was reached. In case of disagreement, a third reviewer was involved to reach a consensus.

### Qualitative Content Analysis

The present study was conducted using guidelines and principles for qualitative research provided by Mayring and other authors [25, 29–35] to ensure the highest rigor and trustworthiness possible. The guidelines and principles used in the present study are in line with the standards of the ‘big-tent’ model by Tracy [36] addressing the fields of ‘worthy topic’, ‘rich rigor’, ‘sincerity’, ‘resonance’, ‘significant contribution’, ‘ethical’, ‘meaningful coherence’. All expert opinions were analysed applying the principles of qualitative data analysis by Mayring [37] and Kuckartz [29, 38]. Since the aim of the present survey was to achieve consensus among the experts, the aspects of valence analysis [39] were applied, albeit under Kaiser’s [40] more moderate application criteria in order to be able to accurately represent the value of the results. After defining the selection criteria and the level of abstraction, the material was processed to determine the categories. This was followed by a subsumption with a final pass through of the material. Finally, the results were formulated. In order to improve validity and intercoder reliability, relevant text passages were reviewed by two of the authors and their results were compared with each other. A verification of semantic validity in the categorisation was also performed by two of the authors. In case of disagreement, a third author was consulted [33]. Following this step, the results were grouped according to the categories of the main tendency of the experts’ response (“Yes”, “No”, “Not sure”, “No answer”). The contents were grouped according to main statements and assigned into themes in so-called ‘super categories’ [19, 41, 42]. Based on this categorical system, the survey content was further paraphrased and summarised, narrowing down the replies to core statements and/or standpoints. Using valence analysis, up to a maximum of five of the most frequent answers or topics were determined and included in the results.

### Quantitative Analysis and Statistics

Since the core statistics in the evaluation of Delphi surveys mainly consist of the determination of the measures of central tendency and level of dispersion, as the scale level allows, a per-protocol analysis was conducted in each round [19].

In the first Delphi round, the answers (“Yes”, “No”, “Not sure”, “No answer”) for the respective questions were analysed for absolute and relative frequency. The second set of questions of the first Delphi round consisted of answers that could be selected from a predefined list (“I don’t think specific skills need to be tested to ensure readiness during deployment training”, “Not sure”, “The following skills should be tested…”) and a comment field for free answers. The open-ended responses were analysed for frequency and central response tendency after the paraphrasing and explication analysis steps.

In the second Delphi round, the statements of the percentage-scaled rating scale were evaluated according to absolute frequency and measures of central tendency. The experts were asked to assess the importance of certain physical performance skills during an astronaut’s career. The different career stages were defined as follows:

1. Astronaut Recruitment Process
2. Astronaut Basic Training
3. Mission Preparation
4. Launch
5. In Flight
6. Landing Process
7. Post Flight

The current ranking scheme is an adapted system after the principles of Hopkins [43]. The mean values were evaluated individually for the assumed importance of each skill during the individual career phases of an astronaut. Three levels of prioritisation were established for the different skills. Values above 81% are essential. Prioritisation level 1 (very important) has a value range of 61–80% importance. For prioritisation level 2 (moderately important) a value range of 50–60% applies and for prioritisation level 3 (negligible) a value range < 50% applies.

## Results

### Expert Characteristics

**Fig. 2:**
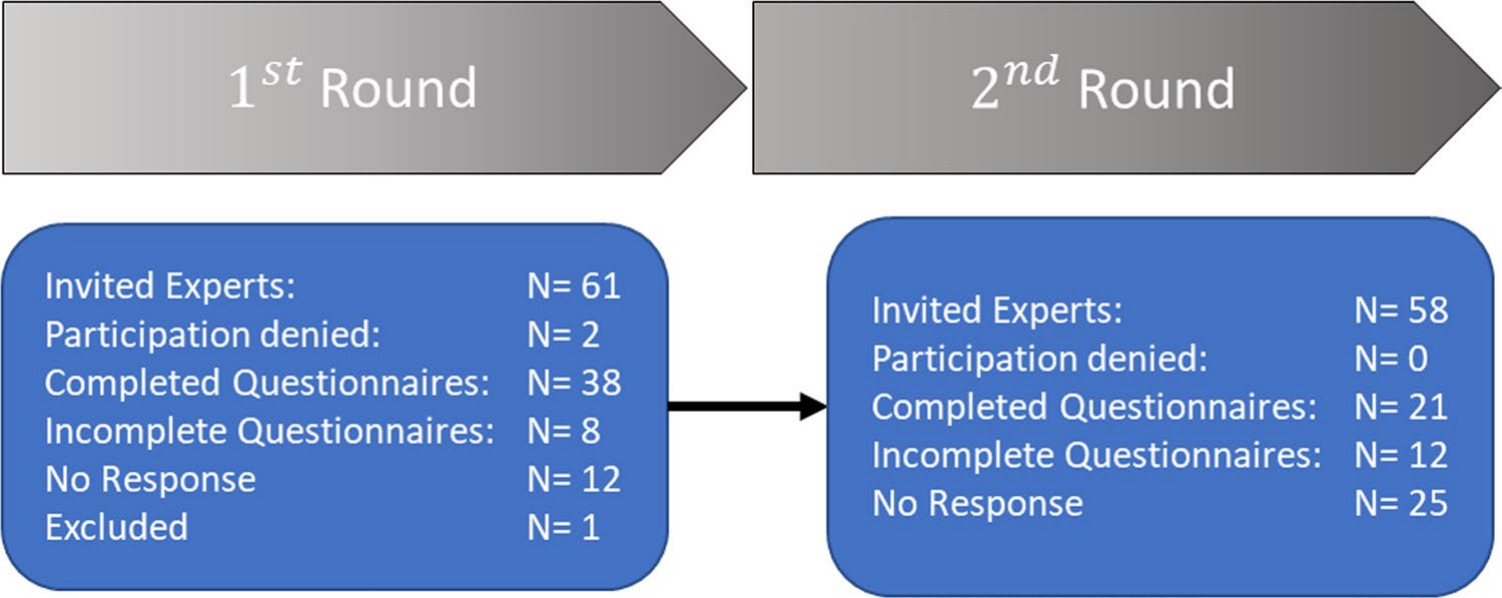
Expert participation in the Delphi Survey (*n* = 61)

From the 61 invited experts, 58 experts with six different professional backgrounds finally participated in the expert panel of the Delphi survey as two refused participation and one had to be excluded due not meeting the inclusion criteria (Fig. 2).

On average, the panel had 18.0 ± 11.1 (max 40.0; min 6.0) years of experience in their profession. In the first round we received 38 and in the second round 21 fully completed questionnaires (Fig. 2). The dropout rate for the first Delphi round was 23 (relative dropout rate 38%: 2 denied participation, 8 incomplete questionnaires, 12 did not respond, 1 was excluded due to insufficient work experience). For the second round the dropout rate was 27 (relative dropout 64%: 12 incomplete questionnaires, 25 did not respond). Due to the applied blinding process, it was not possible to specifically invite members of the expert panel for Delphi round two who participated in Delphi round 1, instead the invitation to participate in the second Delphi round was sent to all experts initially identified for Delphi round 1, expect for those experts who were excluded (1) or denied their participation (2).

The final expert panel consisted of members of the ESA Medical Board (*n* = 10), members of the physically impaired community or persons regularly working with physically impaired individuals (*n* = 9), life scientists or methodologists (*n* = 8), operational experts in human space flight (*n* = 6), operational space medicine experts (*n* = 16) and active or former members of the ESA astronaut corps (*n* = 9).

### Added Value of a Physical Performance Test (PPT) for Future Astronaut Selections

#### General Astronaut Candidate Population

**Fig. 3:**
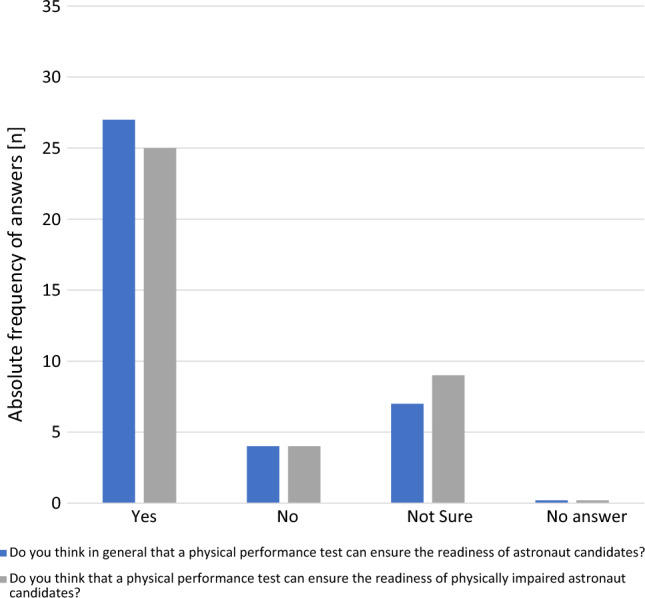
Absolute frequency of answers (*n* = 38) on general benefits of physical performance tests in physically unimpaired and physically impaired astronaut candidates

Figure 3 shows the quantitative results regarding the general questions whether a PPT ensures the operational readiness of astronaut candidates. 71% (27/38) of the experts stated that a PPT is beneficial to ensure operational readiness for astronaut candidates.

Additional general benefits of PPTs for astronaut candidates were seen in the following three areas:

- objective monitoring of individual performance,
- improvement of team performance,
- and as a long-term effect, health monitoring and training control.

Most experts reported that PPTs would be a suitable tool for ensuring operational readiness for astronaut candidates in general (71.1%) (Fig. 3). The main reasons provided in the open answers to these questions were that PPTs could ensure management of emergencies and survival during extreme (off-nominal) emergency situations. As good physical performance is also considered helpful during times outside mission-specific training and space missions, PPTs can provide indications of a healthy lifestyle and enable the personnel in charge to take supportive measures at an early stage to counteract developments that could endanger operational readiness. Furthermore, PPTs were deemed suitable to ensure operational readiness considering age and gender differences provided the measured PPT outcomes would be evidence-based and job-specific.

A PPT as a tool for ensuring operational readiness for astronauts in general was rejected by 10.5% of the experts (Fig. 3) due to the opinion of some experts that physical performance is not the only determining factor for successful mission operational readiness. In addition, results achieved in PPTs would only reflect a momentary picture with no long-term significance or prognostic value.

Seven of the 38 experts (18.4%) were unsure about the added value of a PPT during astronaut selection campaigns, stating that there are many aspects other than performance that contribute to the success of a mission.

#### Physically Impaired Astronaut Candidate Population

Physical performance tests were also considered as a suitable tool to ensure operational readiness in physically impaired astronaut candidates (agreement by 65.8% (24/38) of the experts).

The experts identified the following areas as added value:

- comparability of individual physical performance with the performance of physically unimpaired astronaut candidates;
- confidence building in the team through evidence of equal physical performance capabilities, and improved monitoring of the impact of physical impairment;
- PPTs can be used to test existing supporting hardware to inform mission and hardware designers about usability of current equipment and needs and requirements for new supporting hardware solutions for physically impaired crew in space.

The main reasons for an added value of PPTs in physically impaired astronaut candidates were (1) to ensure operational readiness provided PPTs would specifically test job-specific physical requirements; (2) to ascertain if PPTs would need to be individually tailored to the requirements of individual impairments; and/or (3) to assess if the type and degree of physical impairment is compatible with human space flight.

It was also suggested that medical supervision should be provided during the performance of the PPTs. Another reason for experts advocating for PPTs for physically impaired astronaut candidates was that a person with a physical impairment is also expected to demonstrate their ability to perform the same astronaut-specific tasks as an individual without physical impairment. Furthermore, it was assumed that individuals who apply as a parastronaut can cope very well with their impairment and would expect PPTs to be part of the recruitment process. The experts also indicated that PPTs could be used to assess other performance variables, not directly linked to physical performance, such as mental fitness and resilience.

Implementation of PPTs for physically impaired astronaut candidates was rejected by 10.5% of the experts. The main reasons included the concern that there would be problems adapting the test design, and that the environment cannot be fully adapted to people with physical impairments in such a way that the predictive value of a PPT for astronaut candidates with physical impairments is comparable to that of physically unimpaired people. Furthermore, there were concerns that the testing procedures were not sufficiently fair, evidence-based, and would include restrictive parameters. Importantly, it was recommended by this group that the use of individual assistive devices should be allowed for people with physical impairments.

Nine out of 38 experts (23.7%) were unsure whether a PPT adds any value to assess operational readiness of physically impaired astronauts as these would only be useful if they capture task- and job-specific skills. Furthermore, it was a dominant opinion that physical performance can be trained to a certain extent and therefore the design of a PPT should not be overly focussed on the parameters of strength or endurance skills, but rather on motor skills.

### Advantages and Disadvantages of a PPT

**Fig. 4:**
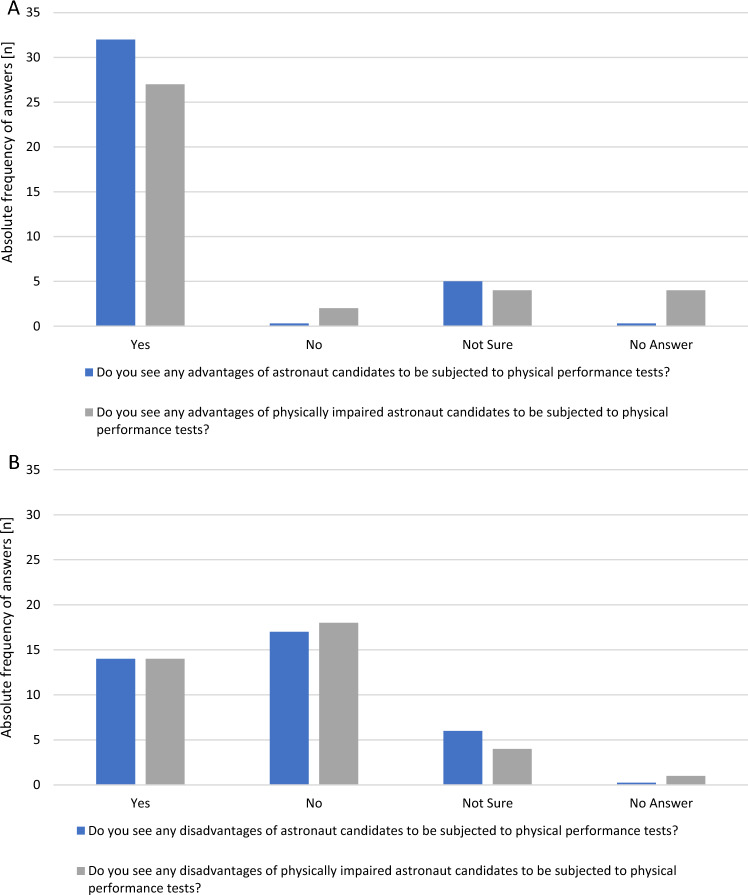
Advantages (**A**) and disadvantages (**B**) of physical performance tests for physically unimpaired and physically impaired astronaut candidates (*n* = 38)

The advantages and disadvantages of PPT for physically unimpaired and for physically impaired astronaut candidates are displayed in Fig. 4.

Most experts stated that the use of PPTs in both physically unimpaired (84.2%) and physically impaired (71.1%) astronaut candidates would be advantageous to assess mission readiness (Fig. 4A).

The main advantages were seen in the following three areas:

- objective monitoring of individual performance,
- improvement of team performance,
- and as a long-term effect, health monitoring and training control.

Applied to physically impaired candidates, additional benefits were seen in the areas of

- comparison of individual performance with the performance of physically unimpaired astronaut candidates,
- confidence building in the team through evidence of equal physical performance capabilities of astronaut specific tasks,
- and improved monitoring of the impact of a physical impairment.

The study found that 36.8% of the experts assessed PPTs to be disadvantageous for both physically impaired and unimpaired astronaut candidates (Fig. 4B) and 15.8% of the experts were unsure about possible disadvantages in terms of unimpaired candidates and 10.5% for the impaired candidates. With respect to physically unimpaired astronaut candidates, the main disadvantages reported were the physiological and psychological stress caused by a PPT, the increased risk of injury, and the risk of exclusion of potentially suitable candidates due to inadequate physical performance. A further disadvantage of implementing PPTs for physically impaired astronaut candidates was seen in the limited transferability from valid tests for unimpaired to people with physical impairments.

### Aspects of Ethics and Equal Opportunities

As the use of testing does involve risks of discrimination, the survey also focused on ethical aspects and equal opportunities.

**Fig. 5:**
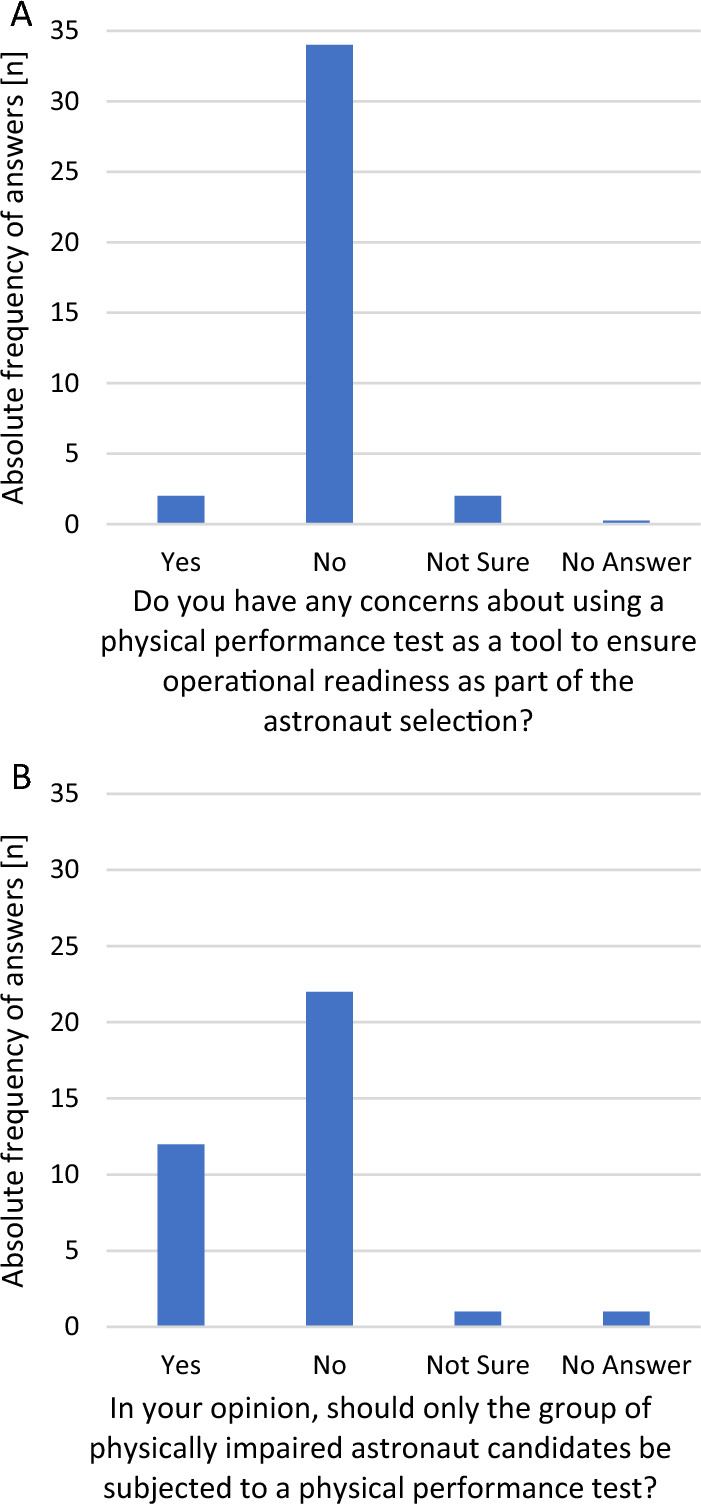
**A** Concerns about using a physical performance test as an assessment tool to ensure operational readiness as part of astronaut selection (*n* = 38). **B** Exclusive physical performance tests for physically impaired astronaut candidates (*n* = 36)

Regarding the question of using PPTs as a tool to ensure readiness for nominal and off-nominal mission operations in astronaut candidates, 31.5% of respondents expressed their concerns around implementing PPTs, 57.8% (22/38) had no doubts of using PPTs, one person (2.6%) was not sure and three people (7.9%) abstained (Fig. 5A).

In their additional answers the experts provided a wide range of reasons for their skepticism and uncertainty to implement PPTs in future inclusive astronaut selection campaigns. The main concern was that overly rigorous thresholds with respect to ability level testing would result in the loss of potentially suitable applicants. Therefore, the experts recommended that tests should be validated against the required astronaut-specific skills. Additional concerns were raised that the use of PPTs risked testing performance rather than aptitude.

Most of the experts (89.4%) (34/38) rejected exclusive testing of physically impaired astronaut candidates using PPTs (Fig. 5B). The main arguments were that exclusive testing of physically impaired astronaut candidates is discriminatory and violates the principle of equal treatment.

In two cases (5.3%), exclusive testing of physically impaired astronaut candidates was advocated, arguing that individuals with physical impairments should be able to demonstrate their physical capabilities to the same extent as unimpaired individuals.

In 5.3% (2) of cases, the experts were not sure about the added value of testing only candidates with physical impairments, as all crew members should have a reasonable level of fitness. As the test conditions may change if participants have a particular physical impairment, it was not clear to them whether individual testing is required for each individual physical impairment.

### Physical Performance Skills During an Astronaut’s Career

To specify which test procedures might be suitable to ensure nominal and off-nominal mission operation readiness, the importance of individual physical performance skills during an astronaut's career at specific career stages had to be evaluated. However, it must be considered that not every physical performance skill has the same importance at every stage of an astronaut’s career but can nevertheless be important for the overall risk management of a space mission.

**Fig. 6:**
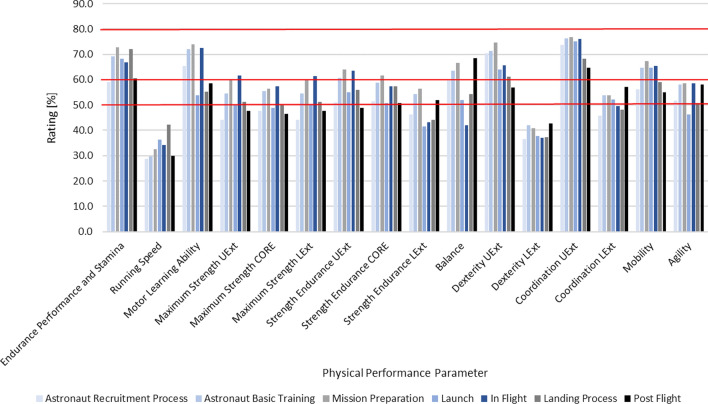
Mean percentual rating of the importance of 16 sub-items on a rating scale of 0% to 100% for the seven different stages of an astronaut’s career. The prioritisation is assigned in three different levels: level 1 (very important) has a range of 61–80%, level 2 (moderately important) a range of 50–60% and level 3 (negligible) a range for everything < 50%

Figure 6 shows the mean percentual rating on a rating scale with the range 0–100% of 16 different performance parameters considered important for different stages of a successful astronaut career. The different items have been rated by the expert for seven different career stages and are displayed clustered according to their affiliation. The prioritisation is assigned in three different levels—level 1 (very important) has a range of 61–80%, level 2 (moderately important) a range of 50–60% and level 3 (negligible) comprises everything < 50%.

Of the 16 items that the experts rated, five items were assigned to level 1 (very important), eight items to level 2 (moderately important), and three items to level 3 (negligible). As depicted in Fig. 6, the experts rated coordination of the upper extremities as the most important physical performance parameter. This is followed by endurance performance and stamina, dexterity of the upper extremities, motor learning ability and mobility, all predominantly assigned to level 1 as very important to assure crew safety and mission success. The experts predominantly classified strength endurance of the lower extremities, dexterity of the lower extremities, and running speed as negligible physical performance parameters (level 3). The eight remaining parameters of balance, strength endurance of the upper extremities, strength endurance of the core, agility, maximum strength of the upper extremities, core and lower extremities and coordination of the lower extremities were ranked as moderately important (level 2).

## Discussion

The present study sought to collect and consolidate subject matter expert opinions to explore whether PPTs should be part of future inclusive astronaut selection campaigns to ensure operational readiness of physically impaired astronaut candidates. In the first survey round, the expert panel’s unambiguous opinion was that a PPT is in principle suitable to ensure the operational readiness of both physically unimpaired and physical impaired astronaut candidates.

Another main finding is that most of the experts were against using PPTs exclusively for physically impaired astronaut candidates. Obviously, exclusive testing of physically impaired candidates was rejected on the principle of discrimination, although it is acknowledged that the unrestricted transfer of all PPTs suitable for unimpaired candidates for use in physically impaired candidates may not necessarily be applicable for every scenario and individual testing may become necessary.

In this context we would like to highlight the following valuable statement from one of our ethicists:

*Physical performance tests should not be the only characteristic, even for impaired astronaut candidates. But the difficult question is whether to use the same tests, or adapted tests that take into account the disability in question. It must also be considered whether (medical) technical aids that can partially compensate for the disability should be taken into account (or whether one test with and one test without should be performed). Furthermore, a threshold value will again be required, which is assumed to be reached in order to be able to avert dangers to oneself and others (as far as realistic). Using the same test may be fair to all candidates (impaired and not-impaired), but may be unfair to impaired candidates who may have other skills that are valuable.*

*Nevertheless, fairness alone cannot be decisive in view of the mentioned duty to be physically able to meet the minimum requirements in order to minimize risks (in general and especially in emergencies). Especially in the case of tests for impaired candidates, it seems particularly important that they are as evidence-based as possible and thus do not make excessive demands, but rather test what is actually required. A test that requires something that an impaired candidate can only achieve with difficulty, but which is also not absolutely necessary (to achieve this with this performance), but rather is based on prejudices or (not always realistic) ideal conceptions of an astronaut, would be unfair and could be discriminatory without justification.*

The statement above elegantly summarises the challenge between trying to be truly inclusive (i.e., allowing all physically impaired individuals access to space) and the operational reality that requires a certain level of physical performance capability to assure self-protection, protection of fellow crew members, as well as the preservation of on-board equipment.

Interestingly, most of the physically impaired community clearly expressed that they were in favour of implementing PPTs in future inclusive astronaut selection campaigns to demonstrate their overall ability to perform nominal and off-nominal crew-specific tasks, and to show that their physical impairment should in no way be considered a deficit that poses risks or limitations to performing their work as a fully integrated and capable crew member as can be seen in the statements of the members of the physically impaired community below:

*“No para candidate wants to be baggage. They will want and need to be a vital member of the team. Physically testing them shows they are expected to pull their weight.”*

and

*“Candidates applying for the Parastronaut Programme as an achiever type subgroup of impaired persons will be more than proud and willing to show in how far they cope with their impaired condition. They would most probably expect such a test, because this is where they are good at and stand out from the impaired 'crowd'.”*

and

*“They will expect to be asked to show their level of self-sustained living, also involving making up for their disabilities.”*

Since this survey also addressed the question of which priorities should be set within the test battery, the answers received in the first round and in the second round allowed a very clear prioritisation towards functional tasks focussing on strength endurance and motor skills of the upper extremities. Prioritisation of individual physical performance skills in the second round showed that the highest importance was assigned to coordination of the upper extremities, endurance performance and stamina, dexterity, and in certain phases of the astronaut career, to motor learning ability, mobility, and balance. The lowest priority was allocated to running speed and dexterity of the lower extremities.

Accordingly, the results of this survey confirm the justification of narrowing down the selection criteria of lower limb impairment and short stature within this first pioneering selection campaign of the Parastronaut Feasibility Project, since impairment of these functions can be expected to have a lower impact on the overall performance of the astronaut on orbit, as in microgravity both tasks and navigation through the vehicle are done using the upper extremities [44]. Furthermore, this survey’s relevance is clearly demonstrated by the fact that in the first round, 32% of the expert panel expressed various concerns in terms of using PPTs. This also applied in the second round, which addressed the extent to which the experts would still miss skills during the Delphi survey that would have to be tested in the context of a PPT. Here, 62.5% of the responses demonstrated a need for further discussion. This suggests that this topic has not been exhaustively discussed yet.

As in many other technically oriented professions, human space flight has traditionally been dominated by physically unimpaired male individuals [8, 44], and still is to this day [45, 46]. Therefore, it is important not to introduce a gender or selection bias for truly inclusive future astronaut selection campaigns in a field which has certain commonalities with the military, fire fighters and police services [47–49], by looking at past testing procedures [47–49]. For a genuinely inclusive astronaut selection campaign it will be important to critically review the criteria that were used during previous selection campaigns and to revise those criteria that inherently introduce a selection bias that will inevitably lead to a skewed representation of the general population of future crews.

The question of whether the use of PPTs is ethically correct can only be answered for some areas by the survey obtained. Although the experts are clearly convinced of the benefits of PPTs, the answers also reflect the concern that the principles of equal opportunities may be violated under certain circumstances. Nevertheless, the question arises as to what extent it is ethically justifiable not to use a PPT in the event an emergency occurs that poses a risk that a person with a physical impairment may not be able to perform certain off-nominal rescue manoeuvres, and thus self-protection, protection of fellow crew members, as well as the preservation of on-board equipment might be jeopardised.

Typically, medical mission readiness is evaluated under standardised laboratory or field conditions [48], which is difficult to simulate for an environment like microgravity. This becomes even more challenging when implementing PPTs since no previous experience has been gathered in this field.

Based on the results of the Delphi rounds, pre-existing test batteries and test batteries from related disciplines have been analysed for their possible transferability using strategies from the field of occupation-specific research [48, 50]. Since the experts in their assessment very clearly emphasised upper extremity skills during basic training, mission preparation and during the in-flight phase, including the work/job-specific requirements, it seems necessary to evaluate what conditions the astronauts are exposed to in these periods [44].

For example, astronauts very often have to wear various forms of protective clothing (scuba diving equipment, extravehicular mobility unit [EMU]/Orlan suit) weighing up to 145 kg (EMU suit) [51, 52] with partly limited mobility, tactile perception and vision. During the mission preparation, attempts are made to reduce this load for the astronauts. Nevertheless, even a partial pressure suit can weigh about 6 kg (SpaceX IVA Suit, ‘Starman Suit’; Boeing, ‘Starliner Spacesuit’) to 10 kg (Advance Crew Escape Suit, ‘Pumpkin Suit’) [53]. For this reason, it is advisable to test the necessary motor skills under these very specific circumstances.

According to the study by Petersen et al., 2015 [50], the Astronaut Fitness Assessment (AFA), including the measurement of height, body mass and body fat percentage, hip flexion/extension, muscle strength (handgrip, trunk strength and one repetition maximum [1RM] for squat and bench press), double-legged jumps and balance parameters on the pressure plate seem to be a suitable test battery for professional astronauts for the operational implementation of field tests for human space flight. However, the tests presented by Petersen et al., were designed for physically unimpaired astronauts; thus, if parts of the AFA tests are to be used for future PPTs, these tests would need to be individually tailored for crew with physical impairments.

Several systematic reviews [49] examining the occupational skills of individuals in police, fire brigade, army, and air force sectors through fitness assessments appear to have limited relevance and utility when assessing the requirements profile of astronauts. However, this only partially reflects the results of the present study. Considering that firefighters routinely perform their duties while wearing similarly heavy protective equipment, it might be worth exploring the potential adaptation of functional tests used in this field, such as the Trondheim Test for Experienced Firefighters developed to meet functional requirements in Norway [47, 54], to assess the job-specific requirements of astronauts.

## Study Limitations

The transferability of qualitative research results is highly dependent on the analytical skills of the researcher [17] and the interpretation approach or external opinions, for example, on the extent to which spacecraft or equipment can be adapted for people with impairment [55]. And of course, a closer examination of individual expert viewpoints always carries a certain risk of introducing subjectivity and bias. Moreover, averaged expert opinions, especially those linked to closed questions or questions that only allow a certain degree of freedom could possibly restrict new approaches and introduce bias. Although predefined survey forms or response mechanisms were used throughout the study, a noticeable dropout rate was evident as participants often did not complete the questionnaires to the end. Non-respondents were not followed up.

Regarding the technical procedure, it became apparent that, despite multiple pilots of each round, there was often a lack of understanding, misunderstanding, or technical difficulties with some individuals. Possibly, an even larger group of people should be included in such pilot studies in the future. Finally, there was a significant number of dropouts in the present study; however, a dropout rate of 35–70% is to be expected in Delphi surveys as investigated by Gordon [16]. Thus, the present dropout rate after the second Delphi round of 66% is still within the range that previous research has shown to be expectable and acceptable.

## Conclusion

The present study provides new insights into a field of inclusion that has never been explored before. Previous astronaut selections have always applied ableist principles and individuals with any kind of physical impairment have not been considered in this process by default. The main goal of the present study was to explore if individuals with physical impairments should be treated differently during the astronaut selection to test their physical capabilities to demonstrate their mission readiness and to ensure their own health and safety and the health and safety of their fellow crew members. Even though ESA’s Astronaut Feasibility Project seeks to break with the logics of ableism, the main priority for human space flight operations remains crew health and safety. This implies that genuine inclusivity can never be achieved in the context of human space flight. Considering the above, the authors would like to emphasise that the present study explored only three aspects of the complex and multi-layered topic of inclusion seeking to assess the value, ethics and content of PPTs for future inclusive astronaut selection campaigns. Based on the results of the present study, it is evident that PPTs should be included in future astronaut selection campaigns; however, the content of these PPTs needs to be carefully evaluated and validated using existing data on nominal and off-nominal crew activities during pre-, in- and post-flight, as well as considering all aspects of the principles of equal opportunities within the boundary conditions of human space flight. The present findings reveal that knowledge about actual physical performance skills needed for risk management is still quite limited. Since the number of people who have flown to space is comparably low and mission profiles are constantly evolving, it is currently not possible to conclusively clarify which physical performance skills, or which minimum physical performance requirements must be fulfilled to assure crew safety and mission success. Many existing requirements for astronauts are maintained due to historical considerations from the early days of space flight, and the findings of this study suggest that these historical requirements should be revisited for future inclusive astronaut selection campaigns, since it is not entirely certain whether these requirements are valid and necessary.

### Supplementary Information

Below is the link to the electronic supplementary material.

Supplementary file1 (DOCX 6378 KB)

## Declarations

### Data availability statement

All raw data supporting the results of this article can be found in the electronic supplementary material (see Supplement 1–5).
