# Supplementary material for: The ESA Parastronaut Feasibility Project: Investigating the Need and Contents of Physical Performance Tests for an Inclusive European Astronaut Corps
Source: Sports Med. 2023 Sep 25;53(11):2267–80. doi: 10.1007/s40279-023-01891-4 (PMC10587332; doi:10.1007/s40279-023-01891-4)
Supplement: Supplementary file 1 — Supplementary file1 (DOCX 6378 KB) [file 40279_2023_1891_MOESM1_ESM.docx]

The ESA Parastronaut Feasibility Project – Investigating Need and Contents of Physical Performance Tests for an inclusive European Astronaut Corps

Isabella Wiedmann^1,2,4^ID 0000-0002-9780-2688, Guillaume Weerts^4^, Klara Brixius^1^ID 0000-0003-2574-7517, Anna Seemüller^3^, Justin Mittelstädt ID 0000-0002-8419-6842^3^ Nolan Herssens^4^ID 0000-0003-0074-5814, Tobias Weber^2,4^ID 0000-0002-3015-3468

1 German Sports University Cologne, Institute for Cardiovascular Research and Sports Medicine, Cologne, Germany

2 KBR GmbH, Cologne, Germany

3 Institute of Aerospace Medicine, Aviation and Space Psychology, German Aerospace Centre, Hamburg, Germany

4 Space Medicine Team (HRE-OM), ISS Operations and Astronauts Group, European Astronaut Centre, Directorate of Human Spaceflight and Robotic Exploration, European Space Agency, Cologne, Germany

**Corresponding Author**

Isabella Wiedmann

Linder Höhe

51147 Cologne

Email: [isabella.wiedmann@esa.int](mailto:isabella.wiedmann@esa.int)

Supplement 1 – Introduction Email

Dear {FIRSTNAME},

In the spirit of diversity and inclusion Dave Parker, Director of Human and Robotic Exploration of the European Space Agency announced in March 2021 that the 2021 ESA astronaut selection will be the first astronaut selection in the history of human space flight to include astronaut candidates with physical impairments.

As part of the recruitment and training preparations for our future crew, including astronaut candidates with physical impairments, ESA’s Space Medicine Team (ESA HRE-OM) under the leadership of Dr Guillaume Weerts is currently investigating different options to optimise astronaut recruitment procedures and readiness assessments to assure crew safety and mission success. As part of the parastronaut feasibility project ([ESA - Parastronaut feasibility project](https://www.esa.int/About_Us/Careers_at_ESA/ESA_Astronaut_Selection/Parastronaut_feasibility_project)), it is ESA’s primary goal to work with our expanded pool of potential (para-) astronaut candidates and operational experts to mitigate risks and to create an operational environment that allows astronauts with physical impairments to become fully proficient and competent crewmembers.

As part of this internal ESA HRE-OM investigation we have identified you as an expert and we would very much appreciate if you could invest a little bit of your time and knowledge to help us prepare for this new and exciting endeavour. As a subject matter expert, we would kindly ask you to take part in our so-called Delphi-survey ([Delphi Survey | involve.org.uk](https://www.involve.org.uk/resources/methods/delphi-survey)) which consists of 2-3 survey rounds with open and closed questions.

For each survey round, you will be able to access and modify your survey for **seven days**. After seven days, all results from all experts will be collected, clustered, and re-sent to you to provide your feedback on the collated results. The expected time to complete a questionnaire in each round is approximately ten minutes. Naturally, all input provided by you as well as your identity are always fully anonymised - your survey input cannot be attributed to your person, neither by the other experts, nor by the investigators of this study.

The first round is entitled:

"{SURVEYNAME}"

"{SURVEYDESCRIPTION}"

Please follow this link below to access our safe ESA portal that will allow you to take part in our survey.

We thank you very much in advance for supporting this extremely exciting project.

Sincerely yours,

----------------------------------------------
Click here to do the survey:
{SURVEYURL}

If you do not want to participate in this survey and don't want to receive any more invitations please click the following link:
{OPTOUTURL}

If you are blacklisted but want to participate in this survey and want to receive invitations please click the following link:
{OPTINURL}

Supplement 2 – Questionnaire Round 1


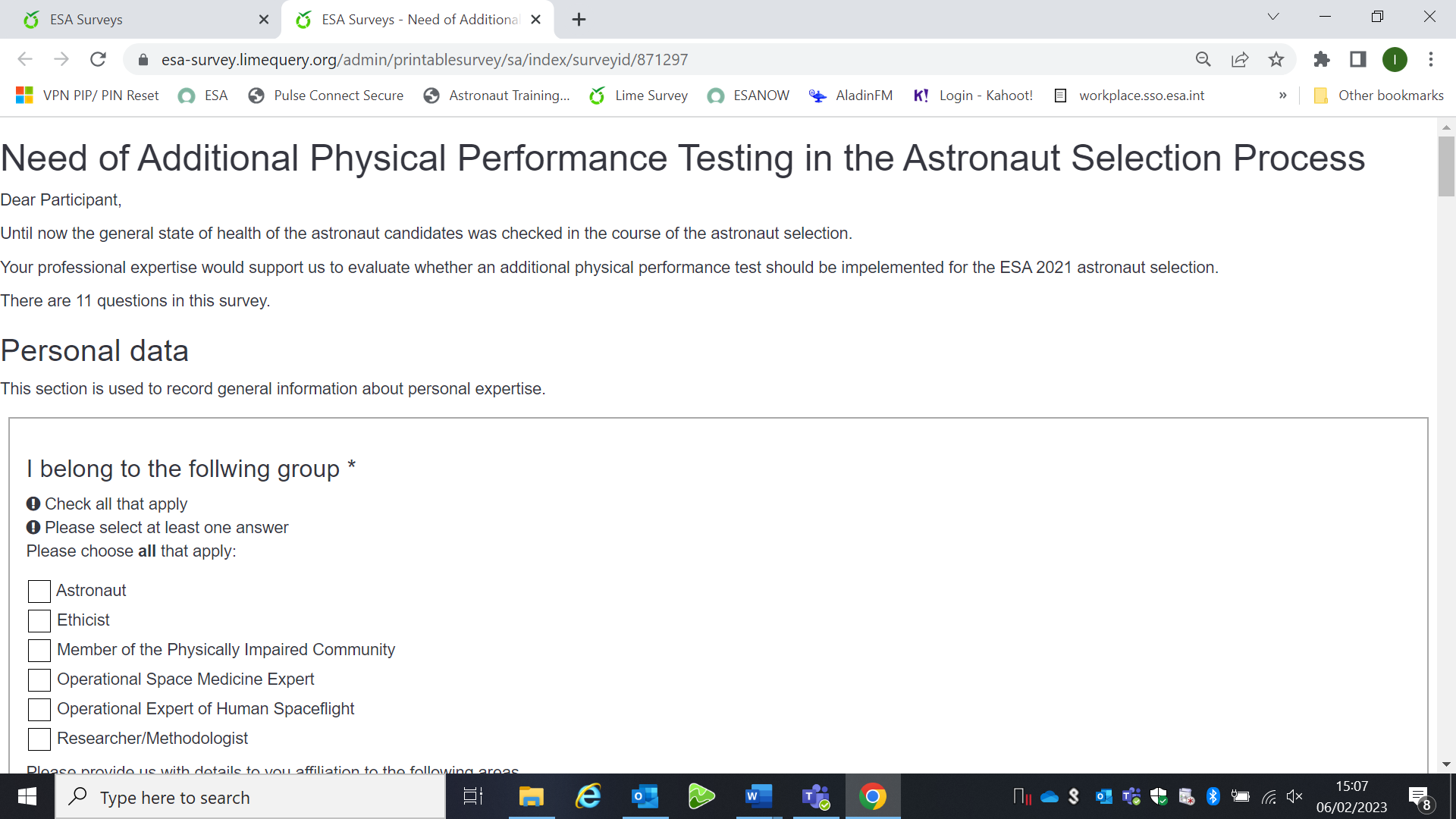


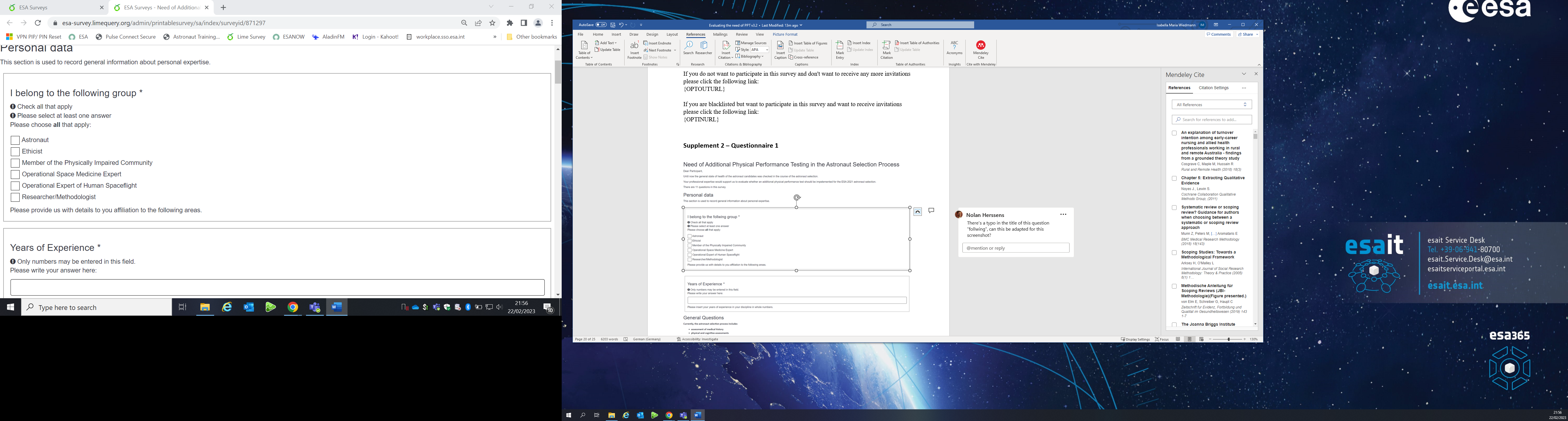


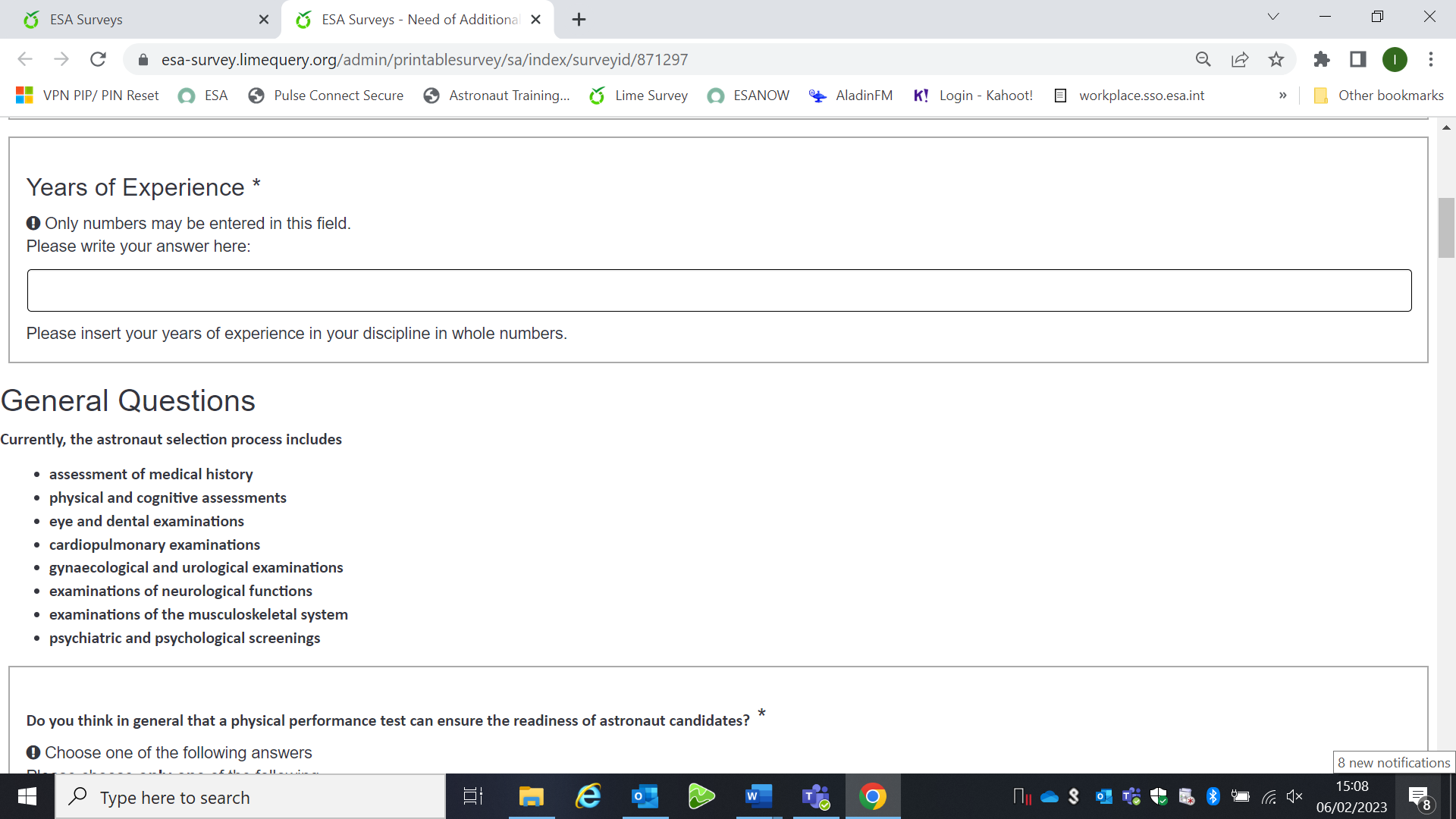


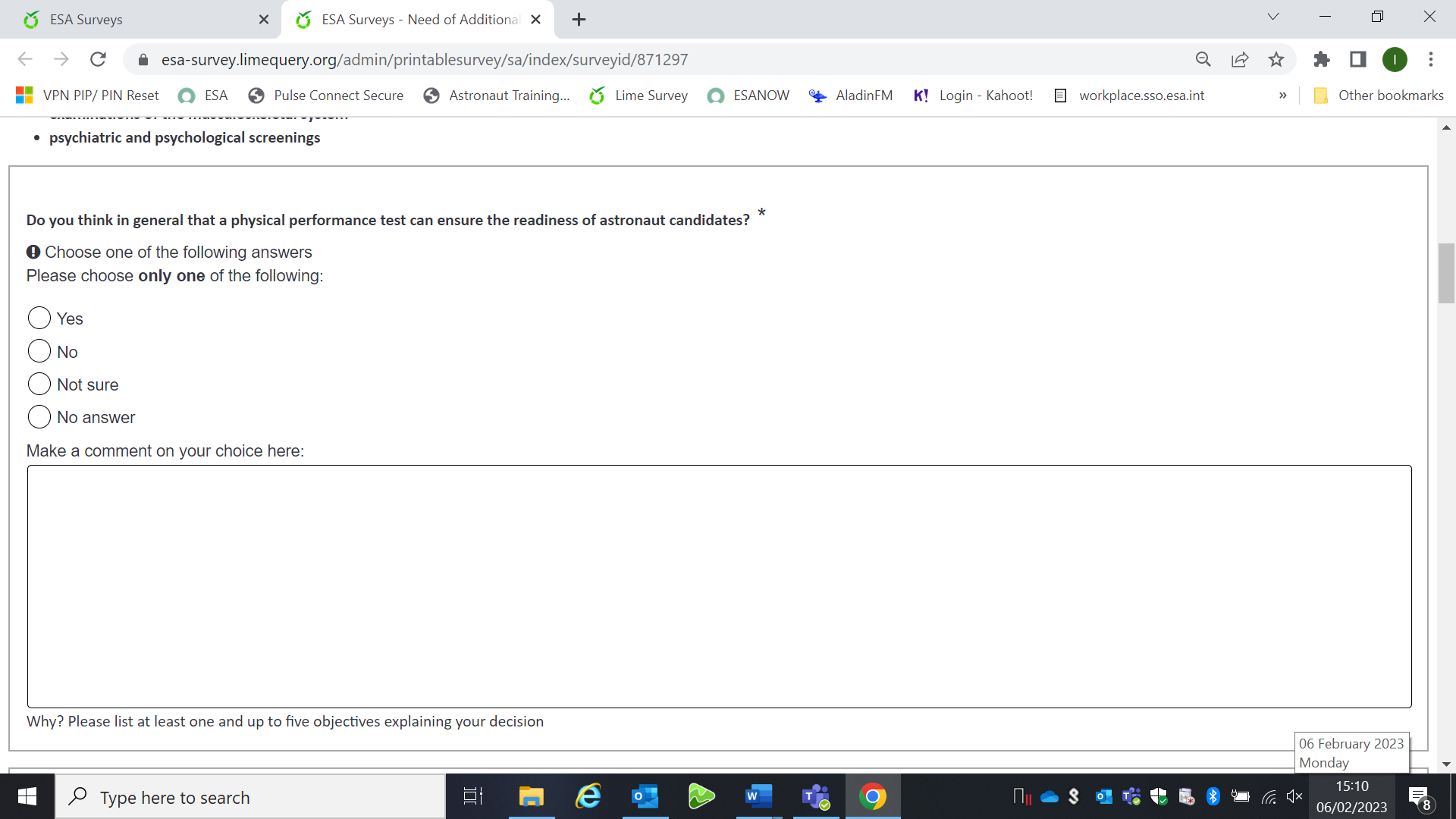


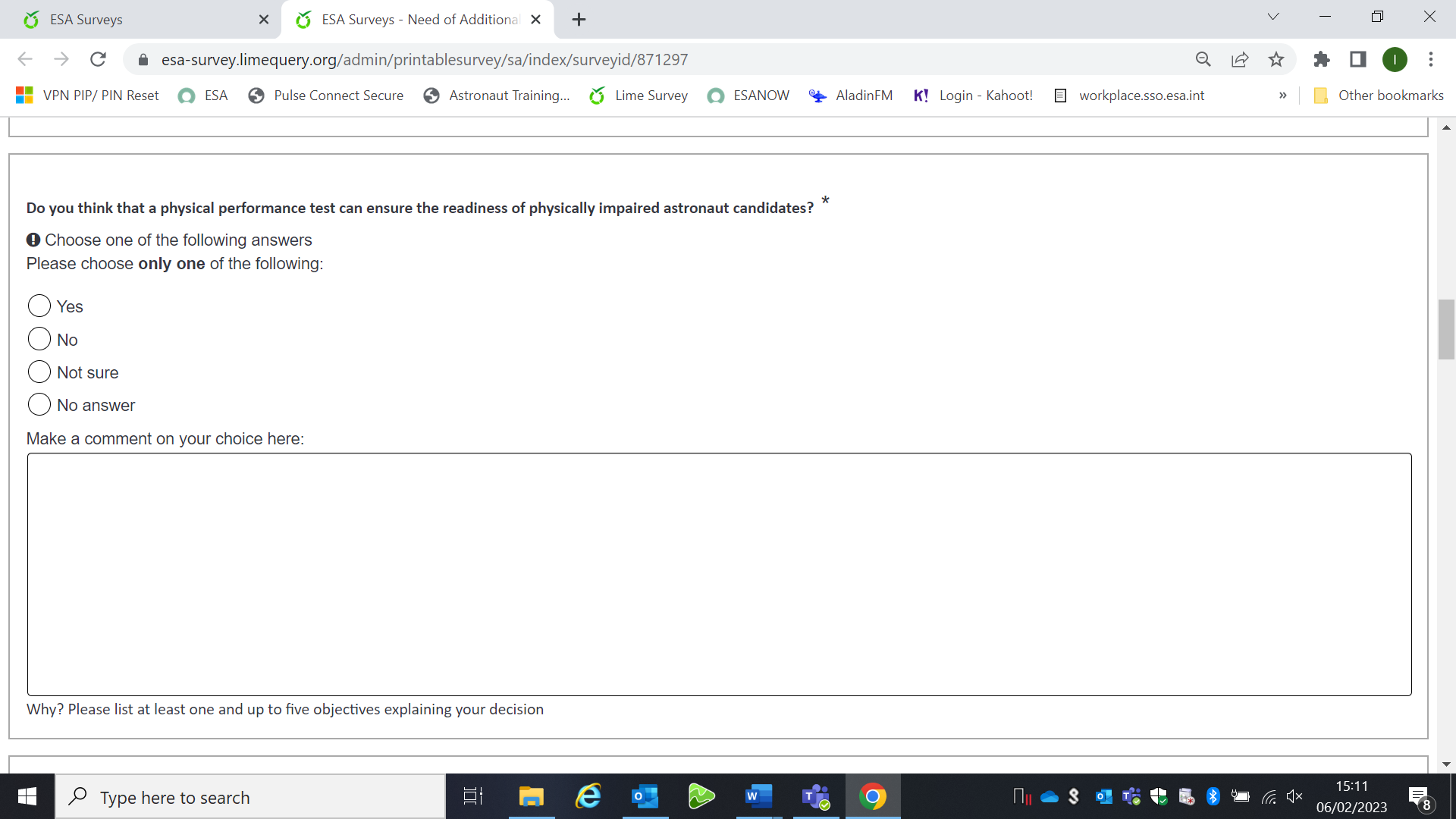


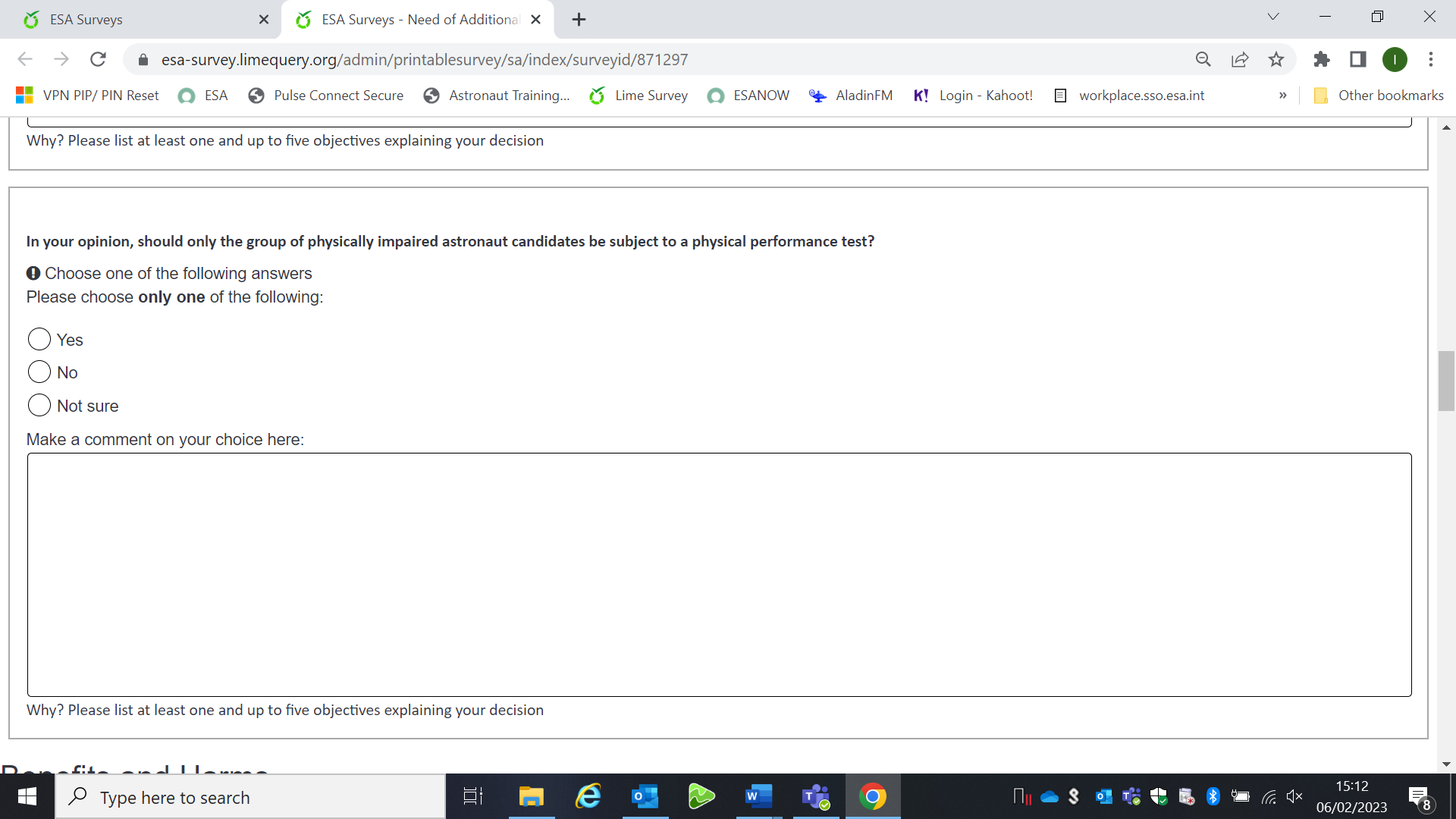


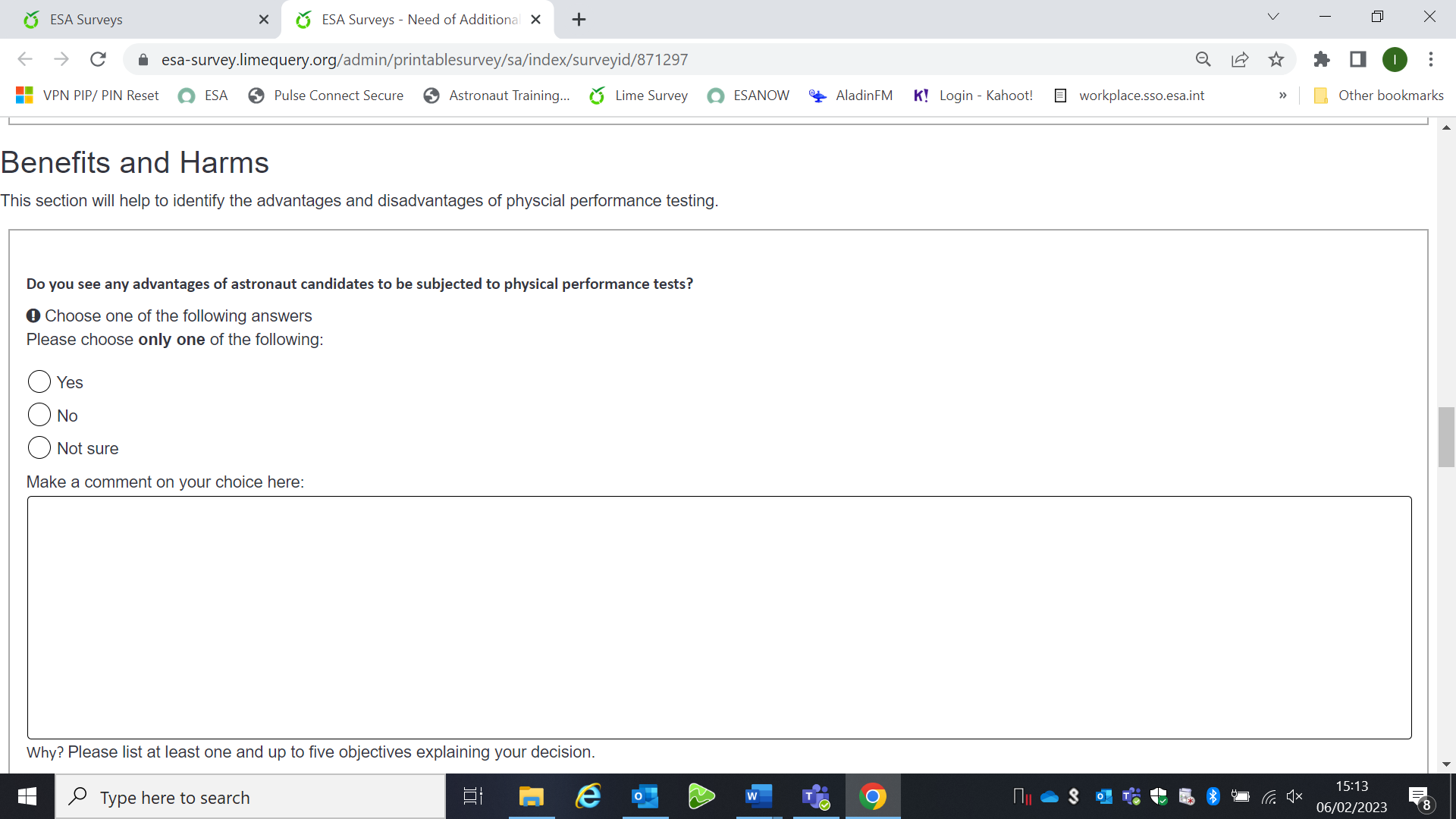


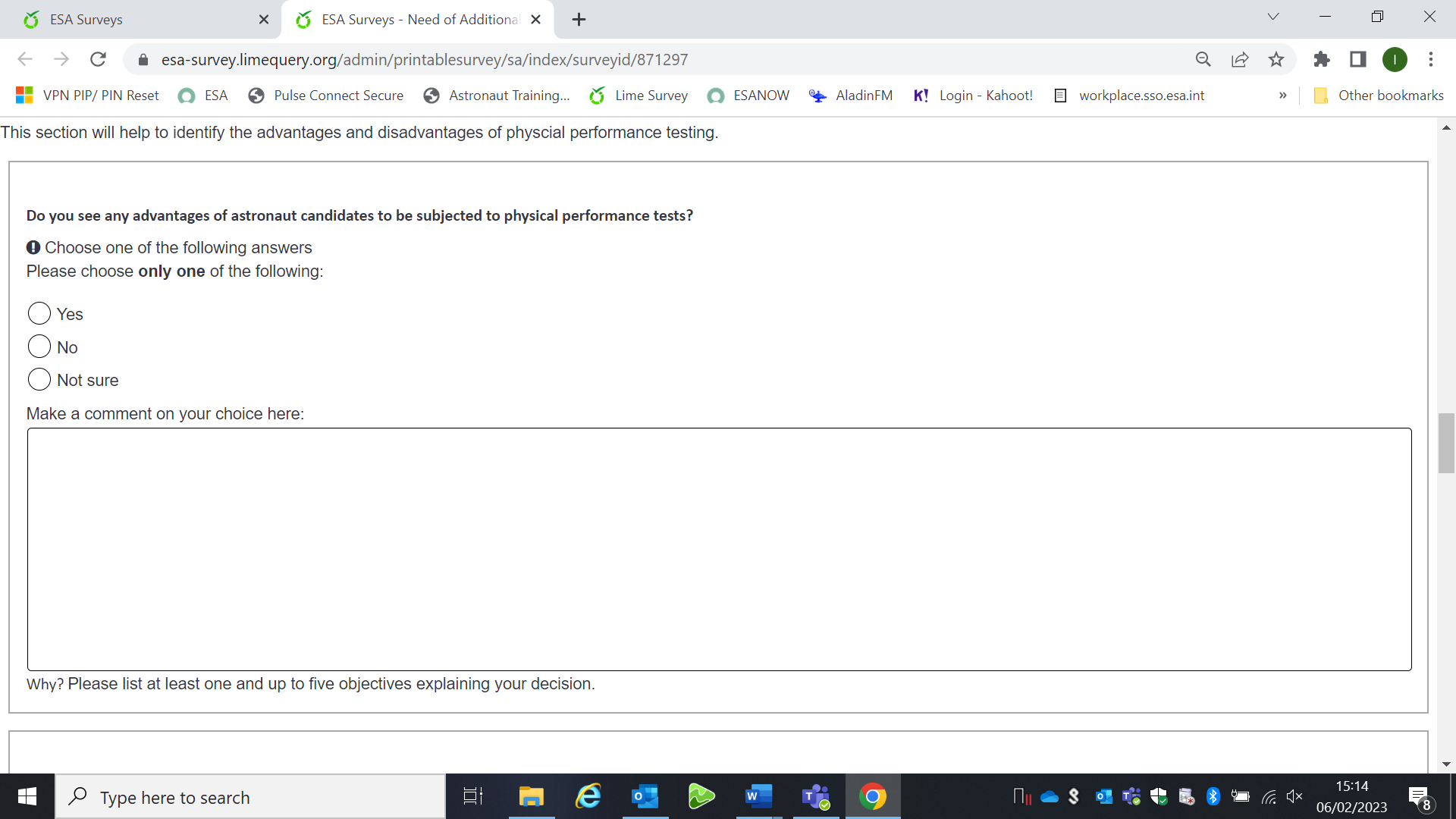


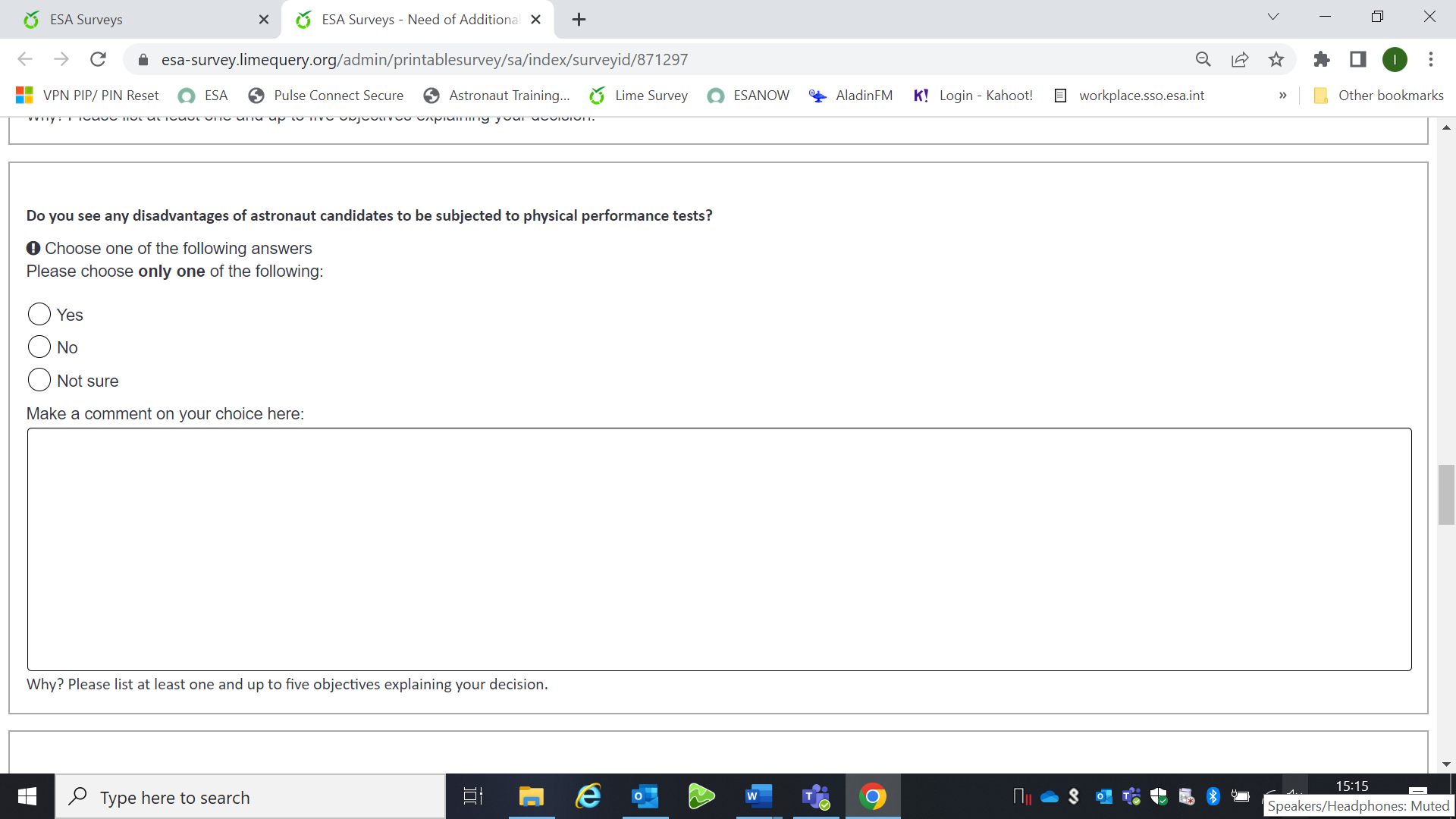


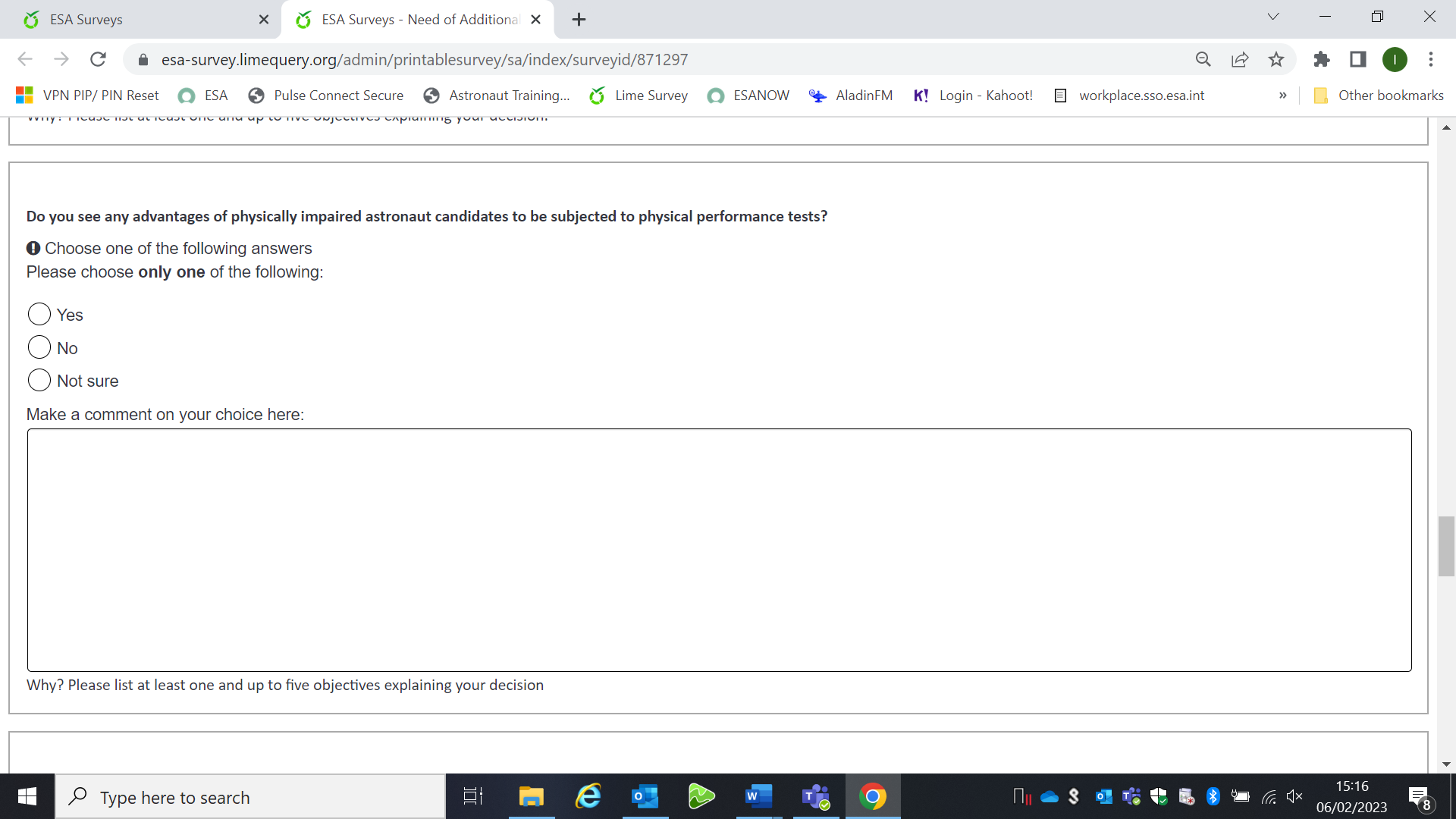


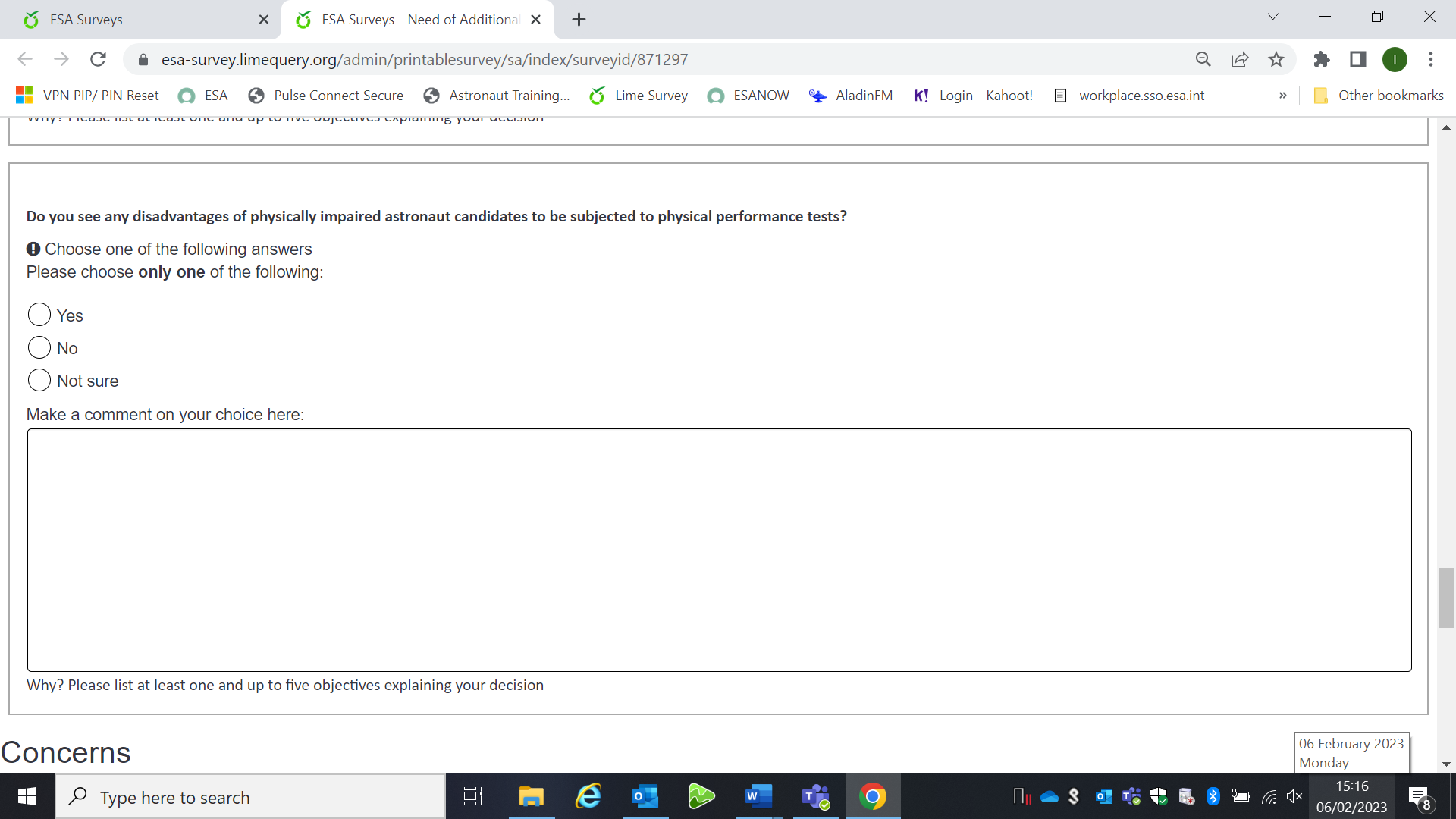


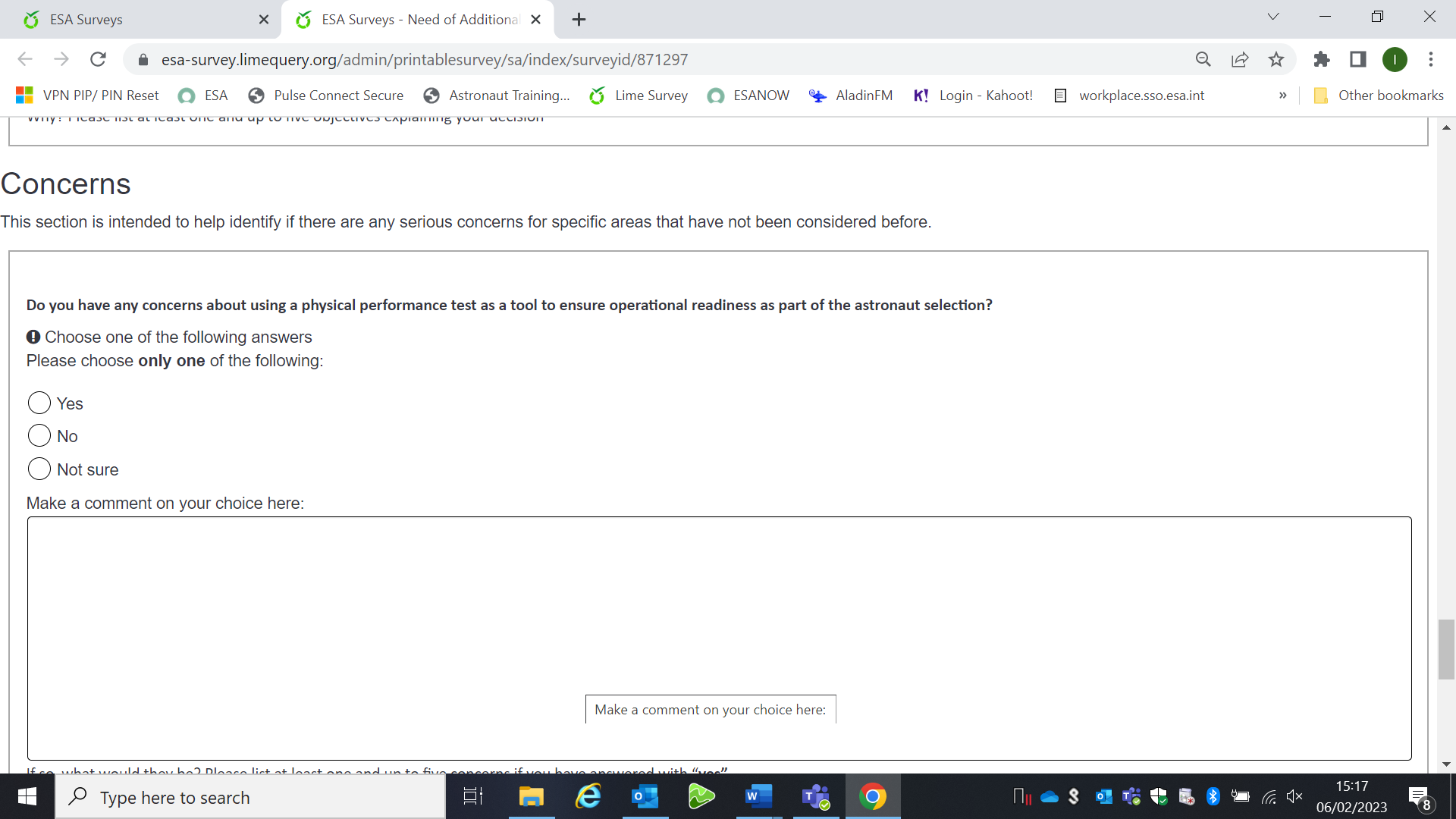


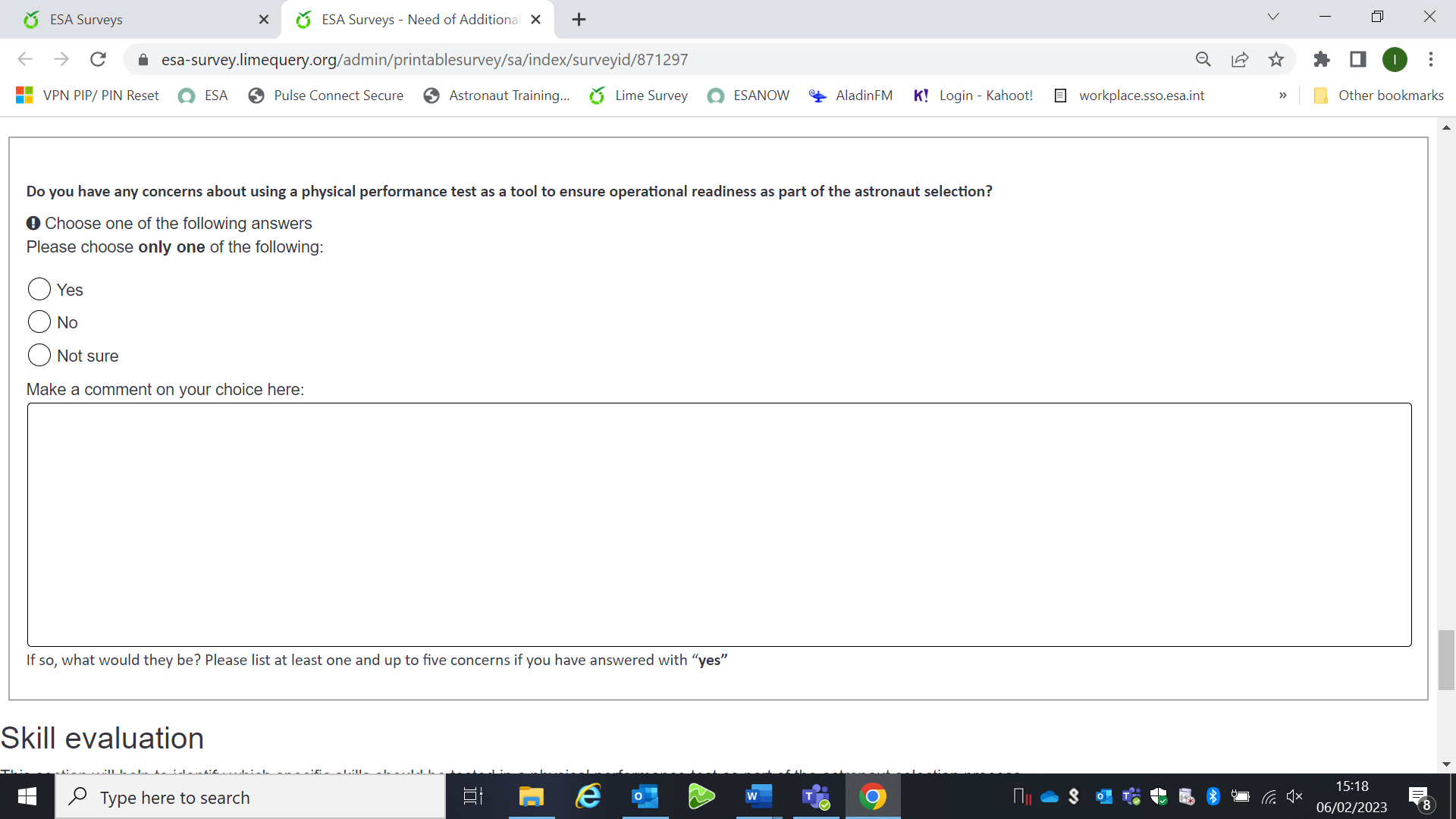


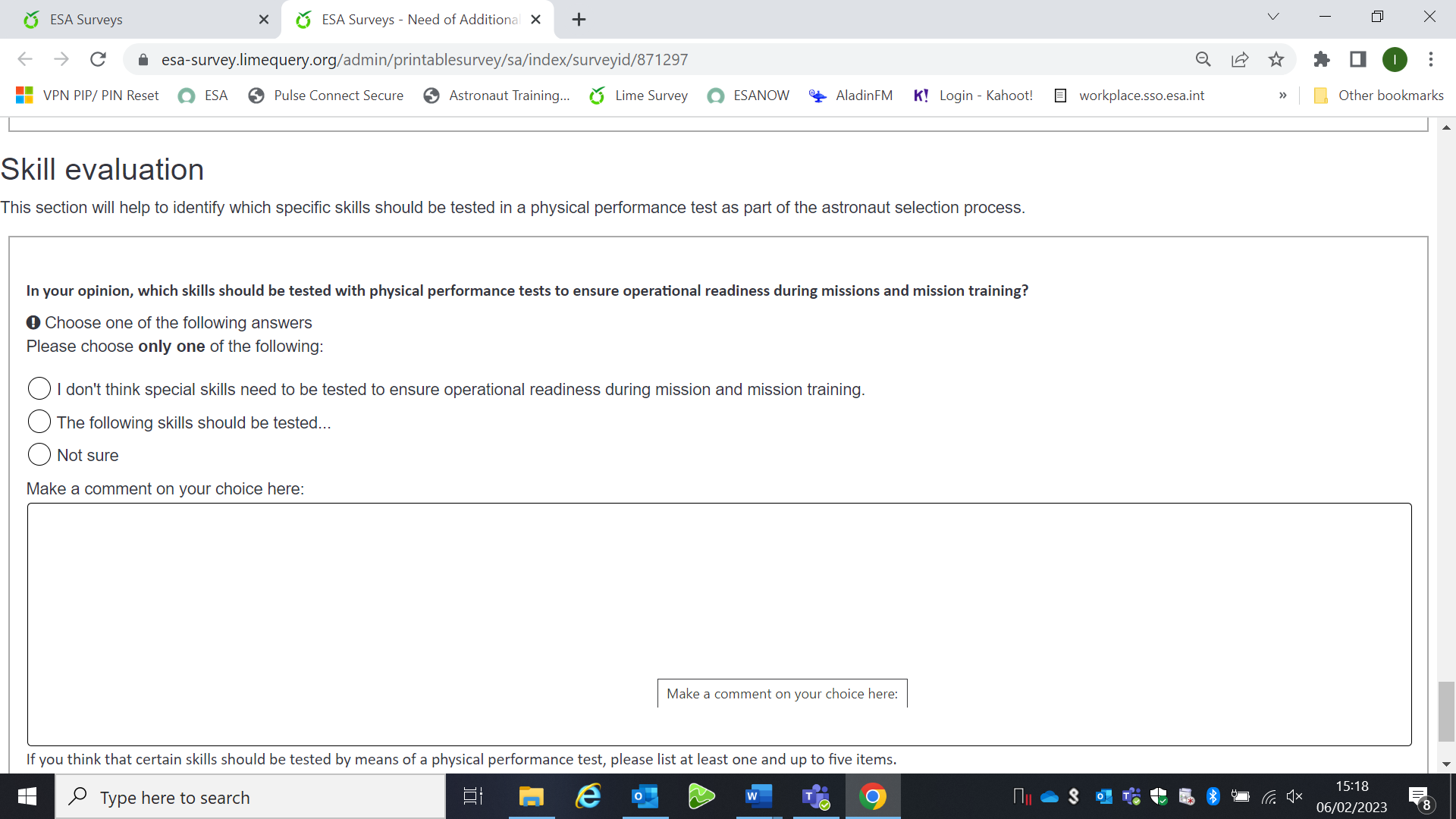


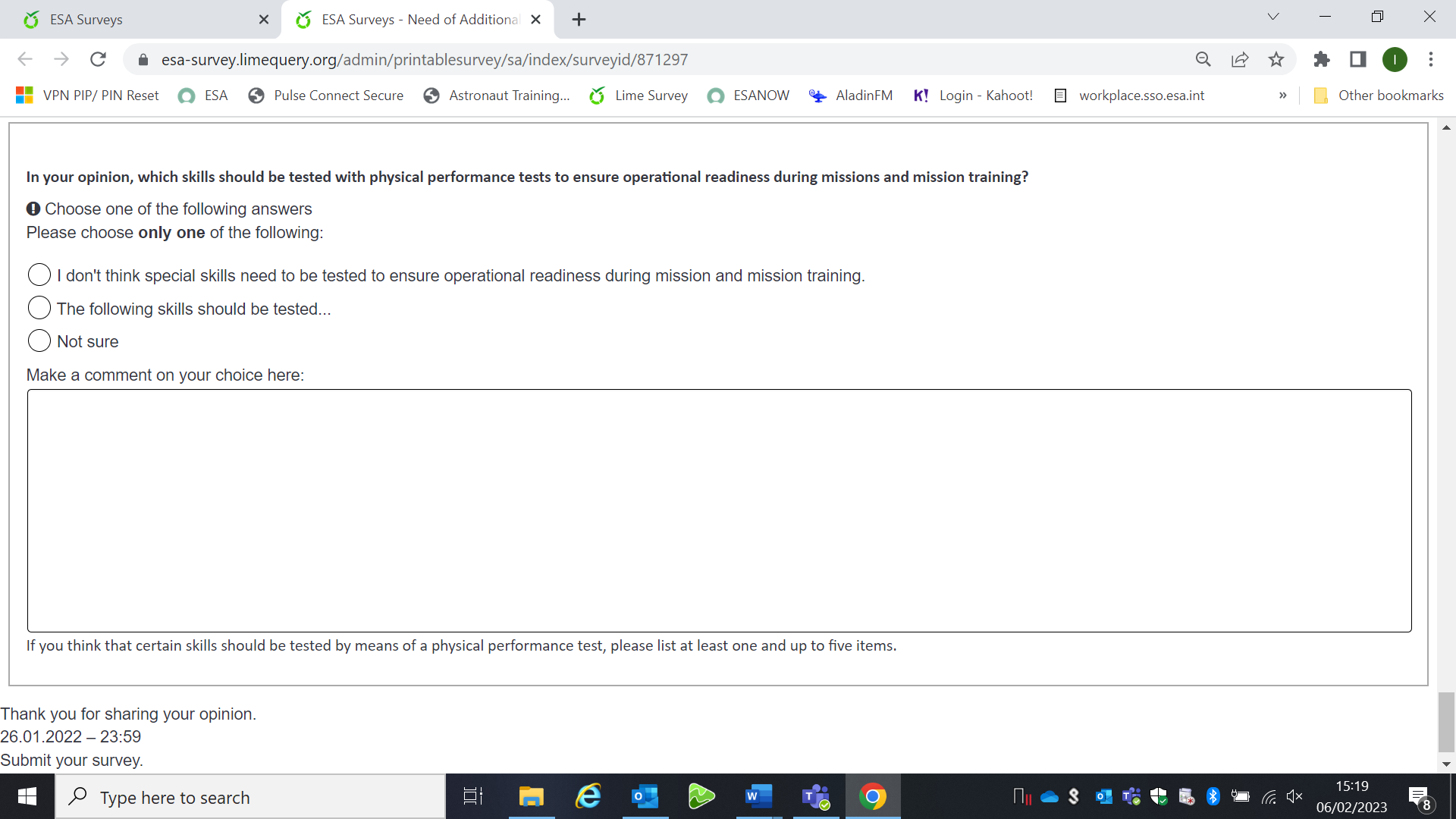


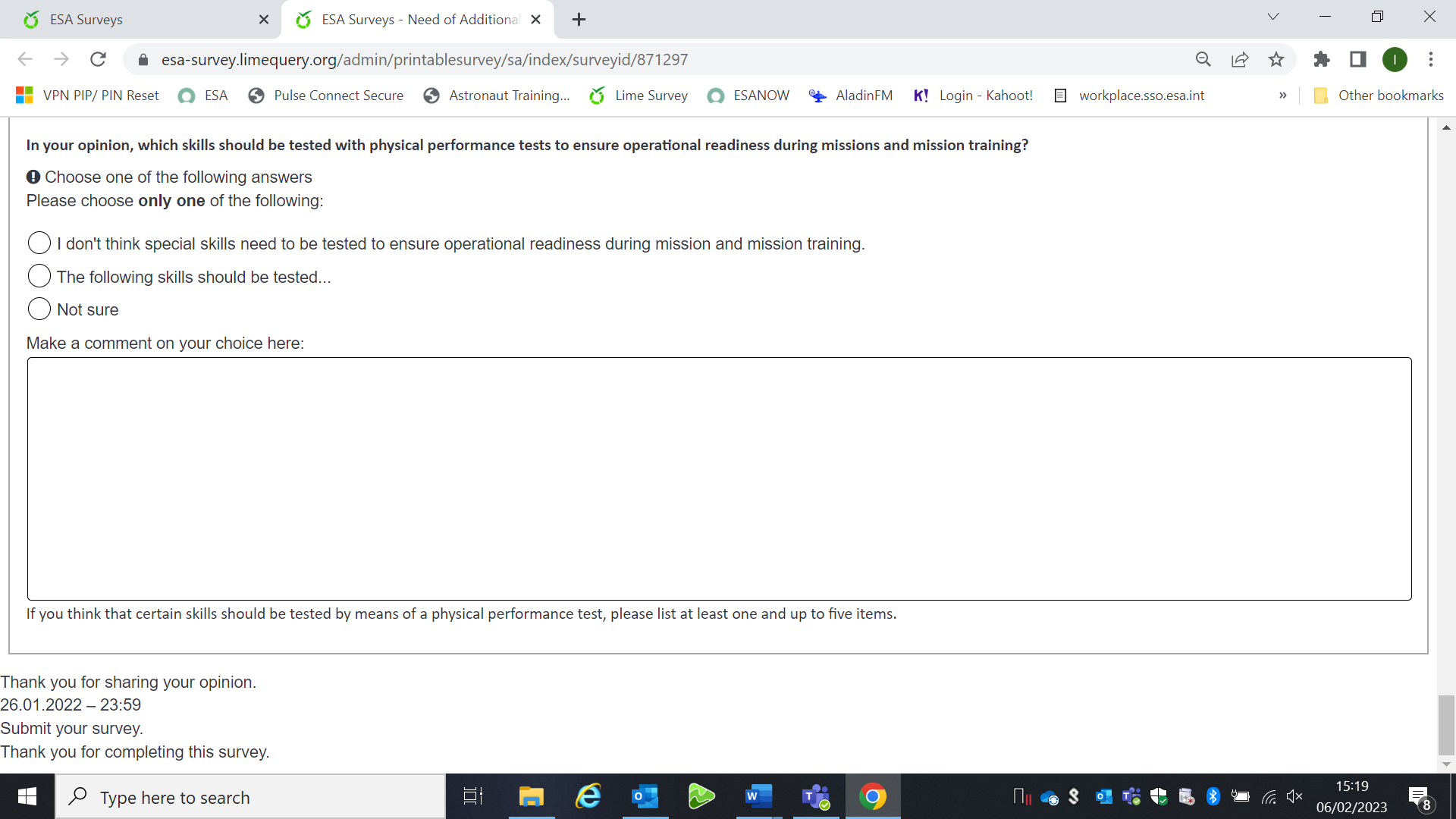


Supplement 3 – Results Round 1

Table 1: Characteristics of the expert panel

|  |  | **I belong to the following group** | | | | | |  |
| --- | --- | --- | --- | --- | --- | --- | --- | --- |
| **ID** | **Status** | **Astronaut** | **Ethicist** | **MoPIC**  **(Member of the Physically Impaired Community** | **OSME**  **(Operational Space Medicine Experts)** | **OEHS**  **(Operational Experts for Human Spaceflight)** | **Researcher** | **Years of Experience** |
| 1 | incomplete |  |  | Y |  |  | Y | 30 |
| 2 | complete | Y |  |  |  |  |  | 12 |
| 3 | complete |  |  |  | Y | Y |  | 17 |
| 4 | complete |  |  | Y |  |  |  | 12 |
| 5 | complete |  |  |  | Y |  | Y | 8 |
| 6 | complete |  |  |  |  |  | Y | 12 |
| 7 | complete |  | Y |  |  |  |  | 40 |
| 8 | complete |  |  |  | Y |  |  | 30 |
| 9 | complete | Y |  |  |  |  |  | 20 |
| 10 | complete |  |  |  | Y |  |  | 40 |
| 11 | complete | Y |  |  |  |  |  | 30 |
| 12 | incomplete |  |  |  | Y | Y |  | 6 |
| 13 | complete | Y |  |  |  |  |  | 12 |
| 14 | complete |  |  |  | Y |  |  | 9 |
| 15 | complete |  |  | Y |  |  | Y | 11 |
| 16 | complete |  |  | Y |  |  |  | 10 |
| 17 | complete |  |  | Y |  |  | Y | 30 |
| 18 | incomplete |  |  |  |  |  |  |  |
| 19 | complete |  |  | Y |  |  |  | 11 |
| 20 | complete |  |  |  | Y | Y |  | 6 |
| 21 | complete |  | Y |  |  |  |  | 14 |
| 22 | complete | Y |  |  |  | Y |  | 33 |
| 23 | complete | Y |  |  |  | Y |  | 31 |
| 24 | complete |  |  |  | Y |  | Y | 15 |
| 25 | complete |  |  |  | Y |  |  | 15 |
| 26 | incomplete |  |  |  | Y |  |  | 8 |
| 27 | complete |  |  |  | Y | Y |  | 10 |
| 28 | incomplete |  |  |  | Y |  |  | 8 |
| 29 | complete |  |  |  |  | Y |  | 14 |
| 30 | complete |  |  |  |  | Y |  | 22 |
| 31 | complete | Y |  |  |  | Y |  | 31 |
| 32 | complete |  |  |  | Y |  |  | 17 |
| 33 | complete |  |  |  | Y | Y |  | 5 |
| 34 | incomplete |  |  |  |  |  |  |  |
| 35 | complete |  |  |  |  | Y |  | 32 |
| 36 | incomplete |  |  |  |  |  |  |  |
| 37 | complete |  |  |  | Y | Y |  | 13 |
| 38 | complete |  |  |  |  | Y |  | 20 |
| 39 | complete |  |  |  |  |  | Y | 29 |
| 40 | complete |  |  |  | Y |  |  | 8 |
| 41 | complete |  |  | Y |  |  |  | 12 |
| 42 | complete |  |  |  | Y | Y |  | 5 |
| 43 | complete |  |  | Y |  |  |  | 12 |
| 44 | complete |  |  |  |  |  | Y | 23 |
| 45 | incomplete |  |  |  |  |  |  |  |
| 46 | complete |  |  |  |  |  | Y | 36 |

Table 2: Advantages of a Physical Performance Test

|  | **QA1** |  | **QA2** |  | **QA3** |  |
| --- | --- | --- | --- | --- | --- | --- |
| **ID** | **Do you think in general that a physical performance test can ensure the readiness of astronaut candidates?** | **Comment** | **Do you think that a physical performance test can ensure the readiness of physically impaired astronaut candidates?** | **Comment** | **In your opinion, should only the group of physically impaired astronaut candidates be subject to a physical performance test?** | **Comment** |
| 1 |  |  |  |  |  |  |
| 2 | Y | Whether on a short or long duration flight, astronauts need to perform high level physical exercise in order to stay healthy. To perform in space, they need to be able to perform at high level on the ground. Thus, verifying their capabilities to perform is a requirement. | Y | If we select physically impaired astronauts to be fully fledged members of a crew, they need to be able to demonstrate the same capabilities. | Y | see above comment. |
| 3 | N | It is already a good point, but it is definitively not enough. First because the physical performance reflects what happen at that time when it is checked but in the next future it might change, " you might be fit Today and not Tomorrow". And of course, beside the physical performance is may be most important would be the second point " the psychology" which for astronaut is really important. How are you able to deal with uncertainties and working in a team with others. | NS | The answer is different from the previous one, in that case we have a physically impaired astronaut, so the physical performance will be connected to the physical problem. If you have a scale and decide of the minimum level of physical, you need to decide what is the level the candidate should reach to be accepted. But for sure if he/she has some physical impairments it depends on the impairment you considerer as a prerequisite minimum to apply the job | N | Same should apply to all, only the score level might change |
| 4 | Y |  | Y | along with strict medical examination | N |  |
| 5 | Y | In general, cardiovascular endurance as measured by achieved number of METs (metabolic equivalent) on max/submax stress test correlates with vascular risk level. Endurance would also be of importance in various scenarios, e.g., emergency landing in remote and hostile environment. Furthermore, the level of fitness indicates whether the candidate is used to do regular exercise - which is a crucial part of the daily routine on the ISS. | Y | Same as above, and perhaps even more as the candidate may need to compensate. | N | All candidates should demonstrate a good level of fitness. It is not necessary to be an athlete, but the cardiovascular endurance level should preferably be above average after correction for age and gender. It is often preferable to test the endurance on a treadmill to avoid biases caused by technical experience in other activities. However, this may be challenging for some of the para-astronaut candidates. Rowing ergometer is also considered to be an excellent test for measuring cardiovascular endurance. |
| 6 | Y |  | Y |  | N |  |
| 7 | Y | To eliminate significant risks to the individual's health | Y | As above, to eliminate individual risks | N | Physically impaired astronaut candidates should not suffer discrimination - I imagine that some physical impairments night be advantageous to the role |
| 8 | Y | Emergency response to launch and landing contingencies leading to a space vehicle evacuation, as well as completion of extravehicular activities on orbit are examples of tasks requiring optimal physical performance to be tested pre-flight and in flight. A safe return to active duty and flight status, after a mission, will also require a physical performance test. | Y | Physical performance is a significant requirement to ensure a safe evacuation of a vehicle in an emergency scenario for all crew members, irrespective of any physical impairment. In case the rescue forces should not be at landing site and the other crew members are incapacitated, a physically impaired crew member will need to independently and safely evacuate the vehicle, seek shelter and, if at all possible, help the other crew members to safety. Performance testing during flight and prior to landing will provide the physically impaired astronaut and the other crew mates with the insight on the level of fitness to fulfil these tasks. | N | In an emergency scenario, adequate physical performance is a requirement for all crew members to ensure a safe evacuation of a vehicle, irrespective of any physical impairment. The same applies to any crew participating in any demanding physical activity, e.g., extravehicular. |
| 9 | NS | Physical fitness can be trained as long as the person is in general good health. We don't need sportsmen to fly to space. | NS | What is required is to check if the candidate is able to perform certain functional tests, not so much physical performance | NS | They should all undergo a functional test to make sure they can perform the functions that are required by an astronaut, especially during emergency ingress and egress |
| 10 | Y | Physical fitness is a requirement for being an astronaut | Y | Fitness is a countermeasure to prevent too much effects of weightlessness | Y | But should be adapted to her/his disability |
| 11 | N | A physical performance test is certainly needed but is obviously not the only criterion to ensure that a candidate is ready to become an astronaut. | N | The previous answer would apply to physically impaired candidates too of course, but the physical criteria for impaired astronaut selection must take into account the design of the space facilities which might not be adapted to certain impairments. | N | From my experience, physical performance tests have always been part of astronaut selection processes in all countries. |
| 12 |  |  |  |  |  |  |
| 13 | Y | Up to a certain point. I don't think that the test should require a high-level performance. But a basic level of fitness will be required for an astronaut (no difference between traditional and parastronauts). Some have argued in the past that such a test is not necessary, because fitness can be built up and trained at any time. Thus, it was argued that even candidates with a substandard level of fitness can be selected, and be trained up later on. However, I do not agree with that. I think that in addition to learning about the current state of fitness of an applicant, such a fitness test would also be an indicator of lifestyle of an applicant. If they did not maintain a basic level of fitness at the time of their application, it is also more likely that they will stop maintaining that level of fitness at a later stage. | Y | same as above | N | there should be NO difference between the two groups! |
| 14 | N | In general, we want to ensure that astronaut candidates are healthy people with no (or minimal) underlying medical issues. However, this can most probably be checked via all the listed medical examinations. A pure physical performance test (focused more on endurance and strength) might be exaggerated, as we are not looking for top athletes and the job of astronaut also does not require a more than average physical fitness (with the exception of performing EVAs). A physical test more focused on hand-eye coordination and motoric skills for the hands is valuable for the astronaut selection. | N | Like for the astronaut candidates, for the physically impaired astronaut candidates we are also not looking for the candidates with the best endurance or the most strength. As long as they are healthy candidates, with good hand/arm-motoric skills, a physical performance test will not bring much. | N |  |
| 15 | Y | Operating in a demanding environment over an extended period of time requires abnormal levels of physical preparedness. The space environment is no different, and the risks inherent in operating there are high. As many or most of the physical demands are known, astronaut candidates should be prepared and assessed against these to ensure the minimisation of risk associated with the physicality required to operate in space. | Y | As with astronaut candidates who are not physically impaired, the physical preparedness of astronaut candidates with an impairment can and should be assessed through the evaluation of performance against a pre-defined model of the demands of operating in space. This performance evaluation should be task or outcome based, objective by nature, and encompass any definitive assistive technology developed and approved for the (likely) mission. | N | Any person operating in an extreme environment should be assessed for physical preparedness in order to minimise mission risk. |
| 16 | Y | Thorough testing and examination are done in most professional industries where candidates need to perform. | Y | Performance testing can ensure the right person with disability can deploy. Not every disability may be suitable for space flight. | N | Even though your aim is to select astronauts with physical disabilities, they still need to be able to survive and operate in space. They should be assessed to the same level as normal astronauts in all aspects then their disability assessed on an individual basis. |
| 17 | Y | There will be a known dataset, and also lived experience, of former astronauts which can confirm or refute the value of these tests to assess the ability to perform the tasks required while in space. | N | It will ultimately depend upon the nature of the test but will also require more detailed assessment of task performance required while in space. It is possible that for some physically impaired astronaut candidates that the tasks will be easier in a weightless environment that will not be assessed by standard physical assessments. For example, I have an incomplete spinal cord injury and can move much more freely in water than on land. So, the tests will need to be task specific to the environment in which they are to be performed. | N | It is logical to make a physical assessment of all candidates but as described above the physical performance tests need to be task specific to the environment. |
| 18 |  |  |  |  |  |  |
| 19 | Y | Space is a tough environment. You must be physically capable. | Y | Physical impairment does not prevent an individual from being physically capable if the test is accessible. | N | All should be held to the same standard. |
| 20 | Y | I believe that a physical performance test can evaluate adequately the readiness of an astronaut candidate because it provides a general idea of the person's fitness and related aspects (e.g., range of motion). If the fitness level is already mission-sufficient, then all is well. If it shortly under the needed level, then with suggested training, the candidate can achieve what is needed. Thus, I believe some flexibility in interpreting the results is necessary instead of introducing 'hard lines'. If the fitness level is significantly below what is needed, then we know the candidate will most likely not achieve readiness in time for a mission. | NS | I believe that depends on the nature of the physical performance test: a VO2max treadmill test will naturally have to be adapted for a person whose left leg may be shorter than the right leg, as an example. Given that the physical performance test will be adapted to the impairment of the candidate, then yes, i believe that the physical performance test can ensure the readiness of that candidate. | N | Personally, I would advocate for all candidates to pass such a test. First of all, when recruiting for analogues and also for spaceflight, we know that no human candidate can ever be 100% perfect in health and anthropometry. We know also that even in a "fully healthy" candidate who passed the tests, some minor medical/physical findings may be missed during examination. Having all candidates (impaired and not impaired) pass the same physical test ensures that even the not impaired ones fully comply with the same requirement and i may also add ethically to the uniformity of the selection. |
| 21 | N | Of course, physical performance is an important characteristic for astronauts, but reducing readiness to that would risk relegating other important characteristics to the background. The readiness of an astronaut should therefore be assessed on the basis of several characteristics and weighted according to the requirement profile depending on the role/function. However, it will probably not be possible to avoid setting minimum values for achieving (part of) a test; these should express that this test result is required in order not to put oneself and others in danger in e.g., emergency situations. This is also an ethical requirement in dealing with risks. Thresholds for test results should, as far as possible, be evidence-based or at least experience-based to be objective as far as possible and not systematically biased (prejudices, misconceptions, perhaps even &amp;quot;ideologies&amp;quot; regarding what an &amp;quot;astronaut should be&amp;quot;). | N | Physical performance tests should not be the only characteristic, even for impaired astronaut candidates. But the difficult question is whether to use the same tests, or adapted tests that take into account the disability in question. It must also be considered whether (medical) technical aids that can partially compensate for the disability should be taken into account (or whether one test with and one test without should be performed). Furthermore, a threshold value will again be required, which is assumed to be reached in order to be able to avert dangers to oneself and others (as far as realistic). Using the same test may be fair to all candidates (impaired and not impaired) but may be unfair to impaired candidates who may have other skills that are valuable. Nevertheless, fairness alone cannot be decisive in view of the mentioned duty to be physically able to meet the minimum requirements in order to minimize risks (in general and especially in emergencies). Especially in the case of tests for impaired candidates, it seems particularly important that they are as evidence-based as possible and thus do not make excessive demands, but rather test what is actually required. A test that requires something that an impaired candidate can only achieve with difficulty, but which is also not absolutely necessary (to achieve this with this performance), but rather is based on prejudices or (not always realistic) ideal conceptions of an astronaut, would be unfair and could be discriminatory without justification. | N | Physical performance is likely to be important in general, so it would be difficult to see why only impaired candidates should take these tests. At most, the question could arise whether there are certain physical performances (e.g., also movement possibilities) that a non-impaired candidate can in all probability perform, but an impaired candidate cannot with certainty. Then it would be understandable if this one performance would be tested (and would be rather almost chicanery to let the non-impaired candidate do the test if it would be clear that he will succeed anyway). Again, however, this should only be done when there are set thresholds to be met (see above), and not as a matter of principle, as that would be an unjustifiable form of discrimination to require a test of one group but not the other, even though there is no good reason for that test. |
| 22 | Y | need to verify good psychomotricity for operating complex machines. pure physical performance can be limited to a stress (cardio) test | Y | same as for non-impaired candidates (essentially cardio and hand motor skills) | N | No, if the aim of the physical performance test is only cardio, because it applies to all |
| 23 | Y | Besides the annual medical maximum exertion test, a set of monitored mild physical exercises were part of the early Russian spaceflight training. IN addition to the classical track and field disciplines we did 'exams' in trampoline and swimming. A similar mild ingoing exam (e.g., the German Sportabzeichen, which is staggered by age) could be part of the astronaut selection just to see whether the candidates have the psychological will to invest also physical strength to the point of some exertion. | Y | Yes, but it should apply the same staggered scale like the non-impaired astronauts, which can be done by choosing the adequate disciplines and allow protheses, helps etc. In no way should the impaired condition lead to a feeling of inferiority towards the non-impaired candidates. Candidates applying for the Parastronaut Programme as an achiever type subgroup of impaired persons will be more than proud and willing to show in how far they cope with their impaired condition. They would most probably expect such a test, because this is where they are good at and stand out from the impaired 'crowd'. | N | Yes, but it should apply the same staggered scale like the non-impaired astronauts, which can be done by choosing the adequate disciplines and allow protheses, helps etc. In no way should the impaired condition lead to a feeling of inferiority towards the non-impaired candidates. Candidates applying for the Parastronaut Programme as an achiever type subgroup of impaired persons will be more than proud and willing to show in how far they cope with their impaired condition. They would most probably expect such a test, because this is where they are good at and stand out from the impaired 'crowd'. |
| 24 | Y |  | Y |  | N |  |
| 25 | Y | Yes, if the physical performance test and the minimum thresholds are based on the occupational requirements of the job (i.e., the astronauts' essential tasks and the metrics that determine an acceptable completion of the task), then they could be used to ensure the readiness of astronaut candidates. | Y | Yes, if the physical performance test and the minimum thresholds are based on the occupational requirements of the job (i.e., the astronauts' essential tasks and the metrics that determine an acceptable completion of the task), then they could be used to ensure the readiness of astronaut candidates. As long as the candidates can successfully and safely complete the essential tasks, a physical impairment should not impact their readiness. | N | All candidates should undergo the same physical performance test. |
| 26 |  |  |  |  |  |  |
| 27 | Y | The physical performance tests/battery need to be comparable with scientific results (e.g., standard VO2max test gives an objective result of aerobic performance and is comparable within age and gender groups) and also should be task oriented (e.g., CMS training on ISS include squatting, so test needs to perform squats) | Y | But depends on the level of physical impairedness. I think we can operate for now only in a small range of what is acceptable. Including a wider range of physical impairedness can follow later. | N | Equal treatment for all! |
| 28 |  |  |  |  |  |  |
| 29 | Y | physical health and fitness are basic requirements for astronauts to withstand launch and microgravity phases. It enables them to work onboard and conduct their mission. | Y | Also, the impaired astronaut needs to be fit and healthy to withstand launch and microgravity while being able to work onboard ISS. | N | Because physical health and fitness is a basic requirement for ANY astronaut |
| 30 | NS | physical performance can be trained in any person, so is not a fixed character physical performance is more important for long duration missions than for short missions’ physical strength is less required on-orbit (with exception of special cases, e.g., EVA) | NS | for a physically impaired candidate, at least a completely different test would be applicable in my mind - testing the reach / movability etc physical performance can be trained in any person, so is not a fixed character | N | It would be rather awkward to put an additional test on the physically impaired candidates than on the non-impaired ones; it would be counter-productive and certainly not fair |
| 31 | Y | Mild physical exercises with a set goal of performances were part of the original Russian training syllabus. It strengthens the motivation for achievements/improvements. A similar demonstration exercise should inform the candidate astronauts about the physical challenges expecting them. Orientation may be the age staggered scale of the German Sportabzeichen. Preference should be given to the somewhat space related sports disciplines, i.e trampoline, balance, force. | Y | I assume that the candidate Parastronauts manage well with their disabilities, including physical condition. They will expect that it is asked to demonstrate this training level at one point in the selection, it is where they can excel and gain positive self-consciousness | N | To not create an unbalance between the candidates selected in the Parastronaut and non-parastronaut flow it is mandatory to have them go through the same procedure. |
| 32 | Y | physical performance is a column of human health, furthermore an enhanced, maybe optimized at least a sufficient physical (and mental) performance could make the difference in any case of contingency especially in live threatening situations | Y | It depends on the contents and methods of the test | N | To get a baseline, to collect data, to be able to assess the performance of impaired candidates in relationship to the non-impaired candidates it would be very wise to let the non-impaired candidates make the test. |
| 33 | NS | physical performance is not the only aspect for astronaut readiness | NS | physical performance is not the only aspect for astronaut readiness | N | every astronaut candidate needs a physical performance test |
| 34 |  |  |  |  |  |  |
| 35 | Y | 1- because it was done during the last ESA astronaut selection of 2009. A physical performance test (running test on a treadmill) was implemented for all astronaut candidates during the Medical Screening Phase in hospital before the interview runs. 2- because this is the only way to assess the performance of the response of the cardiovascular system of the candidates to an increasing physical effort. 3- because I performed this test as astronaut candidate in 2009 and know what it is about. 4- because this test has proven to give a valuable information about the physical performance of an astronaut and is implemented yearly for each ESA astronauts. 5-because a similar test is yearly required for the medical assessment of ESA staff. So, there is no reason to have astronaut candidates not tested as such. | Y | The physical efforts and fitness capability required to perform astronaut training tasks and to perform professional astronaut duties in orbit will not drastically change for an impaired astronaut candidate from ESA. As the ESA parastronaut project aimed to select professional astronauts and not tourist flight participants, the duties of the candidates to be selected will require physical performance capabilities to the one of the current ESA astronauts. There is no reason why a physical performance test could not assess the fitness readiness of physically impaired astronauts. here again it is about testing the cardiovascular system response adaptation to a physical effort. Of course, the type of test shall be adapted to the physical impaired candidates, so that the physical handicap allows the test: as the impairment accepted in the selection is related to the legs, the physical performance test should be done using the arms to produce the expected effort for the test. | N | 1- because it was done during the last ESA astronaut selection of 2009. A physical performance test (running test on a treadmill) was implemented for all astronaut candidates during the Medical Screening Phase in hospital before the interview runs. 2- because this is the only way to assess the performance of the response of the cardiovascular system of the candidates to an increasing physical effort. 3- because I performed this test as astronaut candidate in 2009 and know what it is about. 4- because this test has proven to give a valuable information about the physical performance of an astronaut and is implemented yearly for each ESA astronauts. 5-because a similar test is required for the medical assessment of any ESA staff candidates about to be hired by ESA and for ESA staff members each year. So, there is no reason to have astronauts selected without having performed such a test. |
| 36 |  |  |  |  |  |  |
| 37 | Y |  | Y |  | N |  |
| 38 | NS |  | Y |  | N |  |
| 39 | Y | It can contribute to it | NS | I think all participants should be tested on their physical performance and fitness equally. Performance is based on the environment - some accommodations could be thought of. | NS | As above - no difference to other astronauts - all assessed equally - although it could be looked at if some accommodations were made an athlete with a physical disability could participate better. Performance is dependent on the environment to a great deal. |
| 40 | Y | 1. to ensure the crew can withstand G-forces of launch /re-entry/landing. 2. to ensure the crew can self-evacuate in case of a contingency on the launch pad or remote /off nominal landing - including water landing /be able to swim 3. the crew can perform EVA (nominal scheduled EVA and contingency repair EVA) and work with the standard toolkit/ hardware and able to use the EMU/spacesuits for- EVA - both ISS/lunar. 4. the crew is able to perform nominal and contingency operations on ISS / Gateway - so be able to work with on board tools/equipment/emergency kits 5. the crew should be able to use the on-board countermeasure equipment - to maintain good health and performance | Y | For the exact same reasons as for any astronaut candidate -see above: 1. to ensure the crew can withstand G-forces of launch /re-entry/landing. 2. to ensure the crew can self-evacuate in case of a contingency on the launch pad or remote /off nominal landing - including water landing /be able to swim 3. the crew can perform EVA (nominal scheduled EVA and contingency repair EVA) and work with the standard toolkit/ hardware and able to use the EMU/spacesuits for- EVA - both ISS/lunar. 4. the crew is able to perform nominal and contingency operations on ISS / Gateway - so be able to work with on board tools/equipment/emergency kits 5. the crew should be able to use the on-board countermeasure equipment - to maintain good health and performance | N | No all astronauts should meet the minimum required physical performance |
| 41 | NS | Physical performance must be tested but must be in context with a series of other tests (I'm sure this is the case). The question is where the bar should be set - this is not a question of lowering the bar for the sake of E&D / PC but rather a consideration that impairments on Earth might actually have benefits in space - no legs = less room, less oxygen required, less energy (food) required, less weight and more manoeuvrability in tight spaces in zero-G. | NS | It must be a consideration, see comment (above). | N | Surely all candidates must be tested on their physical performance? There are fundamental tasks that must be performed, and all astronauts must meet the criteria to achieve them. |
| 42 | NS | On the one hand, it may be beneficial, on the other hand, you could increase your fitness with a few weeks of more intense exercise. | NS | On the one hand, it may be beneficial, on the other hand, you could increase your fitness with a few weeks of more intense exercise. | NS | No, this would violate the law of equality |
| 43 | Y | Healthy body means healthy mind. Being fiscally fit will give you the ability to think clearly under pressured environments. | Y | Yes, similar than above answer and I can only assume the there will be some physical demand by launching in to space. So, it’s deemed necessary to be fit. | N | Everyone should be measured equally |
| 44 | Y | Determination of the ratio requirement flight performance astronaut Physical performance as a predictor of overall performance capability | Y | Determination of the ratio requirement flight performance astronaut Physical performance as a predictor of overall performance capability | N | Flight requirements apply equally Physical performance within the framework of Individual Function will always influence general performance. This applies equally to people with and without impairments |
| 45 |  |  |  |  |  |  |
| 46 | NS | Hard to comment without further explanation what the 'Physical Performance' test involves? ? e.g., cardiovascular fitness, strength, co-ordination, endurance, task specific skills??? I'm assuming you mean 'physical performance' as one aspect in a full suite of measures? I expect it could be considered in a similar way to pre-season for elite athletes- there are general requirements necessary to play the sport and sports specific skills that need to be considered. For the astronaut -I'm guessing their life may depend on their physical capabilities. So, the 'physical performance test' would need to assess the capabilities required not only for spaceflight - but I would assume - specific to the mission requirements. As for 'readiness' - the tests should reflect the requirements of the tasks. | NS | In line with answer to question 1 - this would depend on the types of physical tests being considered as well as alignment with the specific requirements of the mission | N | I'm guessing physical performance testing is equally essential for all astronauts |

Table 3: Disadvantages of a Physical Performance Tests

|  | **QB1** |  | **QB2** |  | **QB3** |  | **QB4** |  |
| --- | --- | --- | --- | --- | --- | --- | --- | --- |
| **ID** | **Do you see any advantages of astronaut candidates to be subjected to physical performance tests?** | **Comment** | **Do you see any disadvantages of astronaut candidates to be subjected to physical performance tests?** | **Comment** | **Do you see any advantages of physically impaired astronaut candidates to be subjected to physical performance tests?** | **Comment** | **Do you see any disadvantages of physically impaired astronaut candidates to be subjected to physical performance tests?** | **Comment** |
| 1 |  |  |  |  |  |  |  |  |
| 2 | Y | it should be just a regular part of the selection process, for all candidates; Astronauts are expected to perform at peak level; a physical performance assessment is part of the yearly routine; Candidates can then verify their status themselves. | N | no disadvantages. |  | On top of what I already commented for the astronaut candidates: the other crew members don't have to take into account the physical impairment, if they know the performance of the 'parastronaut' is not affected. | N |  |
| 3 | Y | Yes, because if you have a panoply of test, you can show and see what are the weak and strong part of the candidate The good part of working in a team is mixing the weak and strong points of each one to end up with a solid group of work. | N | After having answer the other questions, I understand you are speaking about astronaut candidate without impaired physical body No it is always good to test ... even if we find a problem might be good for the person so we could solve issue | Y | Same as the previous one, when you have weak points on one side on some other side you might have really strong point. This is why having a battery of test looks really good to understand the real abilities of the impaired body So what is really needed at the end is to understand what is the real minimum physical status you need to become this astronaut despite your impaired physical status | Y | Of cause for a candidate with impaired physical body this performance tests might be negative for them because of their health problem but it will only depend once more on the criteria you established at the beginning |
| 4 | Y | 1. Assesses them for the vigour of space. 2. Compares them with a standard, so future generations can be more easily assessed 3. Allows comparison of able bodied and disabled astronauts to assess difference if any. | Y | Exceptional candidates with minor things wrong that may have no effect whatsoever ruled out of taking part. | Y | 1. allows comparison with able- bodied candidates to see the true effect of disability. 2. Allows physically impaired candidates to prove that they are just as good if not better than some able- bodied candidates. | Y | small risk of space exacerbating existing problems. |
| 5 | Y | Assessment of the health and the ability to perform physical tasks over a long period of time. | NS | There is a minor risk of injuries or other medical incidents. However, this risk is acceptable and not higher than in the normal everyday life a physically active candidate. | Y | Same as for other astronaut candidates. | Y | Depending on the type of performance test and the physical impairment, the risk of injuries may be higher if the candidate is not used to particular activity. |
| 6 | Y |  | Y | There is risk of injury in any fitness test. however, the risk can be outweighed by the benefits if those fitness test with sizable risk are done at later stages of the recruitment, so that only a smaller number of applicants is exposed to the test |  |  | Y | It might hurt personal feelings if those individuals cannot do what they are asked to do. However, that disadvantage can also be regarded as an advantage, as it will filter out those impaired applicants that have good coping strategies |
| 7 | Y | It will help them to establish whether they have the necessary fitness | Y | They might expose concerns about which the candidate might have been previously unaware | Y | The tests might identify new aptitudes and skills | N |  |
| 8 | Y | An adequate status of fitness often translates to an optimal health status. Testing physical performance provides a valuable insight on the individual dedication to a healthy way of life, which is required for the astronaut profession | N | No cannot see any disadvantages in assessing physical performance and acquiring a valuable insight on the individual dedication to a healthy way of life which is required for the astronaut profession | Y | Irrespective of physical impairment status, optimal fitness often translates to an optimal health status. Testing physical performance provides a valuable insight on the individual dedication to a healthy way of life which is required for the astronaut profession | N | No cannot see any disadvantages in physically impaired astronauts in assessing physical performance and acquiring a valuable insight on the individual dedication to a healthy way of life which is required for the astronaut profession, |
| 9 | NS | Physical performance can be trained as long as the astronaut is generally fit | Y | An untrained person could have very good capabilities but could fail the test. Just minor training for some weeks / months might bring him already in line with requirements | NS | Again, we need to concentrate on functional tests and ability to perform the task | NS | If we just look at physical performance, we might miss important aspects |
| 10 | Y | see above | Y | This can be seen in disabled candidates due to the kind of disability | N | if the test is adapted to the kind of disability | N |  |
| 11 | Y | Physical performance tests are part of the physiological and psychotechnical evaluation of the candidates. This is obviously an important criterion of the selection process. | N |  | Y | It would help to understand if specific impairments are an issue in the various space facility environments, which again are designed for unimpaired persons. | N |  |
| 12 |  |  |  |  |  |  |  |  |
| 13 | Y | see answer to first question: A basic level of fitness will be required for an astronaut (no difference between traditional and parastronauts). Some have argued in the past that such a test is not necessary, because fitness can be built up and trained at any time. Thus, it was argued that even candidates with a substandard level of fitness can be selected and be trained up later on. However, I do not agree with that. I think that in addition to learning about the current state of fitness of an applicant, such a fitness test would also be an indicator of lifestyle of an applicant. If they did not maintain a basic level of fitness at the time of their application, it is also more likely that they will stop maintaining that level of fitness at a later stage. | N | But the tests should only be testing basic fitness (e.g., cardio and a few strength tests), not a high performance. | Y | see above. Additionally, it might potentially reveal issues that could come up, resulting from the individual physical impairment of the candidate. E.g., in conjunction with the spacecraft configuration or emergency egress capabilities. This would allow for an early addressal of the issue. | N |  |
| 14 | NS | I don't see a real advantage, since physical performance (endurance and strength) are no real requirements for the tasks astronauts have to perform inside the ISS. Physical performance assessment will be an advantage to ensure the readiness of an astronaut candidate to be selected to perform and EVA. In light of future exploration missions (Moon, Mars, Asteroids), surface exploration EVAs in partial-g circumstances will be an important part of the mission and to select the astronauts for those missions’ physical performance tests will provide an advantage. | NS | It depends on the selection criteria linked to the physical performance test... In case a perfectly valid candidate will be rejected because he/she performs under average on the physical performance test (endurance and strength), then I see this as a disadvantage. | NS | I don't see a real advantage, since physical performance (endurance and strength) are no real requirements for the tasks astronauts have to perform inside the ISS. Physical performance assessment will be an advantage to ensure the readiness of an astronaut candidate to be selected to perform and EVA. In light of future exploration missions (Moon, Mars, Asteroids), surface exploration EVAs in partial-g circumstances will be an important part of the mission and to select the astronauts for those missions’ physical performance tests will provide an advantage. | NS | It depends on the selection criteria linked to the physical performance test... In case a perfectly valid candidate will be rejected because he/she performs under average on the physical performance test (endurance and strength), then I see this as a disadvantage. |
| 15 | Y | Minimisation of risk leading to a higher likelihood of mission success. | Y | A physical performance test can never fully replicate the demands of the actual intended environment, and the disadvantage of having performance tests is the small chance that a perfectly suitable candidate fails the test whilst actually being suitable for operating in space. | Y | Minimisation of risk leading to a higher likelihood of mission success. | Y | A physical performance test can never fully replicate the demands of the actual intended environment, and the disadvantage of having performance tests is the small chance that a perfectly suitable candidate fails the test whilst actually being suitable for operating in space. For the astronaut candidate with a physical impairment, this has the potential to be exacerbated based on time and the potential impact that rehabilitative effort can have on the individual's performance. For example, rehabilitation programme and the assistive technology that an individual with an impairment has been provided with plays a large role in that individuals functional outcome, and therefore if a perfectly qualified candidate has not received an appropriate level of rehabilitation therapy or the correct assistive device prescription, and the physical performance test is conducted too early in the candidate recruitment process, there is a higher risk that that individual may fail, whilst in fact being the most appropriate, save for some relatively facile remedial steps. |
| 16 | Y | Screening out unsuitable candidates Ensuring the astronauts have the best possible chance of coming back unharmed | N |  | Y | If anything, they should be subjected to more tests, as their impairment in space is an unknown quantity at this stage | N | Same as above |
| 17 | Y | As previous - they should confirm general wellbeing and test task specific to the environment. | N | It is logical to assess fitness to undertake the tasks required in the environment. | Y | It is logical to assess fitness to undertake the tasks required in the environment. As discussed previously they need to consider whether the test condition on earth can replicate test condition that would be in space. | N | Important pre-assessment with previous proviso noted |
| 18 |  |  |  |  |  |  |  |  |
| 19 | Y | To make sure they are physically capable of enduring space travel. | N | As long as they are pre warned for the test, they can be prepared and excel. | Y | No para candidate wants to be baggage. They will want and need to be a vital member of the team. Physically testing them shows they are expected to pull their weight. | Y | The test must be adapted to allow the candidate a fair chance of passing the test. This will need careful consideration. |
| 20 | Y | The main advantage would be to ensure that the candidate is able to cope with the physical needs of a spaceflight, including 1) in-flight operations and the cost they exert on the body (example: EVA) but 2) more importantly, cases where an astronaut may need to be physically fit to save their live or the lives of their crewmates. An example of the latter is a launch emergency that requires the crew to evacuate the launch tower immediately or a landing emergency where the crew has to survive on water or land before being rescued. | NS | An obvious disadvantage is the monetary cost for the tester (selecting Agency) and the extra time requirement and stress exerted on the tested candidates. I cannot think of any other disadvantages. | Y | Yes, exactly the same as for non-impaired candidates. Here we presume that the importance of the test may be higher to ensure that an impaired person can survive the adverse conditions of emergency scenarios. | NS | The same as for non-impaired candidates. An extra disadvantage may be that in case only impaired candidates are asked to perform this test, this may create a feeling of inequality between them and their non-impaired co-candidates. |
| 21 | Y | Assuming that there are minimal physical performances that are required (also ethically) to ward off possible dangers to oneself and others, a physical performance test naturally has the advantage of making the achievement of these minimal performances testable (within reason). | N | At most, if the tests measure something that is not really important or use too high (unrealistic) minimum values. | Y | The same as for non-impaired candidates: if there are threshold values to be achieved (and possibly also adapted to the disability), this can be tested by this. | N | Only if the tests impose unnecessary or unrealistic requirements and were not developed on questionable data, or if accommodations for disability are omitted even though they could be justified (although there may be cases where accommodations are not possible). |
| 22 | Y | stress (cardio) test as a minimum. some hand grip and arm force/torque performance tests might be useful | N | No if the tests are not intended to rank candidates but rather to ensure they have minimum required cardio performance (and possibly minimum upper body force/torque performance) | Y | same for all candidates (see 2nd previous question) | N | No if the tests are similar to those for all candidates (cardio tests) and if the tests reference data are based on the same requirement. (impaired candidates might have to use a hand pedal machine rather than a foot pedal machine, therefore a reference norm must be established) |
| 23 | NA |  | NA |  | NA |  | NA |  |
| 24 | Y |  | N |  | Y |  | N |  |
| 25 | Y | Yes, if the physical performance test is based on occupational requirements (i.e., essential tasks), then both the astronaut candidate and support team would have an objective measure of the astronaut candidate's readiness. It would also serve to remove any real or perceived bias. | Y | There is a potential for injury while undergoing the physical performance test. | Y | Yes, if the physical performance test is based on occupational requirements (i.e., essential tasks), then both the astronaut candidate and support team would have an objective measure of the astronaut candidate's readiness. It would also serve to remove any real or perceived bias. | Y | There is a potential for injury while undergoing the physical performance test |
| 26 |  |  |  |  |  |  |  |  |
| 27 | Y | It is the only way to actually see and measure how people perform on which you can rely the selection. Performance criteria need to be public and enough time in advance to prepare! | N | Being able to perform good in a physical performance test/battery on ground is key to avoid harmful degradation of the human body exposed to microgravity. | NA | Equal treatment for all | NA | Equal treatment for all |
| 28 |  |  |  |  |  |  |  |  |
| 29 | Y | health and fitness are a basic requirement for the job of an astronaut. | N |  | Y | health and fitness are a basic requirement for the job of an astronaut. | N |  |
| 30 | NS | since people are trainable, I don't think this would add much to a selection - provided the people are healthy | Y | we might lose people with excellent skills just because they are not as fit, while fitness can be trained after selection | NS | yes, if the test was limited to reach / on-board or training related characteristics; however, my negative assessment of testing only impaired candidates stands against this. | Y | additional burden placed on impaired persons |
| 31 | Y | Yes, but not to select them out, but to acquaint them with the physical challenges. | Y | Yes, it will be difficult to find reasonable standards for passing the tests wrt. age, body height, and choice of disciplines to perform. | Y | Yes, they will expect to be asked to show their level of self-sustained living, also involving making up for their disabilities. | Y | Yes, if it is used applying unreasonable physical performance limits, where otherwise mentally and intellectually able candidates may accidentally fail to meet the set criteria. |
| 32 | Y | see question and answer before | NS | That might take some effort and time... | Y | ESA is responsible for meeting the NASA standards while assigning astronauts for increments. The International Partners must count on the availability of physical and mental fitness and performance of European astronauts. Health and performance constraints could risk the health of IP astronauts, safety and mission goals. | N |  |
| 33 | Y | no comment needed, its logic | N |  | N | no comment needed, its logic | N |  |
| 34 |  |  |  |  |  |  |  |  |
| 35 | Y | -because this is the only way to assess the performance of the response of the cardiovascular system of the candidates to an increasing physical effort. -because this test has proven to give a valuable information about the physical performance of an astronaut and is implemented yearly for each ESA astronauts. - because being able to cope with a long-lasting physical effort is required for astronauts’ professional duties. | N | the only potential disadvantage for an astronaut candidate subjected to physical performance tests in the selection process could be to run the risk to show to the medical board that this candidate is physically unfit for astronaut professional duties. But this is not a disadvantage for ESA (on the contrary, it is all what the selection is about) and for the astronauts selected in fine, as they would be fit for their duties. | Y | Here again: if selected as a professional astronaut, a physically impaired candidate will have to be fit for his/her astronaut duties. It is an advantage to have this fitness assessment done in the selection to ensure that the selected parastronauts will be fit for their duties (training and in --orbit tasks). Nobody in the astronaut selection board of ESA would like to select an impaired astronaut that would be identified after the selection as not fit for his/her future job. | N | No, as long as the test is adapted to the impairment of the candidates. Here again, the response performance of the cardiovascular system to an increasing effort can be tested using the arms to produce the effort instead of the legs, if the impairment is on the legs. |
| 36 |  |  |  |  |  |  |  |  |
| 37 | Y |  | N |  | Y |  | N |  |
| 38 | Y |  | Y |  | Y |  | Y |  |
| 39 | NS | Depends on the purpose - would need to know more to answer | Y | As said - performance depends not just on the person but also on the performance and in which the person needs to do something. | NS |  | Y | If only physically disabled athletes were tested this would pose the question, why? and why not the non-disabled candidates. |
| 40 | Y | It helps to obtain the full picture of the astronaut skillset | Y | You really have to make sure that the test is representative and realistic for the actual work and contingency skills the astronauts need to have - if you set the bar to high - you may lose good candidates - if you set the bar to low - you will be confronted with disabilities too late | Y | Same as for any candidate | Y | Same as for any candidate; see answer above Plus: need to consider if ESA is willing to supply extra tools / hardware/ equipment/ custom-made suits to compensate for a specific impairment - then these need to be available at the performance test stage as well. |
| 41 | Y | There are fundamental tasks that need to be completed. The small crew must be able to rely on each other and not be concerned that one member is a 'weak link'. | Y | Tests can create binary results - pass/fail - which may exclude some people that have a lot to offer in other areas, especially if there are adaptations that could be made (either by the individual or by the organisation) to allow these additional offers to come to the fore. | Y | If the tests are fair and true representations of the fundamental tasks required of astronauts, then those passing the tests will have unequivocal proof of their suitability. The key lies in the test design. | Y | The tests must be designed with a progressive mindset - 'how can we get the most out of people' - not with a mindset of status quo - 'disabled people can't go into space'. |
| 42 | NS | it depends: Of course, you may notice physical problems or fitness issues that you would not notice and that could prove to be very negative later on. On the other hand, you need resources, and you have to calculate the costs (personnel, working hours, possible travel expenses, etc.) | NS | At the moment I can see only one disadvantage: The costs (personnel, working hours, possible travel expenses, etc.). | NS | You may notice physical problems or fitness issues that you would not otherwise notice This may prove to be a positive way to perhaps resolve the issues or see that these issues do not allow for continued participation | N |  |
| 43 | Y | But also, the candidates’ injuries to be take on consideration. | NS |  | Y | But for candidates’ impairments to be taken in consideration. | NS |  |
| 44 | Y | Selection criterion Protection of candidates from unfeasible requirements during a flight | N | Ultimately, I do not see the exclusion for the protection of the person as a disadvantage. Nevertheless, it is a selection that is personally perceived as negative. | Y | Selection criterion Protection of candidates from unfeasible requirements during a flight | N | Ultimately, I do not see the exclusion for the protection of the person as a disadvantage. Nevertheless, it is a selection that is personally perceived as negative. |
| 45 |  |  |  |  |  |  |  |  |
| 46 | Y | Please see comments above; as long as the tests are functional and relevant then my view would be that physical performance tests would enhance astronaut safety. I would hope that good candidates would receive training to improve any deficits that were identified - rather than this be used as a hard 'entry requirement' to the program. | Y | Depends how the results are used - as above - if the tests are designed to ensure astronaut safety (screening physical readiness and ability to perform tasks that are required for the mission) - as long as candidates knew what was expected of them - and were given the opportunity to improve any tests that they didn't pass. A disadvantage would be if the tests eliminated good candidates unnecessarily, e.g., if the tests were too difficult and not aligned to tasks required of astronauts | Y | To ensure the astronaut's safety - I'm guessing there are physical requirements that need to be assessed | Y | Please see comment above - I would view this as being the same for all astronaut candidates |

Table 4: Raw data of the first Delphi round for questions adressing categories 3 and 4

|  | **QC1** |  | **QD1** |  |
| --- | --- | --- | --- | --- |
| **ID** | **Do you have any concerns about using a physical performance test as a tool to ensure operational readiness as part of the astronaut selection?** | **Comment** | **In your opinion, which skills should be tested with physical performance tests to ensure operational readiness during missions and mission training?** | **Comment** |
| 1 |  |  |  |  |
| 2 | Y | Things that are hard to verify during a physical performance test: correctly using a launch/re-entry spacesuit; correctly using EVA suits and tools; correctly executing emergency procedures that require physical mobility. | The following skills should be tested... | egressing: a spacecraft seat from a supine position; water egress; HUET training; underwater skills proficiency. |
| 3 | Y | Yes and no, .. just want to say cannot be the only test of course psychology is very important and regarding impaired body .. on the other side it is well known with physical impairment, the person will have probably higher level of consciousness of their body if only physical performance we might refuse good candidate with strong minds so other tests should be done | The following skills should be tested... | memory all neurological skills like orientation, patience teamwork abilities motivation challenges (.. like being innovative and creative expose to test with testing the fatigue and frustration |
| 4 | N |  | The following skills should be tested... | 1. manual dexterity. 2. Manoeuvrability. 3. Proprioception 4. Physically stressed Decision- making |
| 5 | N | No concerns as long as the test is validated and relevant (e.g., treadmill or rowing ergometer) | I don't think special skills need to be tested to ensure operational readiness during mission and mission training. | Most skills can be learned and may give a false impression of the candidate. However, physical endurance is relevant from a health assessment perspective. Para-astronaut candidates may have challenges related to learning certain skills, and these should be invited to demonstrate tests that are tailored to the particular handicap. |
| 6 | Y | yes, see my Response to the previous question | The following skills should be tested... | Ambulatory capacity; dexterity; muscle strength and power; aerobic fitness |
| 7 | N |  | The following skills should be tested... | I am sure that there must be specific skills which are relevant to the task |
| 8 | N |  | The following skills should be tested... | The same skills that are currently being trained and tested in astronauts’ candidates. |
| 9 | Y | Physical performance can be easily enhanced with training as long as the candidate is sufficiently fit | The following skills should be tested... | hand eye coordination, general aptitude to train and perform exercise |
| 10 | N |  | The following skills should be tested... | movement ability in weightlessness, orientation when being in weightlessness he or she must be able to float within the ISS without problems, road of floating mast be under control |
| 11 | N | This is a major requirement to ensure mission success and to prevent major issues or concerns to the other crewmembers during a space mission. | The following skills should be tested... | I think health and fitness tests currently required and used for the ISS missions (preflight and on-orbit) are well adapted to guarantee the operational readiness from a physical standpoint. |
| 12 |  |  |  |  |
| 13 | NS | I don't think a physical performance test indicates "operational readiness". I think it indicates that the candidate can be trained up towards operational readiness if required. In addition, it indicates issues with their lifestyle that might prevent their operational readiness at a later stage. | The following skills should be tested... | Aptness to a positive response to a training stimulation. Cardio response. Maybe balance tests. General good awareness of their body and capabilities. Range of motion (might be important for EVA tasks in a suit). |
| 14 | Y | In case the 'pass criteria' of the physical performance test is set too high, you might lose otherwise perfectly valid astronaut candidates from the selection. | The following skills should be tested... | Motoric skills: hand-eye coordination, general hand/arm motoric skills and single and double handed operations skills. |
| 15 | Y | Yes - if the process and the outcome potential of enhanced rehabilitation is not understood in the case of the astronaut candidate with a physical impairment then suitable candidates may be overlooked. | The following skills should be tested... | Cardiovascular endurance Muscle strength (pre-mission muscle volume to account for disuse atrophy) Climbing ability (particularly ladders) Navigation of small and confined spaces Standing and dynamic balance and fine motor control skills For those using assistive devices (particularly prosthetics, from my experience): Spatial awareness Skin health of residual limb Prosthetic fitment, particularly comfort levels and adjustability Ability to wear prosthetics for extended periods of time at high activity levels |
| 16 | N |  | The following skills should be tested... | Mobility cognitive ability (problem solving, decision making) co-ordination stress management |
| 17 | N | Exercise or performance testing is standard practice in Paralympic athletes with a wide range of physical impairments. the challenge will be adapting any operational task specific test to make it relevant, but I do not believe this to be impossible. | Not sure | I am unsure of the skills because of lack of familiarity with the tasks required in the space environment but could help advise with greater knowledge of this. |
| 18 |  |  |  |  |
| 19 | N | All crew members must be physically capable. | The following skills should be tested... | Ability to endure high g-force without the disability reducing capability. Strength to conduct regular tasks in missions. Ability to endure long hours in difficult environments. |
| 20 | N | The only serious concern i can think of is over-confidence in the procedure and result: A candidate that passes such a test will in most likelihood be able to sustain the needs of a spaceflight and save their own and their crew's lives in adverse situations if needed. But that should not create on the Agency's side the confidence that a person who passes the test necessarily needs no extra help or retesting down the road. Or repeat testing if a lot of time has passed between selection and mission assignment. | The following skills should be tested... | I think fitness, range of motion, basic sensorimotor skills should be tested and found adequate for a candidate to pass. Adequate does not mean "perfect": if an impaired candidate has means of coping, or if the Agency can find means of supporting such people when flown to space, then that person should not be disqualified solely on the grounds of a low result in the physical performance test. |
| 21 | N | Not fundamentally, much depends on the nature of the test, its quality (on what evidence base developed?), possible threshold values (set realistically and thus "fairly"?), and possible adjustments for impairment (if justifiable). | Not sure | If there is a justification in terms of what tasks are then to be performed (depending on the role/function), or what reactions the skill should enable, that could be specifically tested. Again, it should be avoided to test something for which there is no good data basis as to why this basically needs to be mastered as an astronaut candidate. |
| 22 | NA |  | NA |  |
| 23 | NA |  | NA |  |
| 24 | NA |  | The following skills should be tested... | several skills should be tested with physical performance test, including osteo-articular capability, muscle strength, prehensility |
| 25 | N | As long as the physical performance test is based on the occupational requirements of the position, I do not have concerns about using a physical performance test. This means the test would be designed based on essential tasks that are physically demanding. Essential Tasks for CSA astronauts are defined as tasks where failure to safely and efficiently perform the task could result in: – Compromised mission/operation, – Result in injury/death to personnel on the ground or in space and/or – Cause significant loss/damage to ground property or space assets. | The following skills should be tested... | CSA is in the process of identifying what these skills are for our astronauts. We hope to have these skills identified over the next year. |
| 26 |  |  |  |  |
| 27 | Y | We should not use hard limits for a successful physical performance test but rather soft limits where the subject matter experts see the whole individuum/test performance and decide based on all aspects if GO for becoming an astronaut. | NS | However, the physical performance tests should reflect a reasonable amount of performance skills which are relevant for a mission. E.g., proper exercise technique for daily CMS training. |
| 28 |  |  |  |  |
| 29 | N |  | NS | ability to live and work in microgravity |
| 30 | Y | my concern is that we would not test for aptitude but for something trainable already there we will potentially loose better candidates based on lower physical performance that is not really required on-board | I don't think special skills need to be tested to ensure operational readiness during mission and mission training. | |
| 31 | N | No, physical exertion brings about mental stress and can reveal otherwise hidden doubtful personal traits conflicting with wanted astronaut characteristics | The following skills should be tested... | In general, the communicated limits should be set just outside comfort levels, to test the willingness of the individuals to also invest physical capital into their astronaut application. g-forces, balance, and endurance come to my mind. Typical track and field exercises however should be adapted to show a standard level of physical readiness, not reach into advanced demands or require special techniques. |
| 32 | N |  | The following skills should be tested... | It's much too late to discuss about this topic. There should have been made an analysis, which skills needed to have for mission and mission training. An astronaut candidate should endure every part of the mission training or mission without the help of anybody else e.g., sea survival, vehicle egress, rescue operations etc. |
| 33 | N |  | The following skills should be tested... | health, medical status and functional capabilities: Lab values, Drug screen, Eye exams, Cardio and fitness, Gastroenterology, Neurology and Psychology, female: Gynaecology, Breast MRI, Chest X-Ray, Radiation history, dental exam + Jaw X-ray, possibly: nutrition, fatigue countermeasures |
| 34 |  |  |  |  |
| 35 | N | I have no concern at all. Why shall anyone have suddenly a concern, while physical performance tests have been systematically part of all the astronauts selections by all space agencies selecting astronauts? NASA even had such physical performance tests in the last year NASA astronaut selection and ESA did the same in 2009. | The following skills should be tested... | Adaptation of the cardiovascular system to an increasing physical effort, also known as Cardiopulmonary Exercise Test (CPET). this test shall be performed with ECG, blood pressure measurement and breathing gas measurement (O2, VO2) and shall help to measure the VO2max. |
| 36 |  |  |  |  |
| 37 | Y |  | NA |  |
| 38 | N |  | The following skills should be tested... | strength, coordination, balance, body control |
| 39 | N |  | NS | I am not knowledgeable enough what skills and fitness level are needed to be an astronaut. |
| 40 | Y | As mentioned earlier You need to validate the test, ensure is it representative and realistic for all potential vehicles (CCP/ ISS/ Lunar etc) and equipment /suits the astronauts would need to work with - so there is a risk the bar is either set too high or too low. | The following skills should be tested... | 1. swimming 2. evacuate out of a (mock up) crew module on launch pad/ and in water 3. grip strength (based on actual tools / EMU glove pressures) 4. able operate standard tools, emergency equipment and countermeasure H/W 5. able to use the standard handles (opening/closing) hatches 6. use bars-or clips- for stabilizing one’s position in microgravity (zero G flight?) 7. able to don/doff flight suit/EMU |
| 41 | Y | Concerns outlined in previous comments. | NS |  |
| 42 | N |  | NS | This question should be answered by sports scientists together with orthopaedists/neurologists/physiotherapists, they can answer this question better than I can. |
| 43 | N |  | NS |  |
| 44 | N |  | I don't think special skills need to be tested to ensure operational readiness during mission and mission training. | I suspect that in all tests of potential astronauts, the areas of endurance, strength and coordination find a central place. I would consider speed and flexibility to be of secondary importance, but not negate their significance. |
| 45 |  |  |  |  |
| 46 | Y | Please see previous comments. Tests would need to be specific to the tasks, fair and candidates would need to know beforehand what was expected of them. I believe the opportunity to address any identified impairments would be ideal. And obviously - test results would need to consider within the battery of other tests that are used to select the best person for the job. | The following skills should be tested... | Please see previous comments. I would expect that as biological systems are known to adapt in space - and especially in consideration of the effects of long-duration missions in the future - tests could be based on the systems that change most in space (e.g. - antigravity muscles are known to be primarily affected). 'Skills', on the other hand, is a different and more complex consideration than 'physical performance.' As mentioned previously - physical performance has many aspects - e.g., strength, endurance, C-V fitness, co-ordination, motor control, proprioception....etc etc - skills are more complex aspect. If 'skills' required for astronaut performance (especially functional tasks) could be broken down into their core physiological/ biological requirements - these aspects could be tested - and physical performance testing could be progressed to test the required 'skills'. If the required 'skill' was not attained by the candidate - a 'layered' assessment process would identify the elements that the candidate needed to improve. |

Supplement 4 – Questionnaire 2


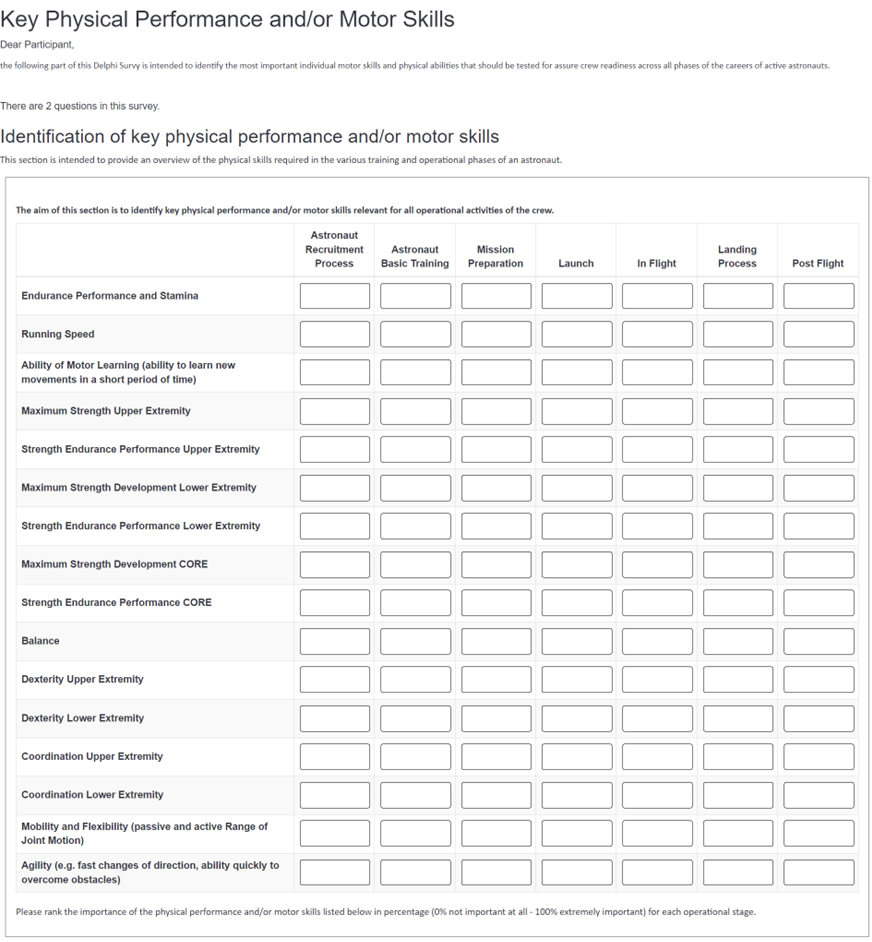


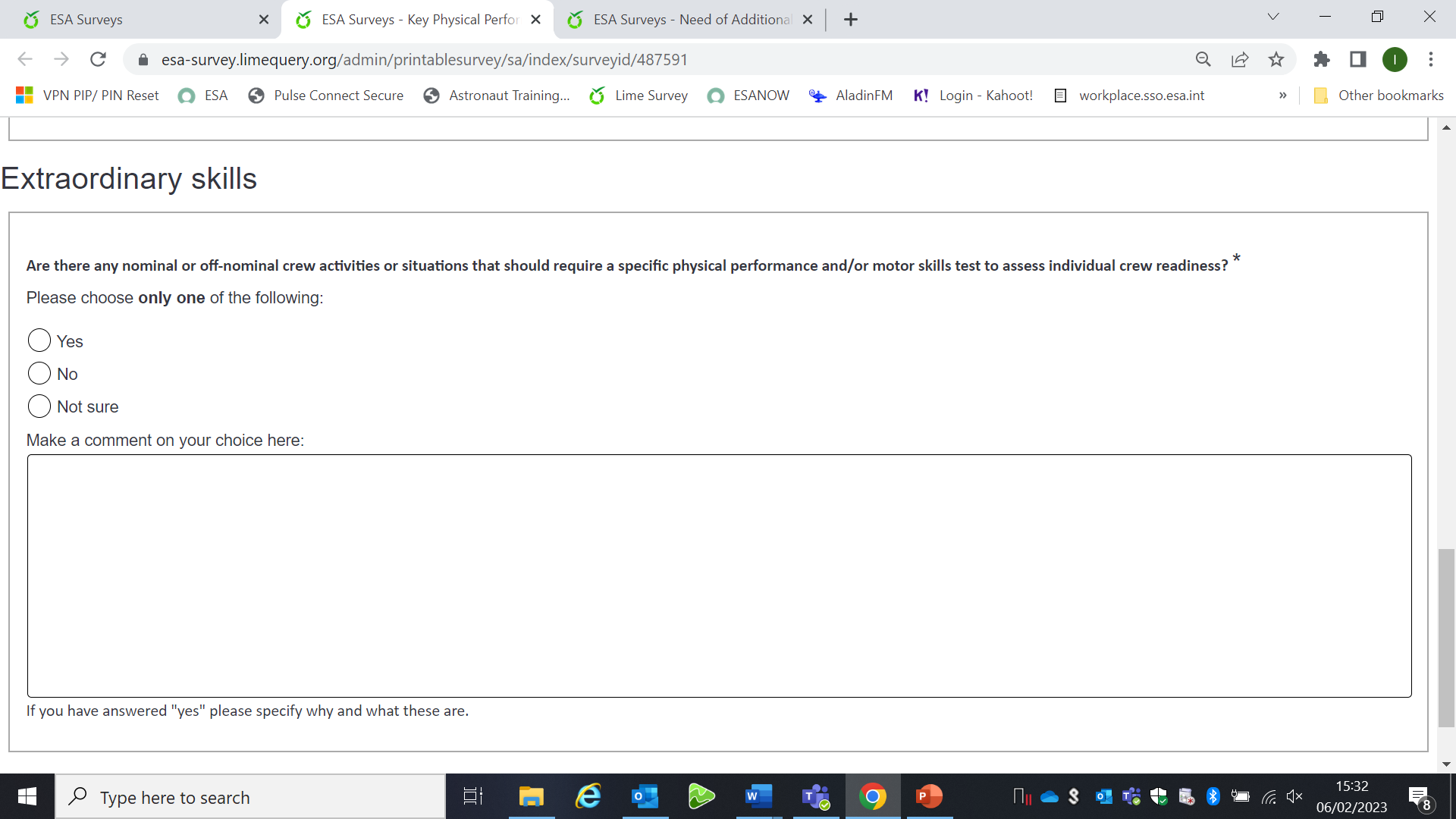


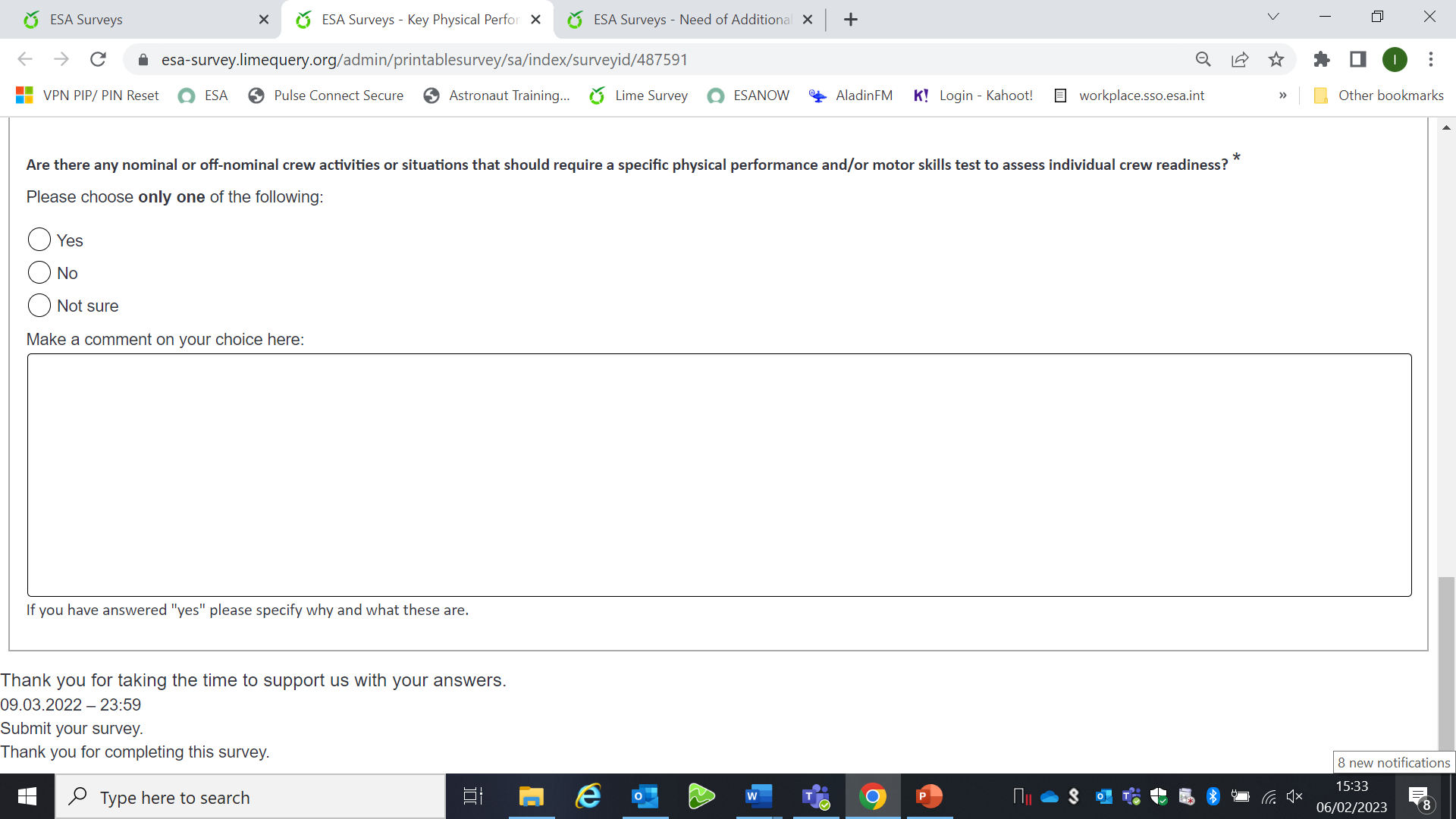


Supplement 5 – Results Round 2

Table 5: Physical Performance Parameters rated by importance for the different career stages of an astronaut – Endurance Performance and Stamina

| ID | Endurance Performance and Stamina [Astronaut Recruitment Process] | Endurance Performance and Stamina [Astronaut Basic Training] | Endurance Performance and Stamina [Mission Preparation] | Endurance Performance and Stamina [Launch] | Endurance Performance and Stamina [In Flight] | Endurance Performance and Stamina [Landing Process] | Endurance Performance and Stamina [Post Flight] |
| --- | --- | --- | --- | --- | --- | --- | --- |
| 6 | 70 | 70 | 70 | 50 | 70 | 50 | 70 |
| 7 | 50 | 70 | 70 | 75 | 10 | 80 | 80 |
| 8 | 10 | 40 | 65 | 65 | 70 | 80 | 60 |
| 9 | 65 | 80 | 90 | 90 | 65 | 55 | 70 |
| 10 |  |  |  |  |  |  |  |
| 11 |  |  |  |  |  |  |  |
| 12 | 85 | 85 | 100 | 75 | 100 | 75 | 75 |
| 13 |  |  |  |  |  |  |  |
| 14 | 50 | 70 | 80 | 80 | 80 | 80 | 70 |
| 15 |  |  |  |  |  |  |  |
| 16 |  |  |  |  |  |  |  |
| 17 |  |  |  |  |  |  |  |
| 18 |  |  |  |  |  |  |  |
| 19 | 90 | 90 | 70 | 50 | 50 | 50 | 50 |
| 20 | 75 | 75 | 75 | 85 | 85 | 85 | 50 |
| 21 |  |  |  |  |  |  |  |
| 22 |  |  |  |  |  |  |  |
| 23 |  |  |  |  |  |  |  |
| 24 | 50 | 70 | 25 | 30 | 20 | 25 | 10 |
| 25 | 40 | 30 | 60 | 30 | 70 | 50 | 20 |
| 26 | 70 | 70 | 70 | 70 | 80 | 100 | 100 |
| 27 |  |  |  |  |  |  |  |
| 28 | 50 | 90 | 90 | 100 | 70 | 100 | 50 |
| 29 |  |  |  |  |  |  |  |
| 30 | 90 | 90 |  |  |  |  |  |
| 31 | 50 | 50 | 75 | 75 | 75 | 100 | 75 |
| 32 |  |  |  |  |  |  |  |
| 33 | 70 | 80 | 80 | 80 | 90 | 80 | 65 |

Table 6: Physical Performance Parameters rated by importance for the different career stages of an astronaut – Running Speed

| ID | Running Speed [Astronaut Recruitment Process] | Running Speed [Astronaut Basic Training] | Running Speed [Mission Preparation] | Running Speed [Launch] | Running Speed [In Flight] | Running Speed [Landing Process] | Running Speed [Post Flight] |
| --- | --- | --- | --- | --- | --- | --- | --- |
| 6 | 10 | 10 | 10 | 10 | 10 | 10 | 10 |
| 7 | 30 | 30 | 25 | 10 | 30 | 30 | 30 |
| 8 | 0 | 10 | 10 | 0 | 0 | 0 | 0 |
| 9 | 50 | 60 | 70 | 70 | 50 | 50 | 60 |
| 10 |  |  |  |  |  |  |  |
| 11 |  |  |  |  |  |  |  |
| 12 | 25 | 25 | 25 | 25 | 50 | 25 | 25 |
| 13 |  |  |  |  |  |  |  |
| 14 | 25 | 30 | 40 | 40 | 30 | 25 | 25 |
| 15 |  |  |  |  |  |  |  |
| 16 |  |  |  |  |  |  |  |
| 17 |  |  |  |  |  |  |  |
| 18 |  |  |  |  |  |  |  |
| 19 | 10 | 10 | 10 | 0 | 0 | 0 | 0 |
| 20 | 0 | 0 | 0 | 0 | 0 | 50 | 0 |
| 21 |  |  |  |  |  |  |  |
| 22 |  |  |  |  |  |  |  |
| 23 |  |  |  |  |  |  |  |
| 24 | 20 | 20 | 20 | 20 | 20 | 20 | 20 |
| 25 | 40 | 30 | 30 | 60 | 50 | 60 | 20 |
| 26 | 70 | 70 | 70 | 100 | 80 | 100 | 80 |
| 27 |  |  |  |  |  |  |  |
| 28 | 50 | 50 | 50 | 60 | 50 | 60 | 50 |
| 29 |  |  |  |  |  |  |  |
| 30 | 50 | 50 |  |  |  |  |  |
| 31 | 50 | 50 | 75 | 75 | 80 | 100 | 80 |
| 32 |  |  |  |  |  |  |  |
| 33 | 20 | 20 | 20 | 40 | 30 | 60 | 20 |

Table 7: Physical Performance Parameters rated by importance for the different career stages of an astronaut – Ability of Motor Learning

| ID | Ability of Motor Learning (ability to learn new movements in a short period of time) [Astronaut Recruitment Process] | Ability of Motor Learning (ability to learn new movements in a short period of time) [Astronaut Basic Training] | Ability of Motor Learning (ability to learn new movements in a short period of time) [Mission Preparation] | Ability of Motor Learning (ability to learn new movements in a short period of time) [Launch] | Ability of Motor Learning (ability to learn new movements in a short period of time) [In Flight] | Ability of Motor Learning (ability to learn new movements in a short period of time) [Landing Process] | Ability of Motor Learning (ability to learn new movements in a short period of time) [Post Flight] |
| --- | --- | --- | --- | --- | --- | --- | --- |
| 6 | 100 | 100 | 100 | 50 | 100 | 50 | 80 |
| 7 | 85 | 80 | 85 | 80 | 80 | 90 | 90 |
| 8 | 20 | 40 | 65 | 50 | 55 | 10 | 40 |
| 9 | 60 | 80 | 95 | 95 | 80 | 70 | 70 |
| 10 |  |  |  |  |  |  |  |
| 11 |  |  |  |  | 100 |  |  |
| 12 | 100 | 70 | 95 | 70 | 100 | 70 | 70 |
| 13 |  |  |  |  |  |  |  |
| 14 | 60 | 60 | 70 | 40 | 70 | 50 | 60 |
| 15 |  |  |  |  |  |  |  |
| 16 |  |  |  |  |  |  |  |
| 17 |  |  |  |  |  |  |  |
| 18 |  |  |  |  |  |  |  |
| 19 | 25 | 40 | 40 | 10 | 30 | 5 | 5 |
| 20 | 75 | 75 | 25 | 25 | 75 | 75 | 75 |
| 21 |  |  |  |  |  |  |  |
| 22 |  |  |  |  |  |  |  |
| 23 |  |  |  |  |  |  |  |
| 24 | 20 | 80 | 50 | 25 | 25 | 25 | 30 |
| 25 | 75 | 80 | 70 | 50 | 80 | 30 | 10 |
| 26 | 80 | 80 | 100 | 50 | 80 | 50 | 100 |
| 27 |  |  |  |  |  |  |  |
| 28 | 50 | 60 | 70 | 70 | 60 | 70 | 50 |
| 29 |  |  |  |  |  |  |  |
| 30 | 70 | 70 |  |  |  |  |  |
| 31 | 80 | 80 | 85 | 90 | 90 | 90 | 90 |
| 32 |  |  |  |  |  |  |  |
| 33 | 85 | 85 | 85 | 50 | 90 | 90 | 50 |

Table 8: Physical Performance Parameters rated by importance for the different career stages of an astronaut – Maximum Strength Upper Extremity

| ID | Maximum Strength Upper Extremity [Astronaut Recruitment Process] | Maximum Strength Upper Extremity [Astronaut Basic Training] | Maximum Strength Upper Extremity [Mission Preparation] | Maximum Strength Upper Extremity [Launch] | Maximum Strength Upper Extremity [In Flight] | Maximum Strength Upper Extremity [Landing Process] | Maximum Strength Upper Extremity [Post Flight] |
| --- | --- | --- | --- | --- | --- | --- | --- |
| 6 | 70 | 70 | 70 | 50 | 70 | 50 | 70 |
| 7 | 50 | 70 | 70 | 50 | 70 | 50 | 50 |
| 8 | 0 | 20 | 50 | 50 | 50 | 60 | 50 |
| 9 | 60 | 70 | 70 | 70 | 80 | 75 | 75 |
| 10 |  |  |  |  |  |  |  |
| 11 |  |  |  |  |  |  |  |
| 12 | 80 | 70 | 80 | 70 | 80 | 70 | 70 |
| 13 |  |  |  |  |  |  |  |
| 14 | 20 | 30 | 40 | 30 | 40 | 30 | 20 |
| 15 |  |  |  |  |  |  |  |
| 16 |  |  |  |  |  |  |  |
| 17 |  |  |  |  |  |  |  |
| 18 |  |  |  |  |  |  |  |
| 19 | 15 | 15 | 15 | 5 | 10 | 5 | 10 |
| 20 | 75 |  | 75 |  | 75 |  |  |
| 21 |  |  |  |  |  |  |  |
| 22 |  |  |  |  |  |  |  |
| 23 |  |  |  |  |  |  |  |
| 24 | 20 | 80 | 50 | 25 | 25 | 25 | 30 |
| 25 | 30 | 40 | 60 | 30 | 80 | 10 | 10 |
| 26 | 70 | 70 | 80 | 80 | 100 | 80 | 100 |
| 27 |  |  |  |  |  |  |  |
| 28 | 50 | 60 | 70 | 70 | 50 | 70 | 50 |
| 29 |  |  |  |  |  |  |  |
| 30 | 10 | 10 |  |  |  |  |  |
| 31 | 50 | 50 | 60 | 70 | 70 | 65 | 65 |
| 32 |  |  |  |  |  |  |  |
| 33 | 60 | 65 | 65 | 50 | 75 | 75 | 20 |

Table 9: Physical Performance Parameters rated by importance for the different career stages of an astronaut – Strength Endurance Performance Upper Extremity

| ID | Strength Endurance Performance Upper Extremity [Astronaut Recruitment Process] | Strength Endurance Performance Upper Extremity [Astronaut Basic Training] | Strength Endurance Performance Upper Extremity [Mission Preparation] | Strength Endurance Performance Upper Extremity [Launch] | Strength Endurance Performance Upper Extremity [In Flight] | Strength Endurance Performance Upper Extremity [Landing Process] | Strength Endurance Performance Upper Extremity [Post Flight] |
| --- | --- | --- | --- | --- | --- | --- | --- |
| 6 | 70 | 70 | 70 | 50 | 70 | 50 | 70 |
| 7 | 45 | 70 | 70 | 70 | 70 | 70 | 70 |
| 8 | 0 | 30 | 50 | 50 | 40 | 60 | 70 |
| 9 | 55 | 60 | 70 | 70 | 65 | 60 | 60 |
| 10 |  |  |  |  |  |  |  |
| 11 |  |  |  |  |  |  |  |
| 12 | 80 | 70 | 80 | 70 | 80 | 70 | 70 |
| 13 |  |  |  |  |  |  |  |
| 14 | 40 | 50 | 60 | 50 | 60 | 50 | 30 |
| 15 |  |  |  |  |  |  |  |
| 16 |  |  |  |  |  |  |  |
| 17 |  |  |  |  |  |  |  |
| 18 |  |  |  |  |  |  |  |
| 19 | 40 | 25 | 25 | 5 | 25 | 5 | 10 |
| 20 | 75 | 75 | 75 | 75 | 75 | 75 | 25 |
| 21 |  |  |  |  |  |  |  |
| 22 |  |  |  |  |  |  |  |
| 23 |  |  |  |  |  |  |  |
| 24 | 20 | 80 | 50 | 25 | 25 | 25 | 10 |
| 25 | 30 | 40 | 40 | 40 | 60 | 20 | 10 |
| 26 | 70 | 70 | 80 | 80 | 100 | 100 | 100 |
| 27 |  |  |  |  |  |  |  |
| 28 | 50 | 60 | 60 | 60 | 50 | 60 | 50 |
| 29 |  |  |  |  |  |  |  |
| 30 | 30 | 30 |  |  |  |  |  |
| 31 | 60 | 60 | 75 | 75 | 80 | 80 | 90 |
| 32 |  |  |  |  |  |  |  |
| 33 | 80 | 90 | 90 | 50 | 90 | 60 | 20 |

Table 10: Physical Performance Parameters rated by importance for the different career stages of an astronaut – Maximum Strength Lower Extremity

| ID | Maximum Strength Lower Extremity [Astronaut Recruitment Process] | Maximum Strength Lower Extremity [Astronaut Basic Training] | Maximum Strength Lower Extremity  [Mission Preparation] | Maximum Strength Development Lower Extremity [Launch] | Maximum Strength Lower Extremity [In Flight] | Maximum Strength Lower Extremity [Landing Process] | Maximum Strength Lower Extremity [Post Flight] |
| --- | --- | --- | --- | --- | --- | --- | --- |
| 6 |  |  |  |  |  |  |  |
| 7 | 60 | 60 | 60 | 30 | 30 | 30 | 65 |
| 8 | 0 | 20 | 30 | 30 | 30 | 10 | 50 |
| 9 | 55 | 60 | 70 | 70 | 55 | 45 | 60 |
| 10 |  |  |  |  |  |  |  |
| 11 |  |  |  |  |  |  |  |
| 12 | 90 | 65 | 90 | 65 | 90 | 65 | 65 |
| 13 |  |  |  |  |  |  |  |
| 14 | 20 | 30 | 30 | 30 | 40 | 30 | 20 |
| 15 |  |  |  |  |  |  |  |
| 16 |  |  |  |  |  |  |  |
| 17 |  |  |  |  |  |  |  |
| 18 |  |  |  |  |  |  |  |
| 19 | 5 | 5 | 5 | 0 | 0 | 0 | 5 |
| 20 | 25 |  | 25 |  | 25 |  |  |
| 21 |  |  |  |  |  |  |  |
| 22 |  |  |  |  |  |  |  |
| 23 |  |  |  |  |  |  |  |
| 24 | 20 | 80 | 50 | 25 | 25 | 25 | 10 |
| 25 | 40 | 30 | 30 | 10 | 40 | 30 | 20 |
| 26 | 70 | 70 | 80 | 80 | 100 | 100 | 100 |
| 27 |  |  |  |  |  |  |  |
| 28 | 50 | 60 | 70 | 70 | 50 | 70 | 50 |
| 29 |  |  |  |  |  |  |  |
| 30 | 30 | 30 |  |  |  |  |  |
| 31 | 50 | 60 | 60 | 70 | 70 | 65 | 70 |
| 32 |  |  |  |  |  |  |  |
| 33 | 45 | 50 | 50 | 40 | 40 | 40 | 20 |

Table 11: Physical Performance Parameters rated by importance for the different career stages of an astronaut – Strength Endurance Performance Lower Extremity

| ID | Strength Endurance Performance Lower Extremity [Astronaut Recruitment Process] | Strength Endurance Performance Lower Extremity [Astronaut Basic Training] | Strength Endurance Performance Lower Extremity [Mission Preparation] | Strength Endurance Performance Lower Extremity [Launch] | Strength Endurance Performance Lower Extremity [In Flight] | Strength Endurance Performance Lower Extremity [Landing Process] | Strength Endurance Performance Lower Extremity [Post Flight] |
| --- | --- | --- | --- | --- | --- | --- | --- |
| 6 |  |  |  |  |  |  |  |
| 7 | 90 | 80 | 85 | 30 | 30 | 30 | 70 |
| 8 | 0 | 20 | 30 | 30 | 10 | 50 | 70 |
| 9 | 50 | 60 | 70 | 70 | 60 | 60 | 60 |
| 10 |  |  |  |  |  |  |  |
| 11 |  |  |  |  |  |  |  |
| 12 | 60 | 50 | 60 | 25 | 60 | 25 | 60 |
| 13 |  |  |  |  |  |  |  |
| 14 | 35 | 50 | 50 | 50 | 10 | 50 | 40 |
| 15 |  |  |  |  |  |  |  |
| 16 |  |  |  |  |  |  |  |
| 17 |  |  |  |  |  |  |  |
| 18 |  |  |  |  |  |  |  |
| 19 | 10 | 10 | 10 | 0 | 0 | 0 | 10 |
| 20 | 25 | 25 | 25 | 25 | 25 | 25 | 75 |
| 21 |  |  |  |  |  |  |  |
| 22 |  |  |  |  |  |  |  |
| 23 |  |  |  |  |  |  |  |
| 24 | 20 | 80 | 40 | 25 | 25 | 25 | 10 |
| 25 | 60 | 60 | 80 | 30 | 70 | 30 | 30 |
| 26 | 70 | 70 | 80 | 80 | 100 | 100 | 100 |
| 27 |  |  |  |  |  |  |  |
| 28 | 50 | 60 | 60 | 60 | 50 | 60 | 50 |
| 29 |  |  |  |  |  |  |  |
| 30 | 50 | 50 |  |  |  |  |  |
| 31 | 60 | 65 | 70 | 75 | 80 | 80 | 80 |
| 32 |  |  |  |  |  |  |  |
| 33 | 70 | 75 | 75 | 40 | 40 | 40 | 20 |

Table 12: Physical Performance Parameters rated by importance for the different career stages of an astronaut – Maximum Strength CORE

| ID | Maximum Strength CORE [Astronaut Recruitment Process] | Maximum Strength CORE [Astronaut Basic Training] | Maximum Strength CORE [Mission Preparation] | Maximum Strength CORE [Launch] | Maximum Strength CORE [In Flight] | Maximum Strength CORE [Landing Process] | Maximum Strength CORE [Post Flight] |
| --- | --- | --- | --- | --- | --- | --- | --- |
| 6 | 50 | 50 | 50 | 30 | 50 | 50 | 30 |
| 7 | 85 | 85 | 75 | 50 | 50 | 65 | 85 |
| 8 | 0 | 20 | 30 | 30 | 30 | 20 | 50 |
| 9 | 60 | 70 | 70 | 70 | 65 | 60 | 60 |
| 10 |  |  |  |  |  |  |  |
| 11 |  |  |  |  |  |  |  |
| 12 | 85 | 65 | 85 | 65 | 85 | 65 | 65 |
| 13 |  |  |  |  |  |  |  |
| 14 | 25 | 35 | 50 | 40 | 50 | 35 | 20 |
| 15 |  |  |  |  |  |  |  |
| 16 |  |  |  |  |  |  |  |
| 17 |  |  |  |  |  |  |  |
| 18 |  |  |  |  |  |  |  |
| 19 | 15 | 15 | 10 | 5 | 5 | 5 | 15 |
| 20 |  |  | 25 |  |  |  | 25 |
| 21 |  |  |  |  |  |  |  |
| 22 |  |  |  |  |  |  |  |
| 23 |  |  |  |  |  |  |  |
| 24 | 20 | 80 | 40 | 25 | 25 | 25 | 10 |
| 25 | 40 | 40 | 40 | 50 | 70 | 20 | 10 |
| 26 | 70 | 70 | 80 | 80 | 100 | 100 | 100 |
| 27 |  |  |  |  |  |  |  |
| 28 | 50 | 60 | 70 | 60 | 70 | 60 | 60 |
| 29 |  |  |  |  |  |  |  |
| 30 | 50 | 50 |  |  |  |  |  |
| 31 | 60 | 65 | 70 | 80 | 80 | 80 | 80 |
| 32 |  |  |  | 80 |  | 80 |  |
| 33 | 60 | 65 | 65 | 50 | 65 | 65 | 20 |

Table 13: Physical Performance Parameters rated by importance for the different career stages of an astronaut – Strength Endurance Performance CORE

| ID | Strength Endurance Performance CORE [Astronaut Recruitment Process] | Strength Endurance Performance CORE [Astronaut Basic Training] | Strength Endurance Performance CORE [Mission Preparation] | Strength Endurance Performance CORE [Launch] | Strength Endurance Performance CORE [In Flight] | Strength Endurance Performance CORE [Landing Process] | Strength Endurance Performance CORE [Post Flight] |
| --- | --- | --- | --- | --- | --- | --- | --- |
| 6 | 50 | 50 | 50 | 30 | 50 | 50 | 30 |
| 7 | 70 | 70 | 70 | 70 | 50 | 70 | 70 |
| 8 | 0 | 20 | 30 | 30 | 30 | 60 | 70 |
| 9 | 70 | 80 | 85 | 85 | 70 | 70 | 60 |
| 10 |  |  |  |  |  |  |  |
| 11 |  |  |  |  |  |  |  |
| 12 | 85 | 65 | 85 | 65 | 85 | 65 | 65 |
| 13 |  |  |  |  |  |  |  |
| 14 | 30 | 40 | 50 | 40 | 50 | 40 | 35 |
| 15 |  |  |  |  |  |  |  |
| 16 |  |  |  |  |  |  |  |
| 17 |  |  |  |  |  |  |  |
| 18 |  |  |  |  |  |  |  |
| 19 | 50 | 40 | 40 | 10 | 10 | 10 | 20 |
| 20 |  |  | 75 |  |  |  | 90 |
| 21 |  |  |  |  |  |  |  |
| 22 |  |  |  |  |  |  |  |
| 23 |  |  |  |  |  |  |  |
| 24 | 20 | 80 | 40 | 25 | 25 | 25 | 10 |
| 25 | 30 | 30 | 30 | 20 | 50 | 20 | 10 |
| 26 | 70 | 70 | 80 | 80 | 100 | 100 | 100 |
| 27 |  |  |  |  |  |  |  |
| 28 | 50 | 60 | 70 | 70 | 70 | 70 | 70 |
| 29 |  |  |  |  |  |  |  |
| 30 | 50 | 50 |  |  |  |  |  |
| 31 | 65 | 70 | 80 | 85 | 85 | 85 | 100 |
| 32 |  |  |  | 80 |  | 80 |  |
| 33 | 80 | 90 | 90 | 50 | 90 | 80 | 20 |

Table 14: Physical Performance Parameters rated by importance for the different career stages of an astronaut – Balance

| ID | Balance [Astronaut Recruitment Process] | Balance [Astronaut Basic Training] | Balance [Mission Preparation] | Balance [Launch] | Balance [In Flight] | Balance [Landing Process] | Balance [Post Flight] |
| --- | --- | --- | --- | --- | --- | --- | --- |
| 6 | 100 | 100 | 100 | 50 | 100 | 50 | 80 |
| 7 | 50 | 50 | 60 | 60 | 40 | 40 | 85 |
| 8 | 30 | 30 | 50 | 50 | 0 | 70 | 70 |
| 9 | 65 | 75 | 80 | 85 | 50 | 60 | 70 |
| 10 |  |  |  |  |  |  |  |
| 11 |  |  |  |  |  |  |  |
| 12 | 70 | 25 | 70 | 25 | 70 | 25 | 25 |
| 13 |  |  |  |  |  |  |  |
| 14 | 60 | 60 | 60 | 60 | 20 | 50 | 70 |
| 15 |  |  |  |  |  |  |  |
| 16 |  |  |  |  |  |  |  |
| 17 |  |  |  |  |  |  |  |
| 18 |  |  |  |  |  |  |  |
| 19 | 40 | 30 | 30 | 5 | 5 | 5 | 50 |
| 20 |  |  |  |  |  |  | 100 |
| 21 |  |  |  |  |  |  |  |
| 22 |  |  |  |  |  |  |  |
| 23 |  |  |  |  |  |  |  |
| 24 | 20 | 80 | 40 | 25 | 25 | 25 | 10 |
| 25 | 90 | 90 | 90 | 60 | 30 | 90 | 75 |
| 26 | 70 | 80 | 80 | 80 | 100 | 100 | 100 |
| 27 |  |  |  |  |  |  |  |
| 28 | 50 | 60 | 60 | 60 | 60 | 60 | 70 |
| 29 |  |  |  |  |  |  |  |
| 30 | 70 | 70 |  |  |  |  |  |
| 31 | 60 | 60 | 60 | 65 | 45 | 45 | 100 |
| 32 |  |  |  |  |  |  |  |
| 33 | 75 | 85 | 85 | 50 | 0 | 85 | 85 |

Table 15: Physical Performance Parameters rated by importance for the different career stages of an astronaut – Dexterity Upper Extremity

| ID | Dexterity Upper Extremity [Astronaut Recruitment Process] | Dexterity Upper Extremity [Astronaut Basic Training] | Dexterity Upper Extremity [Mission Preparation] | Dexterity Upper Extremity [Launch] | Dexterity Upper Extremity [In Flight] | Dexterity Upper Extremity [Landing Process] | Dexterity Upper Extremity [Post Flight] |
| --- | --- | --- | --- | --- | --- | --- | --- |
| 6 | 100 | 100 | 100 | 50 | 50 | 50 | 80 |
| 7 | 65 | 65 | 65 | 65 | 65 | 40 | 90 |
| 8 | 50 | 55 | 55 | 70 | 70 | 40 | 20 |
| 9 | 80 | 90 | 100 | 90 | 80 | 80 | 75 |
| 10 |  |  |  |  |  |  |  |
| 11 |  |  |  |  |  |  |  |
| 12 | 85 | 50 | 85 | 70 | 85 | 70 | 50 |
| 13 |  |  |  |  |  |  |  |
| 14 | 70 | 60 | 70 | 60 | 60 | 60 | 70 |
| 15 |  |  |  |  |  |  |  |
| 16 |  |  |  |  |  |  |  |
| 17 |  |  |  |  |  |  |  |
| 18 |  |  |  |  |  |  |  |
| 19 | 60 | 60 | 60 | 10 | 10 | 10 | 30 |
| 20 | 100 | 100 | 100 | 100 | 100 | 100 | 75 |
| 21 |  |  |  |  |  |  |  |
| 22 |  |  |  |  |  |  |  |
| 23 |  |  |  |  |  |  |  |
| 24 | 20 | 80 | 40 | 25 | 25 | 25 | 10 |
| 25 | 80 | 60 | 70 | 50 | 80 | 60 | 50 |
| 26 | 70 | 70 | 80 | 80 | 80 | 100 | 100 |
| 27 |  |  |  |  |  |  |  |
| 28 | 60 | 60 | 70 | 70 | 70 | 70 | 50 |
| 29 |  |  |  |  |  |  |  |
| 30 | 30 | 25 |  |  |  |  |  |
| 31 | 60 | 60 | 60 | 65 | 55 | 60 | 75 |
| 32 |  |  |  |  |  |  |  |
| 33 | 85 | 90 | 90 | 90 | 90 | 90 | 20 |

Table 16: Physical Performance Parameters rated by importance for the different career stages of an astronaut – Dexterity Lower Extremity

| ID | Dexterity Lower Extremity [Astronaut Recruitment Process] | Dexterity Lower Extremity [Astronaut Basic Training] | Dexterity Lower Extremity [Mission Preparation] | Dexterity Lower Extremity [Launch] | Dexterity Lower Extremity [In Flight] | Dexterity Lower Extremity [Landing Process] | Dexterity Lower Extremity [Post Flight] |
| --- | --- | --- | --- | --- | --- | --- | --- |
| 6 | 0 | 0 | 0 | 0 | 0 | 0 | 0 |
| 7 | 65 | 65 | 65 | 65 | 65 | 65 | 70 |
| 8 | 20 | 25 | 25 | 25 | 25 | 20 | 20 |
| 9 | 60 | 70 | 75 | 70 | 65 | 60 | 60 |
| 10 |  |  |  |  |  |  |  |
| 11 |  |  |  |  |  |  |  |
| 12 | 0 | 0 | 0 | 0 | 5 | 0 | 0 |
| 13 |  |  |  |  |  |  |  |
| 14 | 30 | 30 | 40 | 30 | 10 | 20 | 50 |
| 15 |  |  |  |  |  |  |  |
| 16 |  |  |  |  |  |  |  |
| 17 |  |  |  |  |  |  |  |
| 18 |  |  |  |  |  |  |  |
| 19 | 5 | 5 | 5 | 0 | 0 | 0 | 5 |
| 20 | 10 |  |  |  |  |  |  |
| 21 |  |  |  |  |  |  |  |
| 22 |  |  |  |  |  |  |  |
| 23 |  |  |  |  |  |  |  |
| 24 | 20 | 80 | 40 | 25 | 25 | 25 | 10 |
| 25 | 20 | 20 | 20 | 10 | 20 | 10 | 10 |
| 26 | 70 | 70 | 80 | 80 | 100 | 100 | 100 |
| 27 |  |  |  |  |  |  |  |
| 28 | 40 | 50 | 50 | 50 | 50 | 50 | 60 |
| 29 |  |  |  |  |  |  |  |
| 30 | 30 | 35 |  |  |  |  |  |
| 31 | 60 | 60 | 60 | 65 | 45 | 65 | 100 |
| 32 |  |  |  |  |  |  |  |
| 33 | 85 | 70 | 70 | 70 | 70 | 70 | 70 |

Table 17: Physical Performance Parameters rated by importance for the different career stages of an astronaut – Coordination Upper Extremity

| ID | Coordination Upper Extremity [Astronaut Recruitment Process] | Coordination Upper Extremity [Astronaut Basic Training] | Coordination Upper Extremity [Mission Preparation] | Coordination Upper Extremity [Launch] | Coordination Upper Extremity [In Flight] | Coordination Upper Extremity [Landing Process] | Coordination Upper Extremity [Post Flight] |
| --- | --- | --- | --- | --- | --- | --- | --- |
| 6 | 100 | 100 | 100 | 100 | 100 | 100 | 80 |
| 7 | 95 | 95 | 95 | 95 | 95 | 95 | 100 |
| 8 | 70 | 75 | 75 | 75 | 75 | 40 | 50 |
| 9 | 80 | 80 | 90 | 90 | 80 | 80 | 80 |
| 10 |  |  |  |  |  |  |  |
| 11 |  |  |  |  |  |  |  |
| 12 | 85 | 65 | 85 | 65 | 85 | 65 | 65 |
| 13 |  |  |  |  |  |  |  |
| 14 | 60 | 50 | 60 | 60 | 70 | 50 | 70 |
| 15 |  |  |  |  |  |  |  |
| 16 |  |  |  |  |  |  |  |
| 17 |  |  |  |  |  |  |  |
| 18 |  |  |  |  |  |  |  |
| 19 | 75 | 75 | 60 | 35 | 40 | 10 | 40 |
| 20 | 100 | 100 | 100 | 100 | 100 | 100 | 75 |
| 21 |  |  |  |  |  |  |  |
| 22 |  |  |  |  |  |  |  |
| 23 |  |  |  |  |  |  |  |
| 24 | 20 | 80 | 40 | 25 | 25 | 25 | 10 |
| 25 | 80 | 80 | 80 | 90 | 100 | 75 | 50 |
| 26 | 70 | 70 | 80 | 100 | 100 | 100 | 100 |
| 27 |  |  |  |  |  |  |  |
| 28 | 50 | 50 | 60 | 60 | 60 | 60 | 60 |
| 29 |  |  |  |  |  |  |  |
| 30 | 50 | 50 |  |  |  |  |  |
| 31 | 60 | 60 | 60 | 65 | 45 | 65 | 75 |
| 32 |  |  |  |  |  |  |  |
| 33 | 85 | 90 | 90 | 90 | 90 | 90 | 50 |

Table 18: Physical Performance Parameters rated by importance for the different career stages of an astronaut – Coordination Lower Extremity

| ID | Coordination Lower Extremity [Astronaut Recruitment Process] | Coordination Lower Extremity [Astronaut Basic Training] | Coordination Lower Extremity [Mission Preparation] | Coordination Lower Extremity [Launch] | Coordination Lower Extremity [In Flight] | Coordination Lower Extremity [Landing Process] | Coordination Lower Extremity [Post Flight] |
| --- | --- | --- | --- | --- | --- | --- | --- |
| 6 | 0 | 0 | 0 | 0 | 0 | 0 | 0 |
| 7 | 95 | 95 | 95 | 95 | 95 | 85 | 95 |
| 8 | 20 | 30 | 30 | 30 | 30 | 10 | 40 |
| 9 | 60 | 70 | 75 | 70 | 65 | 60 | 70 |
| 10 |  |  |  |  |  |  |  |
| 11 |  |  |  |  |  |  |  |
| 12 | 60 | 55 | 60 | 55 | 60 | 55 | 55 |
| 13 |  |  |  |  |  |  |  |
| 14 | 40 | 50 | 50 | 60 | 20 | 40 | 70 |
| 15 |  |  |  |  |  |  |  |
| 16 |  |  |  |  |  |  |  |
| 17 | 50 | 75 | 75 | 75 | 75 | 75 | 100 |
| 18 |  |  |  |  |  |  |  |
| 19 | 10 | 10 | 10 | 5 | 10 | 5 | 10 |
| 20 | 25 |  |  |  |  |  |  |
| 21 |  |  |  |  |  |  |  |
| 22 |  |  |  |  |  |  |  |
| 23 |  |  |  |  |  |  |  |
| 24 | 20 | 80 | 40 | 25 | 25 | 25 | 10 |
| 25 | 20 | 20 | 30 | 20 | 40 | 20 | 20 |
| 26 | 70 | 70 | 80 | 100 | 100 | 100 | 100 |
| 27 |  |  |  |  |  |  |  |
| 28 | 50 | 50 | 60 | 60 | 60 | 60 | 60 |
| 29 |  |  |  |  |  |  |  |
| 30 | 50 | 50 |  |  |  |  |  |
| 31 | 60 | 60 | 60 | 65 | 45 | 70 | 100 |
| 32 |  |  |  |  |  |  |  |
| 33 | 85 | 90 | 90 | 70 | 70 | 70 | 70 |

Table 19: Physical Performance Parameters rated by importance for the different career stages of an astronaut – Mobility and Flexibility

| ID | Mobility and Flexibility (passive and active Range of Joint Motion) [Astronaut Recruitment Process] | Mobility and Flexibility (passive and active Range of Joint Motion) [Astronaut Basic Training] | Mobility and Flexibility (passive and active Range of Joint Motion) [Mission Preparation] | Mobility and Flexibility (passive and active Range of Joint Motion) [Launch] | Mobility and Flexibility (passive and active Range of Joint Motion) [In Flight] | Mobility and Flexibility (passive and active Range of Joint Motion) [Landing Process] | Mobility and Flexibility (passive and active Range of Joint Motion) [Post Flight] |
| --- | --- | --- | --- | --- | --- | --- | --- |
| 6 | 70 | 70 | 70 | 50 | 70 | 50 | 70 |
| 7 | 80 | 80 | 80 | 80 | 60 | 80 | 90 |
| 8 | 0 | 10 | 50 | 60 | 50 | 50 | 20 |
| 9 | 70 | 80 | 80 | 80 | 70 | 60 | 70 |
| 10 |  |  |  |  |  |  |  |
| 11 |  |  |  |  |  |  |  |
| 12 | 50 | 50 | 50 | 70 | 50 | 50 | 50 |
| 13 |  |  |  |  |  |  |  |
| 14 | 65 | 65 | 75 | 75 | 75 | 75 | 40 |
| 15 |  |  |  |  |  |  |  |
| 16 |  |  |  |  |  |  |  |
| 17 | 50 | 75 | 75 | 75 | 100 | 75 | 100 |
| 18 |  |  |  |  |  |  |  |
| 19 | 40 | 40 | 40 | 20 | 30 | 5 | 20 |
| 20 | 90 | 90 | 90 | 90 | 90 | 90 | 50 |
| 21 |  |  |  |  |  |  |  |
| 22 |  |  |  |  |  |  |  |
| 23 |  |  |  |  |  |  |  |
| 24 | 20 | 80 | 40 | 25 | 25 | 25 | 10 |
| 25 | 70 | 70 | 75 | 40 | 75 | 20 | 10 |
| 26 | 70 | 70 | 80 | 100 | 100 | 100 | 100 |
| 27 |  |  |  |  |  |  |  |
| 28 | 50 | 60 | 70 | 70 | 50 | 70 | 60 |
| 29 |  |  |  |  |  |  |  |
| 30 | 50 | 50 |  |  |  |  |  |
| 31 | 50 | 55 | 60 | 60 | 60 | 60 | 60 |
| 32 |  |  |  |  |  |  |  |
| 33 | 70 | 75 | 75 | 75 | 75 | 75 | 75 |

Table 20: Physical Performance Parameters rated by importance for the different career stages of an astronaut – Agility

| ID | Agility (e.g., fast changes of direction, ability quickly to overcome obstacles) [Astronaut Recruitment Process] | Agility (e.g., fast changes of direction, ability quickly to overcome obstacles) [Astronaut Basic Training] | Agility (e.g., fast changes of direction, ability quickly to overcome obstacles) [Mission Preparation] | Agility (e.g., fast changes of direction, ability quickly to overcome obstacles) [Launch] | Agility (e.g., fast changes of direction, ability quickly to overcome obstacles) [In Flight] | Agility (e.g., fast changes of direction, ability quickly to overcome obstacles) [Landing Process] | Agility (e.g., fast changes of direction, ability quickly to overcome obstacles) [Post Flight] |
| --- | --- | --- | --- | --- | --- | --- | --- |
| 6 | 50 | 50 | 50 | 30 | 30 | 30 | 50 |
| 7 | 90 | 90 | 90 | 90 | 90 | 90 | 90 |
| 8 | 0 | 10 | 10 | 10 | 0 | 0 | 0 |
| 9 | 70 | 75 | 75 | 75 | 65 | 70 | 70 |
| 10 |  |  |  |  |  |  |  |
| 11 |  |  |  |  |  |  |  |
| 12 | 50 | 35 | 50 | 10 | 50 | 10 | 50 |
| 13 |  |  |  |  |  |  |  |
| 14 | 30 | 40 | 40 | 30 | 50 | 30 | 60 |
| 15 |  |  |  |  |  |  |  |
| 16 |  |  |  |  |  |  |  |
| 17 | 50 | 75 | 75 | 75 | 100 | 75 | 100 |
| 18 |  |  |  |  |  |  |  |
| 19 | 60 | 40 | 40 | 10 | 40 | 5 | 15 |
| 20 | 60 | 60 | 60 | 60 | 60 | 100 | 80 |
| 21 |  |  |  |  |  |  |  |
| 22 |  |  |  |  |  |  |  |
| 23 |  |  |  |  |  |  |  |
| 24 | 20 | 80 | 40 | 25 | 25 | 25 | 10 |
| 25 | 60 | 60 | 70 | 20 | 75 | 30 | 20 |
| 26 | 70 | 70 | 80 | 100 | 100 | 100 | 100 |
| 27 |  |  |  |  |  |  |  |
| 28 | 50 | 60 | 70 | 70 | 60 | 70 | 70 |
| 29 |  |  |  |  |  |  |  |
| 30 | 70 | 70 |  |  |  |  |  |
| 31 | 50 | 55 | 60 | 50 | 45 | 55 | 85 |
| 32 |  |  |  |  |  |  |  |
| 33 | 65 | 70 | 70 | 40 | 90 | 70 | 70 |

Table 21: Raw data of the open question of Delphi round 2 addressing needs and requirements for implementing Physical Performance Tests in any other relevant context that has not been captured through previous questions

| **ID** | **Are there any nominal or off-nominal crew activities or situations that should require a specific physical performance and/or motor skills test to assess individual crew readiness?** | **Are there any nominal or off-nominal crew activities or situations that should require a specific physical performance and/or motor skills test to assess individual crew readiness? [Comment]** |
| --- | --- | --- |
| 1 | Not sure |  |
| 2 | No |  |
| 3 | Yes | Yes, EAV training and performance needs special training to avoid overstressing the motion apparatus. The survival training may require previous demonstrations of endurance and exhaustion consequences. I knew my limits after the sea survival exercise in a hot bobbling Soyuz spacecraft. It was one of the most exhaustive all-body experiences I ever had. It would have been good to go through parts of it beforehand. |
| 4 | Yes | Probably in off nominal situation may require specific capabilities but in total a good combination of motor skills, and balance strength may be relevant Each one being different we might use the best power of each one in a dedicated manner... training the team together may be very useful to improve capabilities of the team |
| 5 |  |  |
| 6 | Not sure |  |
| 7 | Yes | EVAs Robotics |
| 8 |  |  |
| 9 | Yes | Spacewalks, a.k.a. EVAs need the astronaut to be fit, enduring and strong enough to perform tiresome activities over a prolonged period of time. Launch and landing may impose loads on the body which fitness may be able to counteract. In the event of a launch or landing failure, the crew may end up in a remote area and will need to be fit to survive and built a shelter until rescued (if the capsule lands in the water, this may include swimming). |
| 10 |  |  |
| 11 |  |  |
| 12 | Yes | e.g., inflight: if EVAs are planned endurance, agility, mobility extremely important, as well as visual and hearing abilities |
| 13 |  |  |
| 14 | Yes | Evacuation, e.g., water egress exercise. |
| 15 | Yes | depends what mission tasks crew is assigned to, for example EVA and or robotic arm |
| 16 |  |  |
| 17 |  |  |
| 18 |  |  |
| 19 | Not sure |  |
| 20 | Yes | - putting on and off space suits requires some agility - manual flying of robotic systems requires good psychomotricity - repair of delicate equipment requires dexterity - spacewalk requires both cardio endurance and upper body strength - emergency evacuation requires upper body tonicity for quick actions - in case of emergency landing and/or survival, overall good physical and psychological condition are required (lower body not as important as upper body) - survival was not included in the previous question but should be also taken into account for defining importance of physical abilities required in various phases of an astronaut career. |
| 21 | Yes | Space vehicle nominal and emergency exiting/evacuation at launch and landing are likely events which require specific physical performance and if trained and performed effectively are key risk mitigation manoeuvres |
| 22 |  |  |
| 23 | No |  |
| 24 |  |  |
| 25 | Yes | seeking shelter, various disaster scenarios (fire, smoke, depress) and emergency egress |
| 26 | No |  |
| 27 |  |  |
| 28 | Not sure |  |
